# Facile access to potent antiviral quinazoline heterocycles with fluorescence properties via merging metal-free domino reactions

**DOI:** 10.1038/ncomms15071

**Published:** 2017-05-02

**Authors:** Felix E. Held, Anton A. Guryev, Tony Fröhlich, Frank Hampel, Axel Kahnt, Corina Hutterer, Mirjam Steingruber, Hanife Bahsi, Clemens von Bojničić-Kninski, Daniela S. Mattes, Tobias C. Foertsch, Alexander Nesterov-Mueller, Manfred Marschall, Svetlana B. Tsogoeva

**Affiliations:** 1 Organic Chemistry Chair I and Interdisciplinary Center for Molecular Materials, Friedrich-Alexander University of Erlangen-Nürnberg, Henkestrasse 42, 91054 Erlangen, Germany; 2 Physical Chemistry Chair I, Friedrich-Alexander University of Erlangen-Nürnberg, Egerlandstrasse 3, 91058 Erlangen, Germany; 3 Institute for Clinical and Molecular Virology, Friedrich-Alexander University of Erlangen-Nürnberg, Schlossgarten 4, 91054 Erlangen, Germany; 4 Karlsruhe Institute of Technology, Institute of Microstructure Technology, Hermann-von-Helmholtz-Platz 1, 76344 Eggenstein-Leopoldshafen, Germany

## Abstract

Most of the known approved drugs comprise functionalized heterocyclic compounds as subunits. Among them, non-fluorescent quinazolines with four different substitution patterns are found in a variety of clinically used pharmaceuticals, while 4,5,7,8-substituted quinazolines and those displaying their own specific fluorescence, favourable for cellular uptake visualization, have not been described so far. Here we report the development of a one-pot synthetic strategy to access these 4,5,7,8-substituted quinazolines, which are fluorescent and feature strong antiviral properties (**EC**_**50**_ down to 0.6±0.1 μM) against human cytomegalovirus (HCMV). Merging multistep domino processes in one-pot under fully metal-free conditions leads to sustainable, maximum efficient and high-yielding organic synthesis. Furthermore, generation of artesunic acid–quinazoline hybrids and their application against HCMV (**EC**_**50**_ down to 0.1±0.0 μM) is demonstrated. Fluorescence of new antiviral hybrids and quinazolines has potential applications in molecular imaging in drug development and mechanistic studies, avoiding requirement of linkage to external fluorescent markers.

## Abstract (web summary)

Heterocycles are ubiquitous in bioactive compounds and routes to different substitution patterns are important to access the full substrate space. Here the authors report a route to 4,5,7,8-substituted antiviral fluorescent quinazolines, to allow cellular uptake visualization without external marker.

Quinazoline heterocycles are ubiquitous in pharmaceutical compounds and drugs. They are important subunits of a broad variety of natural products as well as synthetic pharmaceuticals possessing anti-inflammatory^1^, antiviral^2^, antimalarial^3^ and anticancer^4^ activities. The known quinazoline-based drugs, which are not demonstrating fluorescence properties, can be divided into four groups **A**–**D** (2,4,6,7-; 4,6,7-; 2,4,5,6- and 2,4,6,7,8-substituted quinazolines), representing four types of existing substitution patterns (Fig. 1a).

The synthesis of known quinazoline heterocycles by conventional ways is a hard work that implies many synthetic steps and expensive starting materials, and involves time-consuming and waste-producing isolation and purification of product intermediates^4^,^5^,^6^,^7^,^8^. The number of synthetic methods to afford quinazolines is, furthermore, restricted to the availability of the appropriate starting compounds. For this reason, development of new efficient and straightforward synthetic methods towards quinazoline heterocyclic compounds of value to medicinal chemistry, starting from simple precursors, is of high current demand. To our knowledge, the class of 4,5,7,8-substituted quinazolines (**E**-type, Fig. 1b) have not been prepared to date because of the lack of a synthetic route towards these compounds. Furthermore, no examples of fluorescent quinazolines have been reported so far. External fluorescent labels are usually employed to study the functions of quinazoline-based pharmacophores within cells. However, fluorescent markers can influence or change the properties of studied lead compounds and drugs. Therefore, the drug candidate should ideally display its own fluorescence to allow its cellular uptake visualization and without incorporation of external fluorescent labels. The motivation and desire to address these gaps prompted the research work we report here.

Recently, combined processes, where several fundamentally different catalytic reactions are joined in a one-pot protocol, were introduced^9^. Among known examples are combinations of (i) organocatalysis and transition–metal catalysis^10^, (ii) organocatalysis and silver or gold catalysis^11^,^12^,^13^ or (iii) organocatalysis and (photo)redox catalysis^14^,^15^. Even more efficient and economical methods towards heterocyclic compounds are domino processes^16^,^17^,^18^,^19^,^20^,^21^,^22^.

In terms of efficiency and sustainability, generation of bioactive heterocycles through multicomponent multistep reactions avoiding intermediate isolation and purification steps, is unbeatable. While examples of organocatalysed linear domino reactions^17^,^18^,^20^ and of a single branched domino process^21^ are known, surprisingly, combining them in one-pot is unprecedented, although such a multistep one-pot process would further reduce costs and waste production in the synthesis of versatile heterocycles.

Herein, we describe development of a combined process, which joins a new five-step branched domino reaction with two-step linear and subsequent three-step linear domino reactions. This new metal-free 10-step sequence, with only a single work up procedure and starting from simple and readily available compounds, results in new functionalized quinazolines of type **E** (Fig. 1b), which exceed the antiviral potency of the clinical reference drug ganciclovir^23^. Furthermore, selected fluorescent quinazolines were applied for synthesis of first artesunic acid–quinazoline hybrids, which are, remarkably, also fluorescent and display superior potency against HCMV (**EC**_**50**_ down to 0.1±0.0 μM) compared to that of their parent quinazolines (**EC**_**50**_ down to 4.6±0.9 μM) and artesunic acid (**EC**_**50**_ 3.8±0.4 μM), as well as ganciclovir (**EC**_**50**_ 2.6±0.5 μM). For all quinazoline compounds, cytotoxicity for primary human fibroblasts (HFFs; **CC**_**50**_) was undetectable at concentrations up to 100 μM, indicating that the new quinazolines and hybrid compounds are selective. Importantly, the fluorescent quinazolines could nicely be depicted both in extracellular and intracellular localizations when analysing primary HFFs and virus-infected cells using confocal laser-scanning microscopy. An accumulation of fluorescent quinazolines was mostly observed in cytoplasmic areas, thus visually indicating their efficient cellular uptake. These results open up new perspectives for molecular imaging in the drug development process and mechanistic studies, avoiding the requirement of linkage of external fluorescent labels to potential drug molecules.

## Results

### Proposed one-pot approach towards fluorescent quinazolines

**Figure 2:**
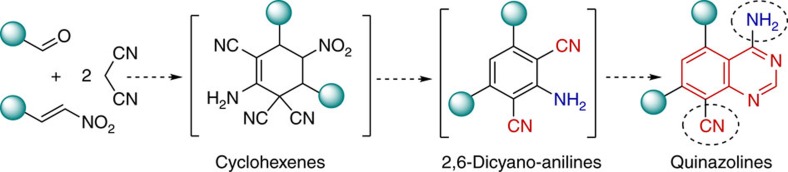
Proposed convenient synthesis of fluorescent quinazolines. One-pot multistep synthetic approach towards 4,5,7,8-substituted quinazolines from simple compounds.

Inspired by the power of the domino concept introduced by Tietze^16^,^22^, we envisioned the possibility of designing a multicomponent one-pot process, involving domino reactions, which might allow to generate quinazolines starting from simple precursors: aldehydes, nitroalkenes and malononitrile (Fig. 2). The proposed unprecedented quinazolines, bearing amino (donor) and cyano (acceptor) groups at the same time, were expected to demonstrate desirable fluorescence properties.

### One-pot synthesis of cyclohexenes and 2,6-dicyanoanilines

**Figure 3:**
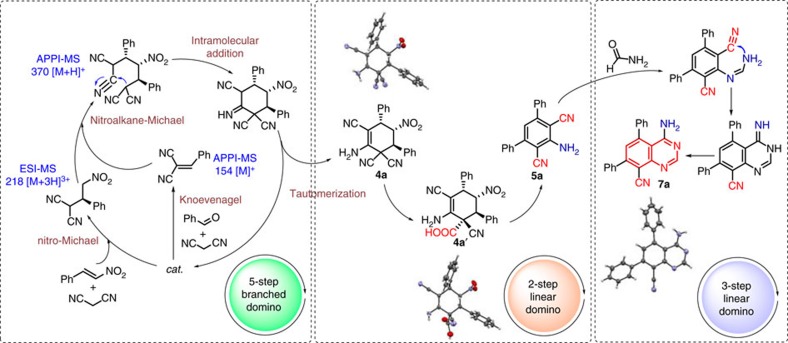
Merging metal-free domino reactions. Development of one-pot access to new 2,6-dicyanoaniline-based multichromophores and 4,5,7,8-substituted quinazolines. For detailed X-ray data of compounds **4a**, **4a′** and **7a**, see Supplementary Tables 2–4.

**Figure 4:**
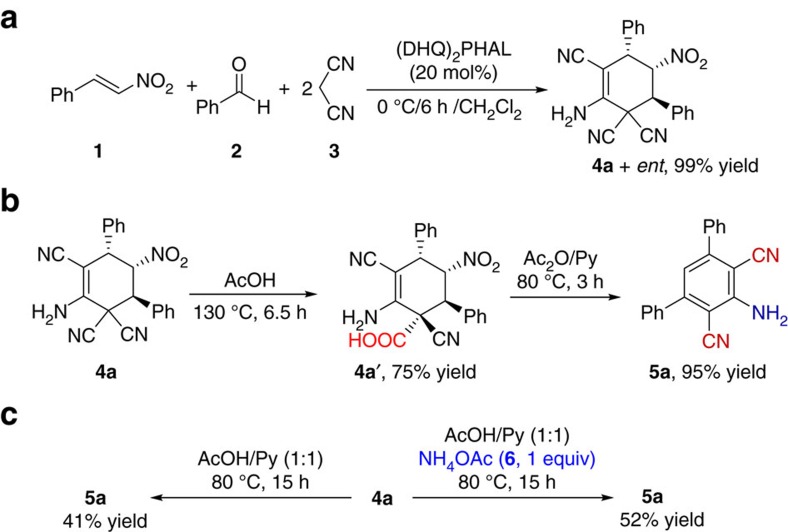
Optimization of domino and one-pot processes. (**a**) Four-component (ABC_2_) five-step branched domino reaction. (**b**,**c**) Optimization of one-pot two-step synthesis of 2,6-dicyanoaniline derivative **5a** (see Supplementary Methods).

We started by exploring a reaction of *trans*-*β*-nitrostyrene (**1**), benzaldehyde (**2**) and malononitrile (**3**; Figs 3 and 4a). Our most recent finding of an imidazole-catalysed six-step linear domino reaction of malononitrile with phenylethanal derivatives^24^ inspired us to apply imidazole as a catalyst also for this new transformation (Supplementary Fig. 1). To our delight, the domino process using dichloromethane as a solvent in presence of imidazole at room temperature resulted in highly functionalized cyclohexene derivative **4a**. Structure and relative stereochemistry of isolated major diastereomer **4a** was confirmed by X-ray analysis (Fig. 3).

We next screened further commercially available bases (Supplementary Fig. 1). A significant improvement of product yields to >99% was observed in presence of hydroquinine 1,4-phthalazinediyl diether ((DHQ)_2_PHAL, Fig. 4a), well known as a ligand in osmium-catalysed Sharpless dihydroxylation reaction^25^.

The proposed mechanism of the branched domino reaction was confirmed by ESI- and APPI-MS studies (Fig. 3). In presence of a catalyst, we observed signals at *m/z* 154 [M]^+^ and *m/z* 218 [M+3H]^3+^, which correspond to products of two transformations running in parallel, Knoevenagel (step 1) and nitro-Michael (step 2) reactions, respectively. A signal at *m/z* 370 [M+H]^+^ corresponds to product of a subsequent nitroalkane-Michael reaction (step 3) between previously formed nitroalkane derivative (donor) and a Knoevenagel product (Michael acceptor). The last steps, leading to target compound **4a**, involve intramolecular addition (step 4) and tautomerization (step 5).

Next, optimal conditions for 2,6-dicyanoaniline derivative **5a** formation (key intermediate for quinazoline synthesis according to Fig. 2) from cyclohexene **4a** were studied (see Supplementary Table 1). Notably, 2,6-dicyanoaniline derivatives have recently attracted considerable attention, since they exhibit strong fluorescence in ultraviolet light and may have utility in synthesis of fluorescent materials, non-natural photosynthetic systems, materials with semiconducting or nonlinear optical properties, molecular electronic devices and light-emitting diodes^26^,^27^,^28^,^29^.

Since direct aromatization of **4a** was not successful using different oxidizing agents (see Supplementary Table 1), we assumed that hydrolysis of CN groups to COOH might facilitate the subsequent aromatization step through CO_2_ release and might also prevent generation of toxic hydrogen cyanide (HCN). Applying acetic acid (AcOH) and carrying out the reaction at 130 °C for 6.5 h, product **4a′** with a quaternary carbon centre was isolated in 75% yield (Fig. 4b). Subsequently, we reacted **4a′** with Ac_2_O/Py, giving desired product **5a** in 95% yield. Next, we turned our attention to finding the optimal conditions for two-step one-pot synthesis of **5a** from **4a** (Fig. 4c). Carrying out the reaction in presence of AcOH/Py (1:1) at 80 °C for 15 h, desired product **5a** was isolated in 41% yield. We obtained, furthermore, performing the same reaction under otherwise identical conditions—but in presence of ammonium acetate (**6**) as an additive—the product **5a** in a higher yield of 52% over two steps (Fig. 4c).

**Figure 5:**
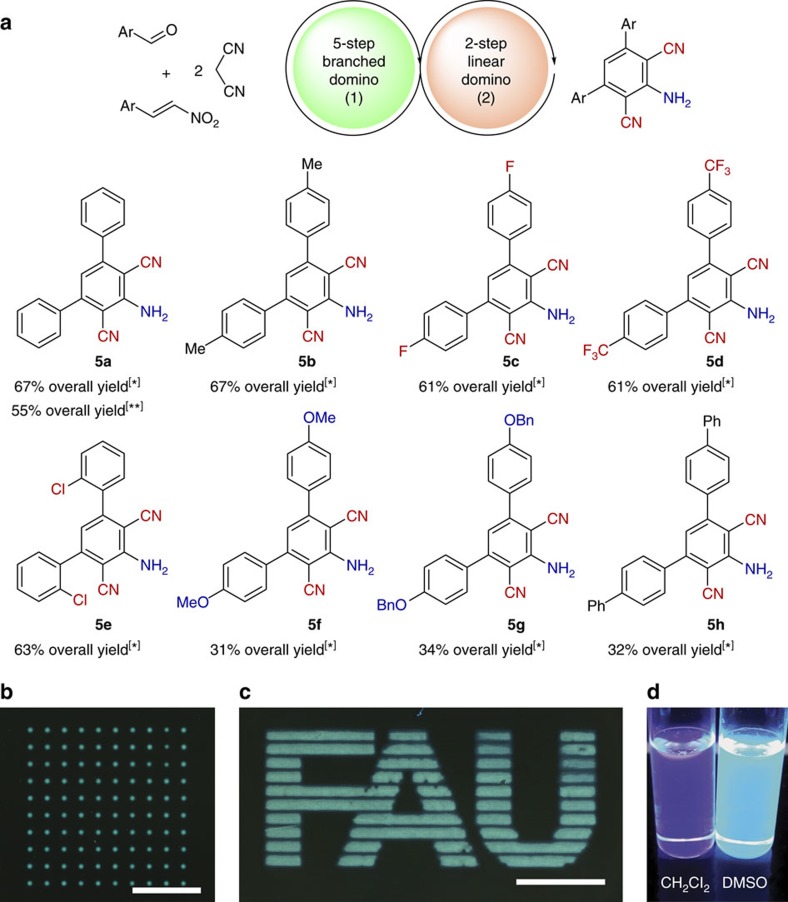
One-pot seven-step process and imaging experiments with 5d using combinatorial laser-induced forward transfer (cLIFT) method. (**a**) Synthesis of new 2,6-dicyanoaniline-based multichromophores via seven-step one-pot sequence. * (1) Catalyst (DHQ)_2_PHAL (5 mol%), CH_2_Cl_2_, RT, 3 h, (2) AcOH/Py=1/1, 1 eq. NH_4_OAc, 80 °C, 15 h. ** (1) Catalyst (DHQ)_2_PHAL (20 mol%), CH_2_Cl_2_, 0 °C, 6 h, (2) AcOH/Py=1/1, 1 eq. NH_4_OAc, 80 °C, 15 h (see Supplementary Methods). (**b**) Fluorescent image of an array (10 × 10 spots) of the selected new fluorophore **5d** patterned via cLIFT. Excited at 254 nm, the fluorescence was captured for 10 s at ISO 1,600 with a resolution of 1.25 μm per pixel (scale bar, 1 mm). (**c**) Imaging of Friedrich-Alexander University logo with the new fluorophore **5d** patterned on a glass slide using cLIFT (imaging parameters see **b**; scale bar, 2 mm), see Supplementary Fig. 2 and Supplementary Note 1. (**d**) Demonstration of the fluorescence properties of **5d** in two different solvents illuminated by ultraviolet light.

Another specific aim was to combine the five-step branched domino reaction with the optimized two-step linear domino reaction in a seven-step one-pot process, which we successfully realized (Fig. 5a). Noteworthy, all reactions investigated can be effected in 31–67% overall yield over seven steps. Generally, higher yields (61–67% for **5a**–**5e**) were obtained using substrates with both neutral and electron-withdrawing moieties on benzene rings.

### Laser-induced fluorescence imaging with 2,6-dicyanoaniline

Using the combinatorial laser-induced forward transfer method^30^, we patterned selected new fluorophore **5d** in the form of an array consisting of 10 × 10 spots (Fig. 5b), and the logo of Friedrich-Alexander University, consisting of single overlapping spots (Fig. 5c). Hereby, laser irradiation (wavelength of 532 nm) transferred the small amounts of 2,6-dicyanoaniline derivative **5d** from the donor slide to an acceptor (see Supplementary Fig. 2 and Supplementary Note 1). In accord with the absorption spectrum of compound **5d**, an excitation wavelength of 254 nm was chosen. The expected fluorescent image was detected with an optical set-up developed for rapid wide-field screening of chromophores (see Supplementary Fig. 2). The fact that the combinatorial laser-induced forward transfer procedure does not harm push–pull chromophore **5d** opens up the possibility to test and optimize optical properties in a high-density array format by performing parallel screenings for different chromophores at the same time. In addition, **5d** showed also solvatochromic properties (Fig. 5d).

### One-pot 10-step synthesis of fluorescent quinazolines

**Figure 6:**
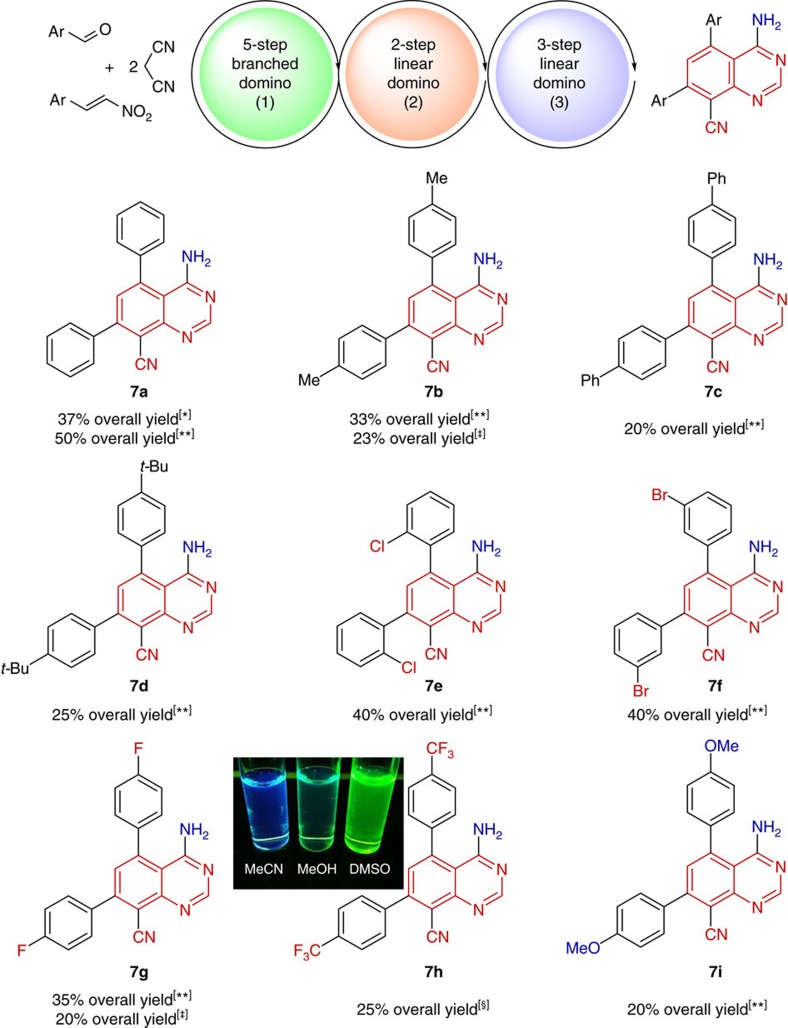
Synthesis and fluorescence properties of quinazolines. Synthesis of new quinazolines via 10-step one-pot process and demonstration of the fluorescence properties of **7h** in different solvents illuminated by ultraviolet light. * (1) Catalyst (DHQ)_2_PHAL (5 mol%), CH_2_Cl_2_, RT, 3 h, (2) AcOH/Py=1/1, 1 eq. NH_4_OAc, 80 °C, 15 h; (3) formamide, reflux, 2 h. ** (1) Catalyst (DHQ)_2_PHAL (20 mol%), CH_2_Cl_2_, 0 °C, 6 h, (2) AcOH, 130 °C, 15 h, (3) formamide, reflux, 2 h. ‡ (1) Catalyst (DHQ)_2_PHAL (5 mol%), CH_2_Cl_2_, RT, 3 h, (2) AcOH, 130 °C, 15 h, (3) formamide, reflux, 2 h. § (1) Pyrrolidine (20 mol%), CH_2_Cl_2_, 0 °C, 6 h, (2) AcOH, 130 °C, 15 h, (3) formamide, reflux, 2 h.

Consequently, anticipated one-pot synthesis of quinazolines **7a–i** was performed via combination of our new seven-step one-pot process with a three-step linear domino reaction using formamide as a suitable reagent in 2 h reaction time (Figs 3 and 6). Reaction of **5a** with formamide can proceed through an imine formation (step 1), intramolecular condensation (step 2) and tautomerization (step 3) linear domino process (Fig. 3). We explored the substrate scope of the resulting 10-step one-pot process (Fig. 6). All applied aromatic substrates (carrying either neutral, electron-withdrawing or electron-donating groups) afforded high overall yields of 20–50% over 10 steps. Remarkably, calculated average yields of every individual step in these multistep one-pot reactions, deduced from a total yield of up to 67% (seven-step, Fig. 5a) and up to 50% (10-step, Fig. 6) is 94% and 93%, respectively.

### Photophysical study of selected fluorescent quinazoline

On example of selected compound **7h** we demonstrate the solvatochromy of new quinazolines (Fig. 6). Inspired by this interesting property, we conducted a basic photophysical study of **7h** in acetonitrile (see Supplementary Fig. 3, Supplementary Discussion and Supplementary Methods). First insights into the excited-state properties came from steady-state fluorescence measurements. Quinazoline compound **7h** exhibits strong fluorescence between 370 and 600 nm, with a maximum at 439 nm. The fluorescence properties of potentially bioactive compounds, for example, antiviral, antimalarial and anticancer agents, might be very useful for an *in vitro* monitoring of compound-treated cultured cells by use of fluorescence microscopy.

### Synthesis of artesunic acid–quinazoline hybrids

**Figure 7:**
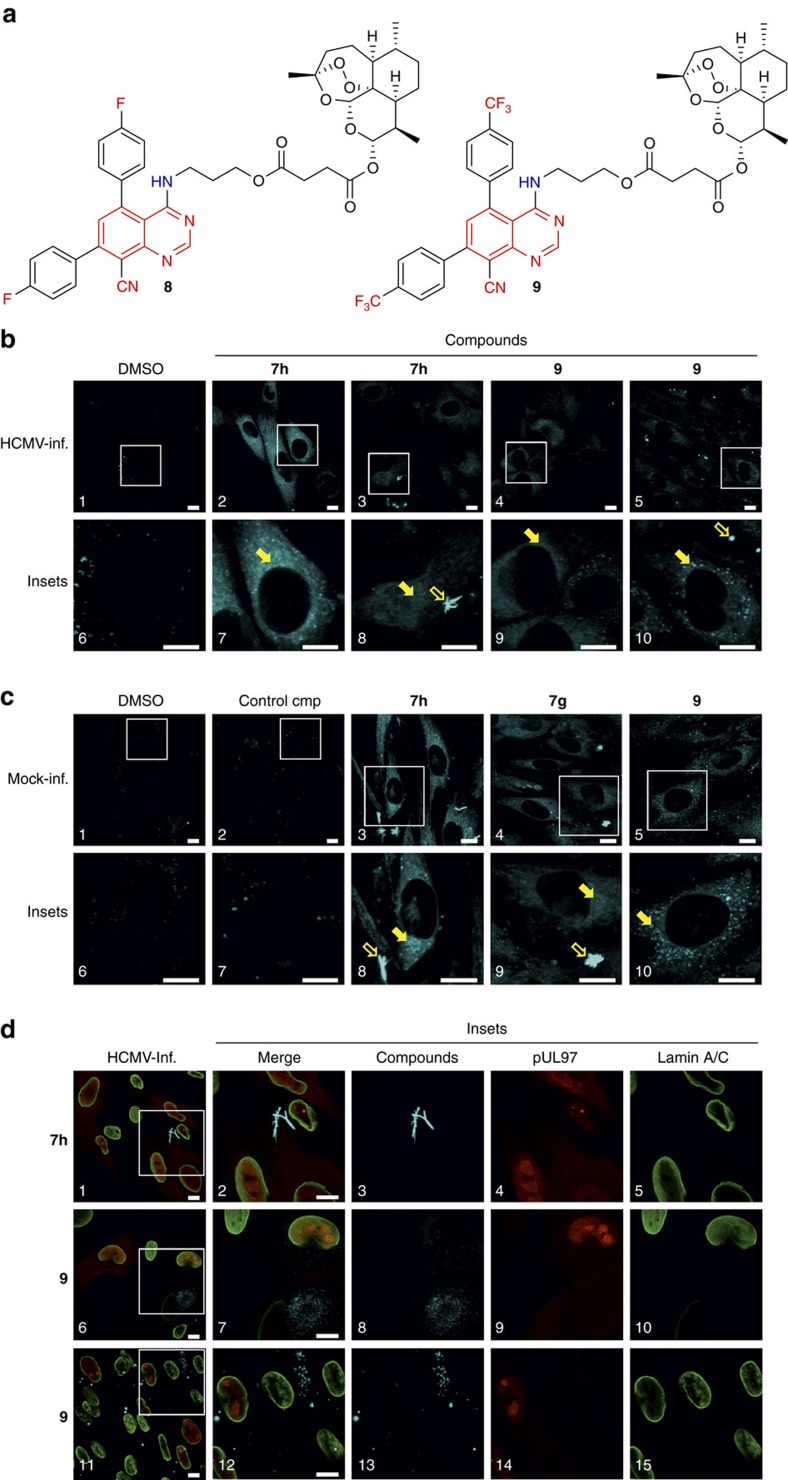
Microscopy-based visualization of cellular uptake of fluorescent quinazoline compounds. (**a**) Artesunic acid–quinazoline hybrids **8** and **9**. (**b**–**d**) Compounds **7h**, **7g** and **9** were analysed for properties of fluorescence imaging using confocal laser-scanning microscopy. HFFs were cultivated in six-well plates on cover slips and were either directly incubated with compounds by addition to the culture media for 20 h (**c**) or were added after HCMV infection (**b**,**d**; compound addition at 50 h post infection, fixation and analysis of cells 20 h later; scale bar, 10 μm). The chosen concentrations were 10 μM for compounds **7h**, **7g** and **9**, or 1 μM for compound **9** in panels 4, 9 (**b**), panels 5, 10 (**c**) and panels 6–10 (**d**), referring to the compounds' **EC**_**50**_ values of anti-HCMV activity (see Table 1). The depicted images are representative for three independent experiments. Lack of mycoplasm contamination was verified by routine 4,6-diamidino-2-phenylindole staining. General procedures of cell fixation, indirect immunofluorescence staining of proteins (**d**) and microscopic analysis have been described elsewhere^36^,^37^.

Human cytomegalovirus (HCMV) is a major human pathogen showing worldwide prevalence. Prevention and clinical interventions for HCMV disease are limited, since cross-resistance to all approved anti-HCMV drugs is increasingly observed^31^. As alternatives to standard drugs^23^,^32^, our ongoing search for highly active hybrid molecules^33^,^34^,^35^, which exceed their parent compounds in activity against HCMV, resulted in synthesis of new artesunic acid–quinazoline hybrids **8** and **9** (Fig. 7a and Supplementary Methods). We chose compounds **7g** and **7h** (Fig. 6) as coupling partners, since their fluorescence properties were considered beneficial for biological investigations. Artesunic acid on the other hand was selected because it proved to be a valuable building block in order to get highly bioactive compounds with just a few chemical transformations and also because of its promising pharmacological properties^23^,^32^,^35^.

### Antiviral activities of quinazolines and hybrid compounds

Subsequently, the selected quinazolines **7b**, **7g**, **7h**, **7i** and hybrids **8**, **9** were quantitatively analysed for their antiviral activity against HCMV (Table 1). The new quinazolines **7b**, **7g**, **7h** and **7i** consistently demonstrated a high activity against HCMV, among which the quinazoline heterocycle **7i** displayed the most potent antiviral efficacy with a lower *in vitro* **EC**_**50**_ value than the reference drug ganciclovir (Table 1).

Furthermore, our results clearly demonstrate the great potential of the hybridization concept. Artesunic acid–quinazoline hybrids **8** and **9** (**EC**_**50**_ 0.6±0.1 and 0.1±0.0 μM, respectively) are not only 6- to 46-fold more active than their parent compounds: artesunic acid, **7g** and **7h** (**EC**_**50**_ 3.8±0.4, 4.9±0.2 and 4.6±0.9 μM, respectively); they also exceed antiviral activity of the reference drug ganciclovir (**EC**_**50**_ 2.6±0.5 μM) that is in use for standard treatment of HCMV infections. The measurement of antiviral activity was based on automated fluorometry using a green fluorescent protein (GFP)-expressing, recombinant HCMV (excitation 485 nm, emission 535 nm; see Supplementary Note 2, Supplementary Data 1 and Supplementary Fig. 4 for dose–response curves).

In addition, **EC**_**50**_ values were confirmed using non-GFP-expressing HCMV (strain AD169) by a classical plaque formation assay on HFFs (*n*=4), providing data consistent with the GFP-based data shown in Table 1, that is, compound **7h**, 7.3±0.7 μM; compound **8**, 0.3±0.0 μM; compound **9**, 0.3±0.1 μM. Moreover, antiviral activity was clearly separated from putative cytotoxic effects because of the results obtained with a standard trypan blue exclusion assay. For all compounds, cytotoxicity for primary HFFs (**CC**_**50**_) was low or undetectable at concentrations up to 100 μM (that is, cytotoxicity values obtained at the highest test concentration of 100 μM remained under the cutoff level for all compounds except artesunic acid also comprising a high CC_50_ of 82.0±0.9), while anti-HCMV activities (**EC**_**50**_) were in a sub- or single-digit micromolar range. Consistent with these findings are results from a MTS-based cell proliferation assay, showing moderate or no antiproliferative effects on HFFs for all analysed compounds in concentrations up to 30 μM (see Supplementary Fig. 5 and Supplementary Methods). Thus, the new quinazolines and hybrid compounds can be regarded as selective, which is one of the most important aspects in drug design.

## Discussion

We describe the operationally simple and expeditious one-pot synthetic method—merging multistep domino reactions under fully metal-free conditions—providing unprecedented convenient access to a new class of antiviral 4,5,7,8-substituted quinazolines, bearing amino and cyano groups at the same time, in high overall yields, starting from simple and readily available compounds (Figs 3 and 6). Only one purification step is involved, leading to significant reduction of costs, waste and labour input. The investigated quinazolines **7b**, **7g**, **7h**, **7i** exhibit significant activity against HCMV. Furthermore, selected fluorescent quinazolines **7g**, **7h** were applied for synthesis of first artesunic acid–quinazoline hybrids **8** and **9**, which are, strikingly, also fluorescent and display superior anti-HCMV activity (**EC**_**50**_ down to 0.1±0.0 μM) compared to the parent compounds (artesunic acid and quinazolines) as well as the reference drug ganciclovir (**EC**_**50**_ 2.6±0.5 μM).

When analysing primary HFFs, the fluorescent compounds **7g**, **7h** and **9** could nicely be depicted both in extracellular (transparent arrows) and intracellular localizations (filled arrows; Fig. 7b,c). Interestingly, the extracellular signals included well-ordered crystalline structures (Fig. 7b, panel 8; Fig. 7c, panels 8–9) in the range of the applied concentrations (1–10 μM). The intracellular fluorescence pattern of the compounds showed a speckled accumulation mostly in cytoplasmic areas, thus visually indicating their efficient cellular uptake (Fig. 7b, panels 2–5 and 7–10; Fig. 7c, panels 3–5 and 8–10). A nonfluorescent artemisinin-derived control compound (Fig. 7c, panels 2 and 7; see also Supplementary Fig. 6), the DMSO panels (solvent controls) as well as the inspection of different excitation wavelengths of the confocal laser-scanning microscopy confirmed the specificity of signals and a lack of unwarranted cross-fluorescence. Of note, the fluorescent capacity of compounds **7h**, **7g** and **9** (depicted by microscopic imaging scored at a laser wavelength of 405 nm; Fig. 7b–d) did not interfere with GFP fluorometry, so that control measurements with these compounds did not produce signals above background levels. Importantly, the evaluation of virus-infected cells could also be included in this analytic approach, opening new perspectives in the investigation of antiviral activity and mechanistic details of the compounds (the latter point might be supported by the study of intracellular drug–target binding and localization in future applications). An indirect immunofluorescence counterstaining of viral and cellular proteins^36^,^37^ led to the detection of nuclear markers, that is, the viral protein kinase pUL97 and cellular lamins type A/C (Fig. 7d, panels 4, 9, 14 or 5, 10, 15, respectively), in the context of extracellular and intracellular fluorescence signals of compounds **7h** and **9** (Fig. 7d, panels 1–5 or 6–15, respectively). The direct co-staining of putative target proteins of the compounds seems to be a further realistic goal and might be realizable at a later stage.

The fact that the studied antiviral quinazoline compounds are fluorescent opens up a new vista of molecular imaging in drug development and mechanistic studies, avoiding the requirement of linkage of external fluorescent markers to quinazoline drugs. Through fluorescence these new compounds might then be analysed for details of putative biological activities, such as *in vitro* inhibitory properties against HCMV as demonstrated in the present study and also for the aspects of drug delivery in single cells and whole tissue.

In general, these unprecedented findings have important future implications for the development of efficient drugs (for example, antiviral, anticancer and antimalarial) for clinics based on fluorescent bioactive heterocycles as lead compounds.

## Methods

### General methods

For details of the reaction optimization procedures and product characterizations, see Supplementary Fig. 1, Supplementary Table 1 and Supplementary Methods. For X-ray data of compounds **4a**, **4a′** and **7a**, see Supplementary Tables 2–4. For fluorescent imaging with **5d**, see Supplementary Fig. 2 and Supplementary Note 1. For photophysical studies of compound **7h**, see Supplementary Fig. 3, Supplementary Methods and Supplementary Discussion. For antiviral studies of selected compounds in this manuscript, see Supplementary Figs 4 and 5, Supplementary Methods and Supplementary Note 2. For ^1^H, ^13^C NMR spectra of the compounds in this article, see Supplementary Figs 6–52.

### Data availability

CCDC 1472467 (**4a**), 1472469 (**4a′**) and 1472470 (**7a**) contain the supplementary crystallographic data for this paper. These data are provided free of charge by The Cambridge Crystallographic Data Centre. The authors declare that all other data supporting the findings of this study are available within the article and its Supplementary Information file.

## Additional information

**How to cite this article:** Held, F. E. *et al*. Facile access to potent antiviral quinazoline heterocycles with fluorescence properties via merging metal-free domino reactions. *Nat. Commun.* **8,** 15071 doi: 10.1038/ncomms15071 (2017).

**Publisher's note**: Springer Nature remains neutral with regard to jurisdictional claims in published maps and institutional affiliations.

## Supplementary Material

Supplementary Information Supplementary figures, supplementary tables, supplementary notes, supplementary discussion, supplementary methods and supplementary references.

Supplementary Data 1 Excel sheet with EC50 data.

## Figures and Tables

**Figure 1:**
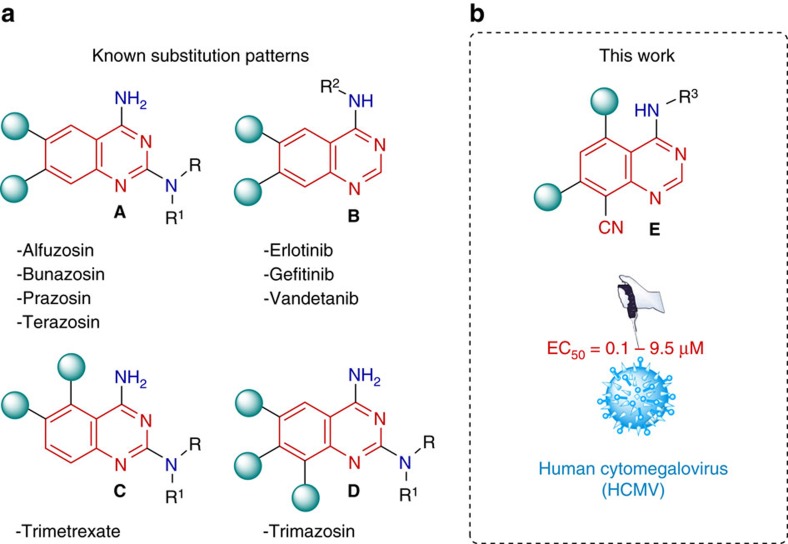
Quinazoline scaffolds with different substitution patterns. (**a**) List of the selected corresponding non-fluorescent quinazoline-marketed drugs: Alfuzosin (adrenergic blocker), Bunazosin (antihypertensive agent), Prazosin (adrenergic blocker), Terazosin (adrenergic blocker), Erlotinib (anticancer agent), Gefitinib (anticancer agent), Vandetanib (tyrosine kinase inhibitor), Trimetrexane (dihydrofolate reductase inhibitor) and Trimazosin (antihypertensive agent). (**b**) Antiviral quinazolines with fluorescence properties developed in this work.

**Table 1:** EC_50_ values of anti-HCMV activity (AD169-GFP) determined in primary HFFs.

**Compound**	**HCMV EC**_**50**_ (μM)*†	**HFFs CC**_**50**_ (μM)*‡
Ganciclovir	2.6±0.5	>100
Artesunic acid	3.8±0.4	82.0±0.9
Artemisinin	>10	>100
**7b**	9.5±4.8	>100
**7g**	4.9±0.2	>100
**7h**	4.6±0.9	>100
**7i**	0.6±0.1	>100
**8**	0.6±0.1	>100
**9**	0.1±0.0	>100

^*^The cell culture-based systems for the determination of **EC**_**50**_ and **CC**_**50**_ values (trypan blue exclusion assay) has been previously reported^38^.

^†^*n*=4.

^‡^*n*=6 (except artesunic acid: *n*=3).
