# Supplementary material for: Facile access to potent antiviral quinazoline heterocycles with fluorescence properties via merging metal-free domino reactions
Source: Nat Commun. 2017 May 2;8:15071. doi: 10.1038/ncomms15071 (PMC5418574; doi:10.1038/ncomms15071)
Supplement: Supplementary Information — Supplementary figures, supplementary tables, supplementary notes, supplementary discussion, supplementary methods and supplementary references. [file ncomms15071-s1.pdf]

Supplementary Figures

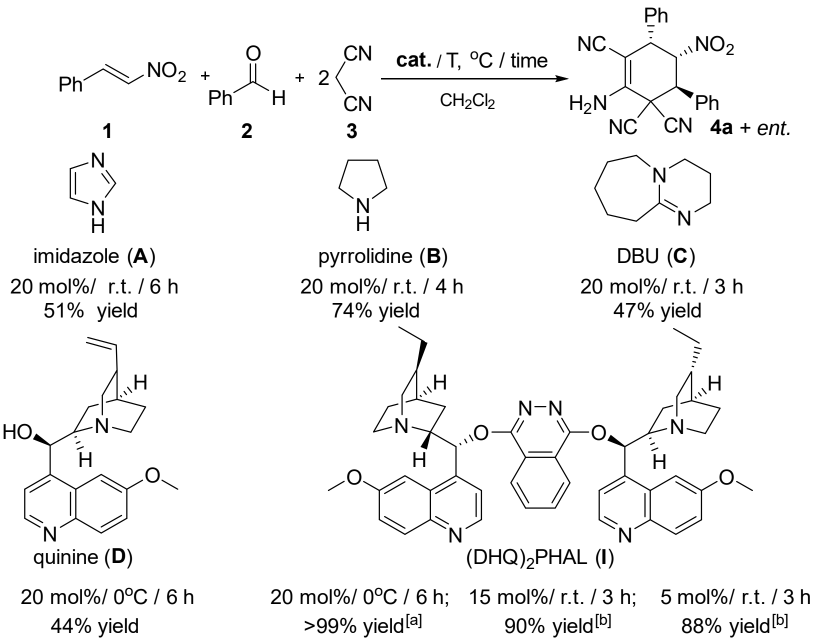

[a] Yield of isolated product. [b] Yield was determined by ^{1}H NMR.

Supplementary Figure 1. Catalyst screening for five-step branched domino reaction (see also Supplementary Methods).

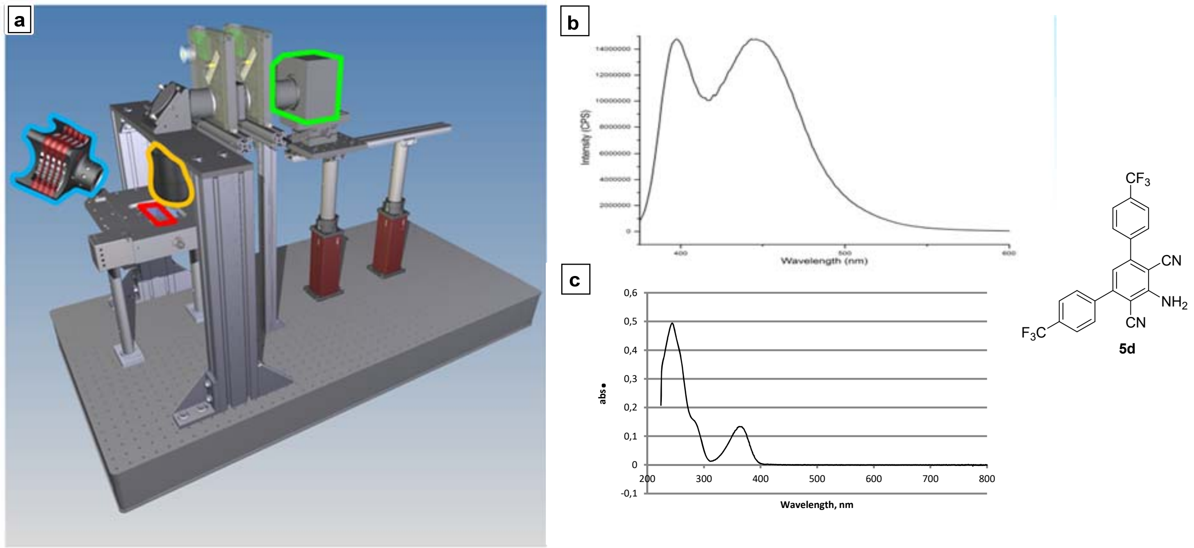

Supplementary Figure 2. (a) Scheme of the optical setup for fluorescent imaging. A UV-Lamp (254 nm) excites the chromophores on the slide (red). The emitting fluorescent signal is collected by an objective (orange) and detected by a CMOS camera (green). To exclude the excitation light from the fluorescent light, a long pass filter (cut-off wavelength 475 nm) is mounted in advance to the camera. The system allows to image 56 mm^2 with a resolution of 1.25 µm per pixel. **(b)** Emission and **(c)** absorption spectrum of compound **5d** (see also Supplementary Note 1).

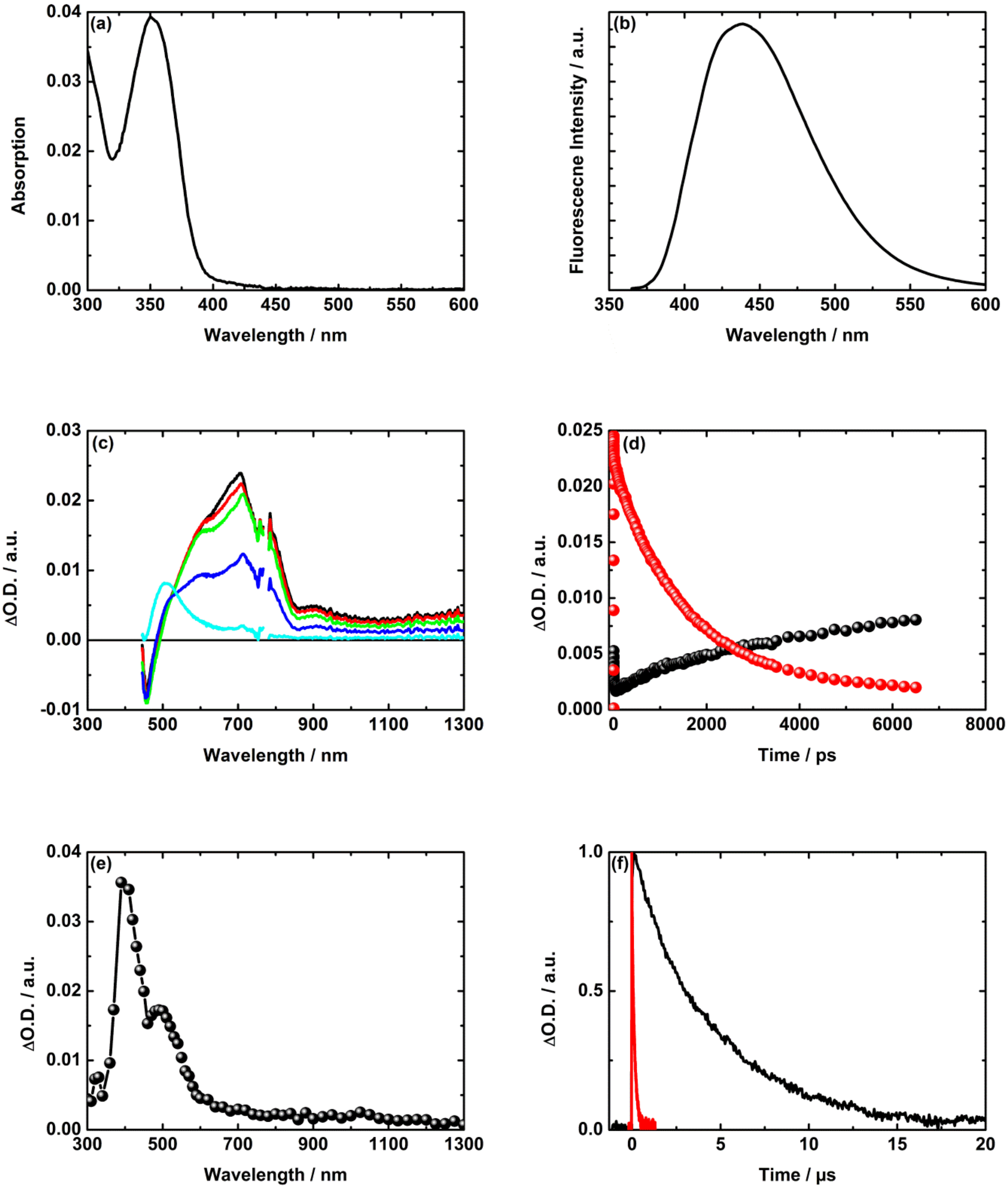

Supplementary Figure 3. (a) Absorption spectrum of **7h** in acetonitrile. (**b**) Fluorescence spectrum of **7h** in acetonitrile upon photoexcitation at 350 nm. (**c**) Femtosecond transient absorption spectra of **7h** in argon-saturated acetonitrile; 1 ps (black), 10 ps (red), 100 ps (green), 1000 ps (blue), and 6500 ps (cyan) after laser excitation at 258 nm. (**d**) Corresponding absorption time profiles at 510 nm (black) and 710 nm (red). (**e**) Nanosecond transient absorption spectrum of **7h** in argon-saturated acetonitrile; 1 μs after laser excitation at 355 nm. (**f**) Corresponding normalized absorption time profiles at 390 nm measured under argon saturation (black) and oxygen saturation (red) (see also Supplementary Discussion and Supplementary Methods).

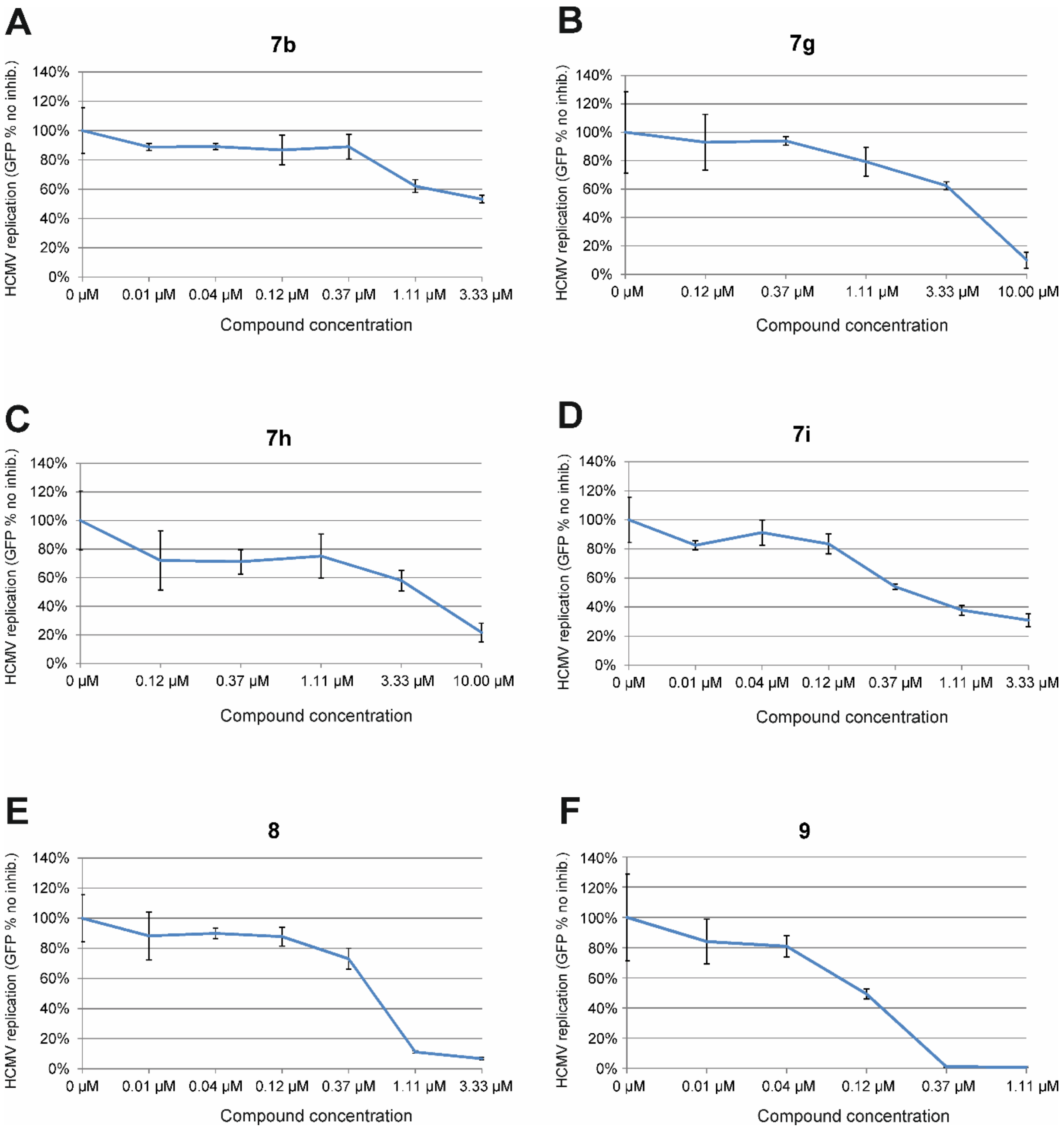

Supplementary Figure 4. Dose response curves of anti-HCMV activity (see also Supplementary Note 2). Compounds were analyzed in the HCMV GFP based replication assay; data presented in the individual curves refer to EC_{50} values in Table 1 of the main text.

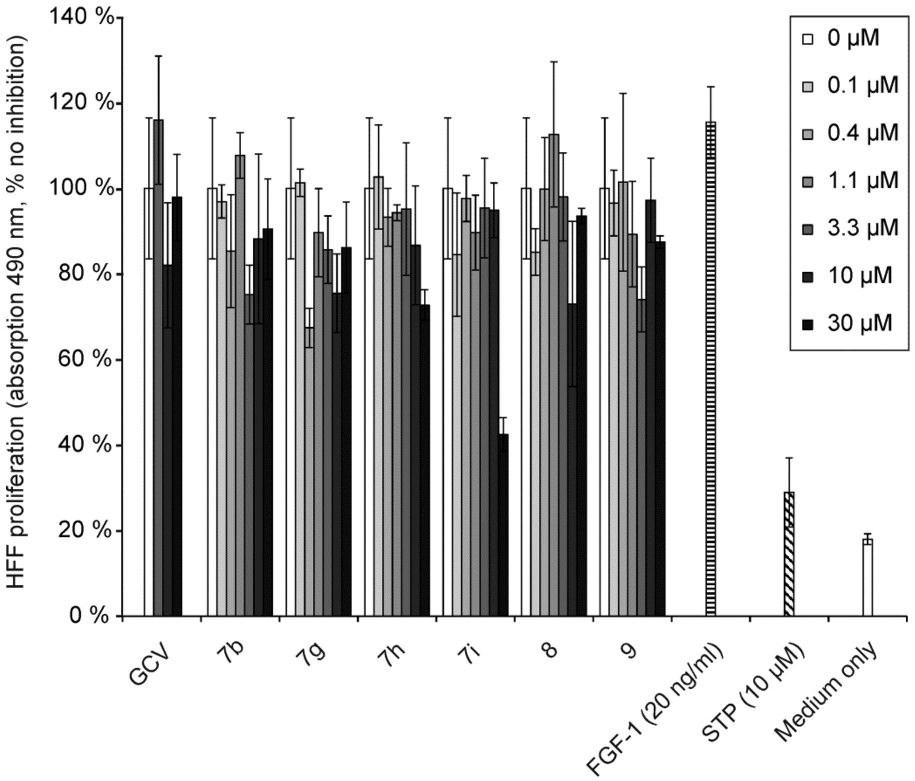

Supplementary Figure 5. Cell proliferation in the presence of quinazoline heterocycles. Proliferating layers of primary human fibroblasts (4500 cells seeded per well of 96-well plates) were incubated with ganciclovir (GCV), quinazoline heterocycles, fibroblast growth factor 1 (FGF-1) or staurosporine (STP) at the concentrations indicated. The release of NADPH/NADH from proliferating cells was measured by the use of the CellTiter 96® Aqueous One Solution Cell Proliferation Assay (Promega; n = 3, mean ± SD) (see also Supplementary Methods).

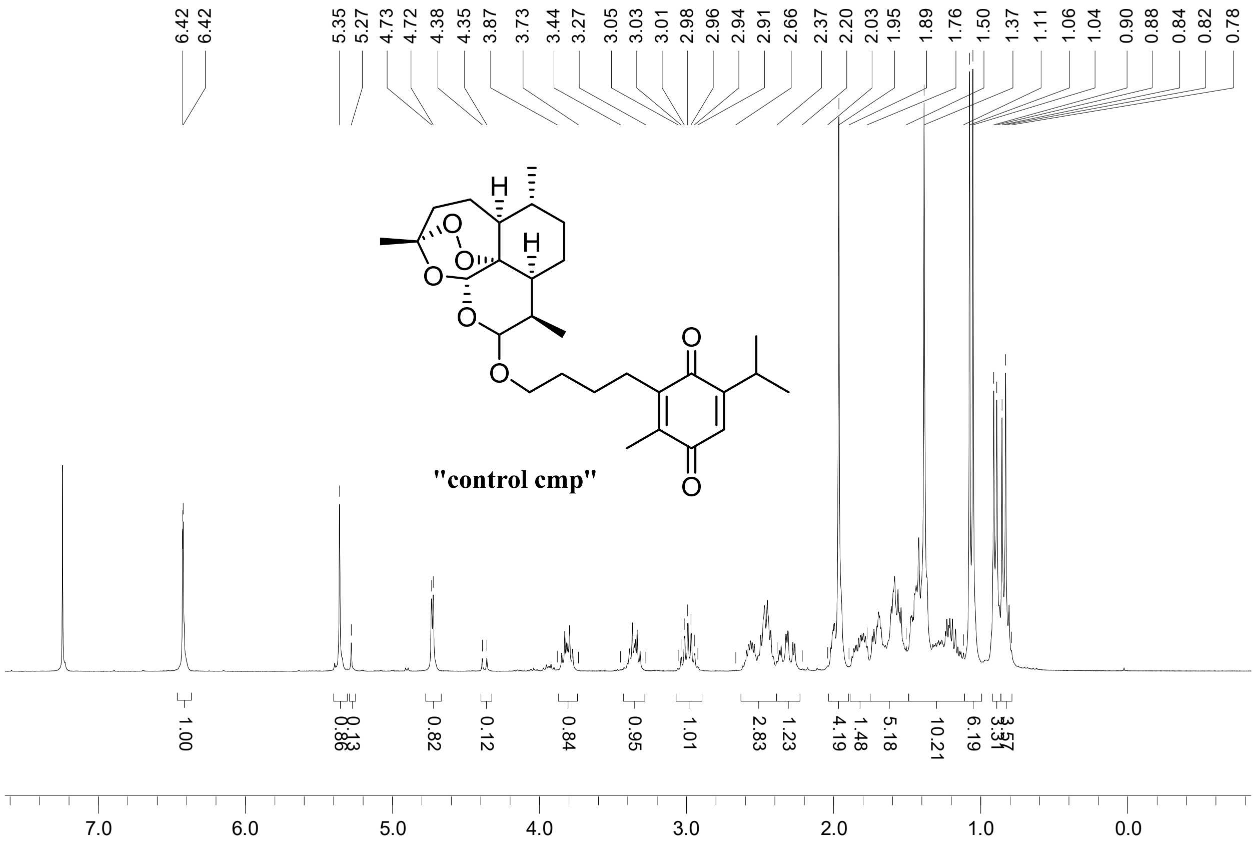

Supplementary Figure 6. Structure and ^{1}H-NMR spectrum (300 MHz, $CDCl_3$) of the nonfluorecent artemisinin-thymoquinone hybrid[1] used as a control compound (see Figure 7c of the paper).

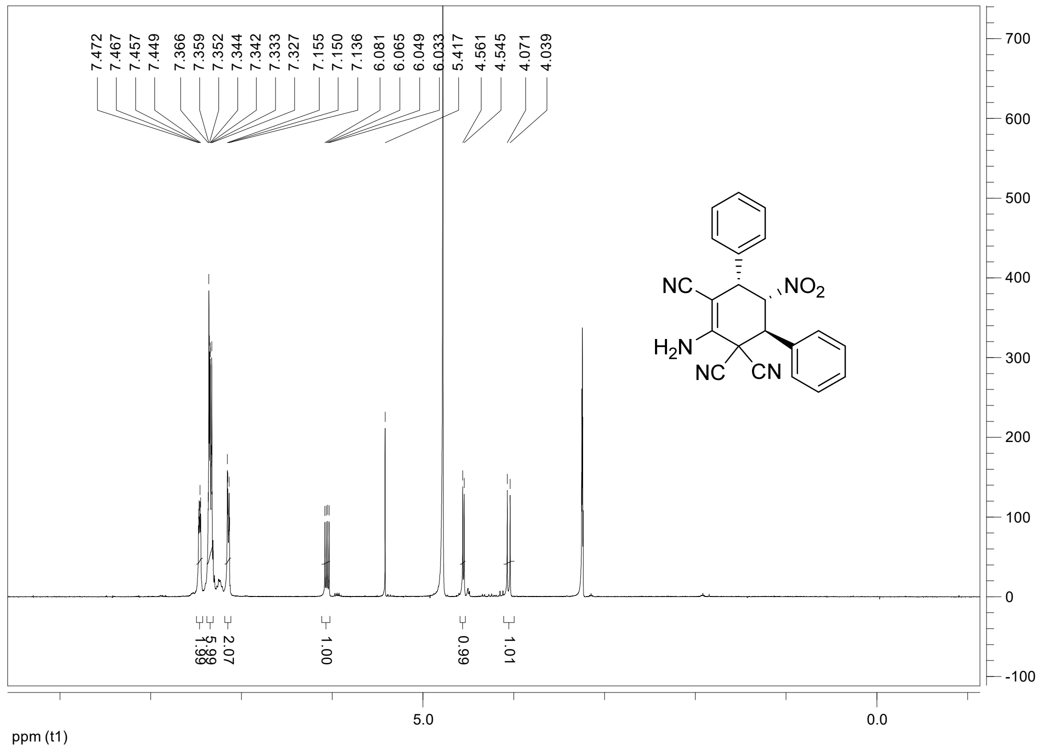

Supplementary Figure 7. Compound 4a: ^{1}H-NMR (300 MHz, $CDCl_3$)

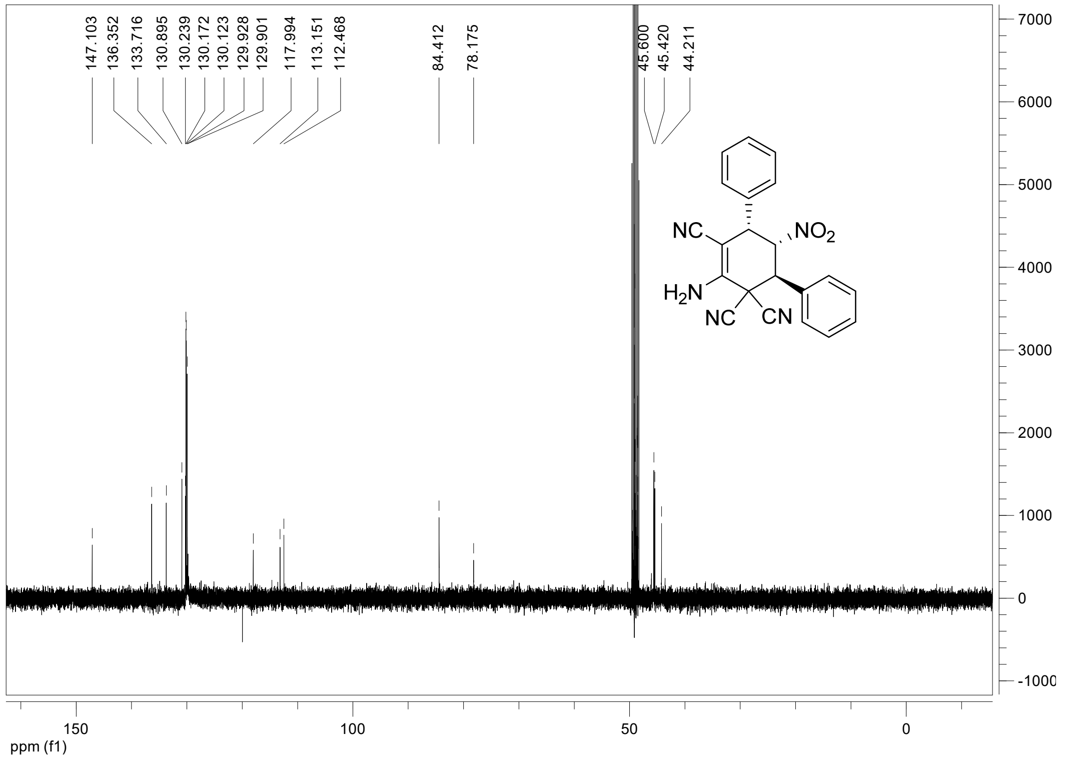

Supplementary Figure 8. Compound 4a: ^{13}C-NMR (100 MHz, $CDCl_3$)

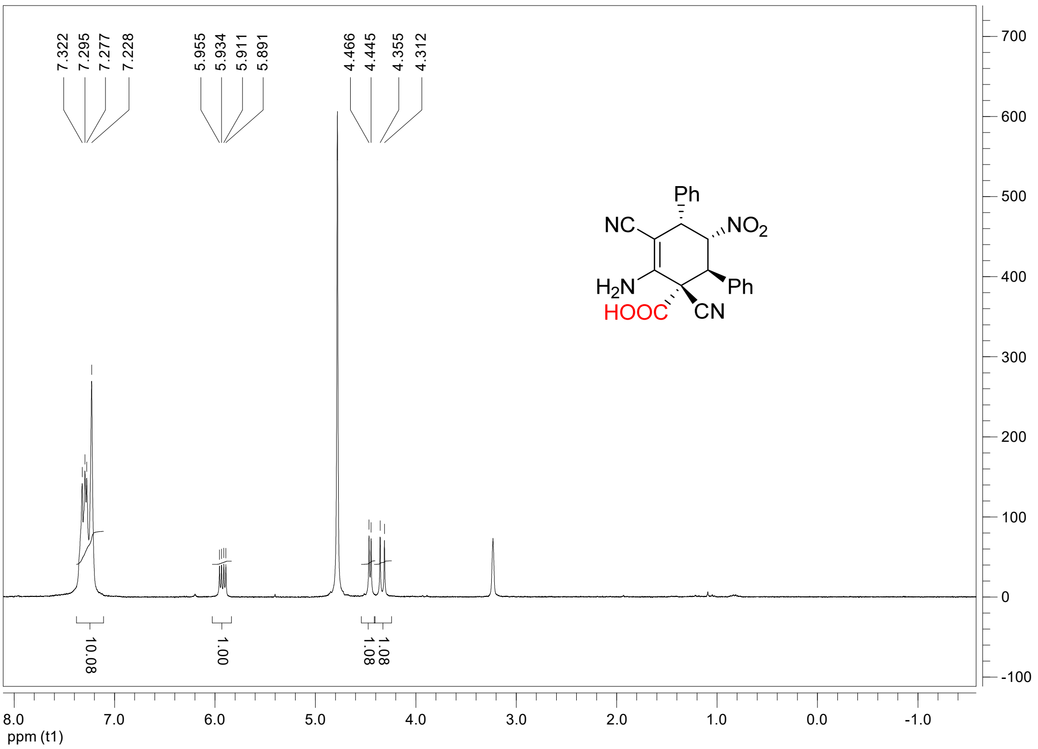

Supplementary Figure 9. Compound 4a': ^{1}H-NMR (300 MHz, MeOD)

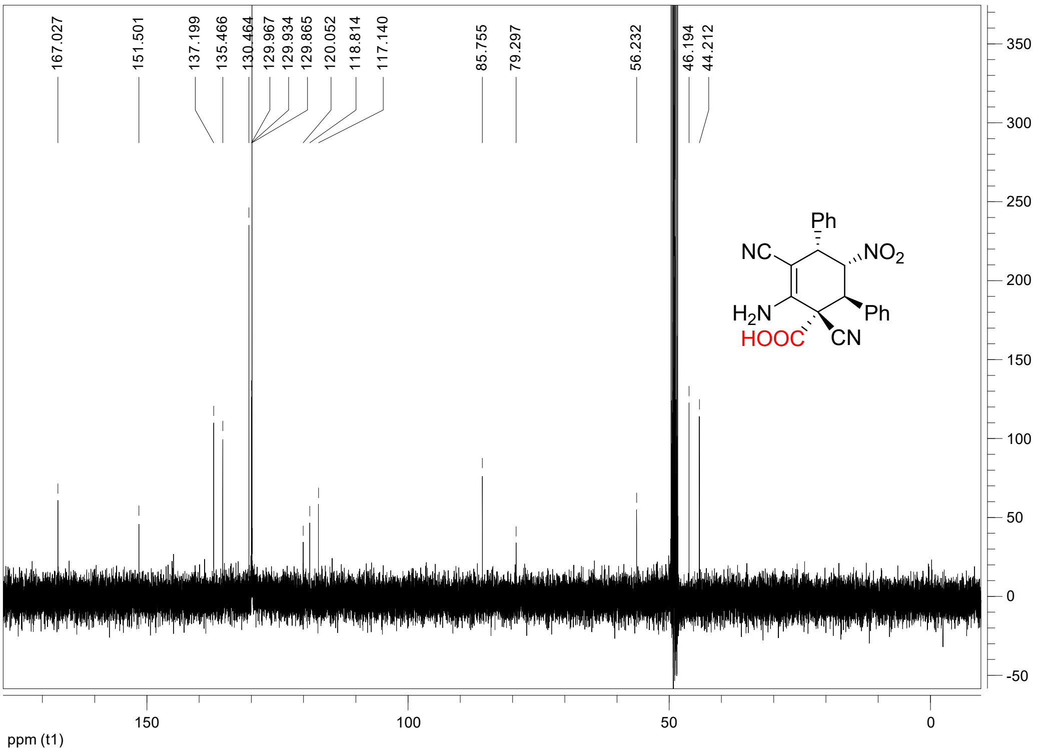

Supplementary Figure 10. Compound 4a': ^{13}C-NMR (100 MHz, MeOD)

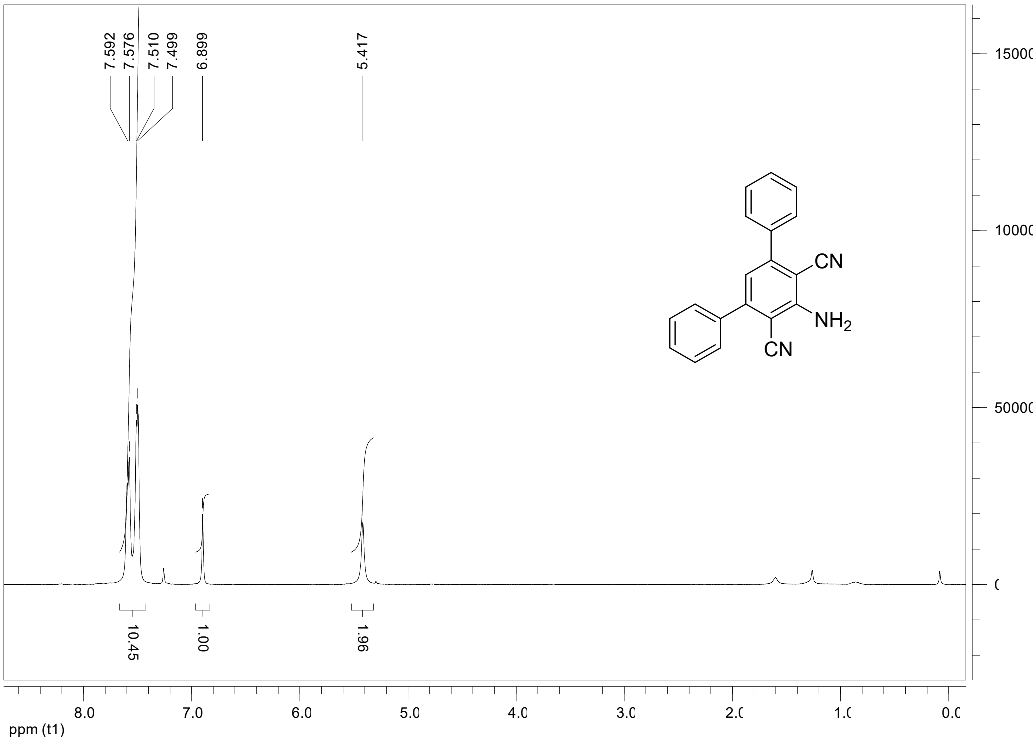

Supplementary Figure 11. Compound 5a: ^{1}H-NMR (400 MHz, $CDCl_3$)

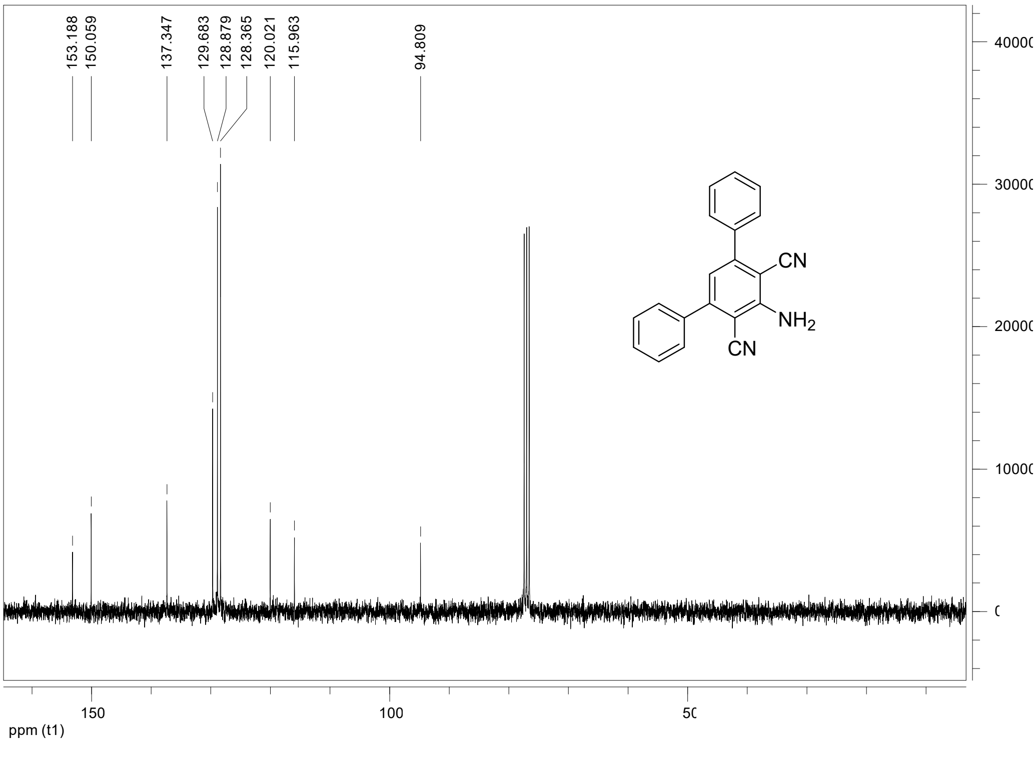

Supplementary Figure 12. Compound 5a: ^{13}C-NMR (100 MHz, $CDCl_3$)

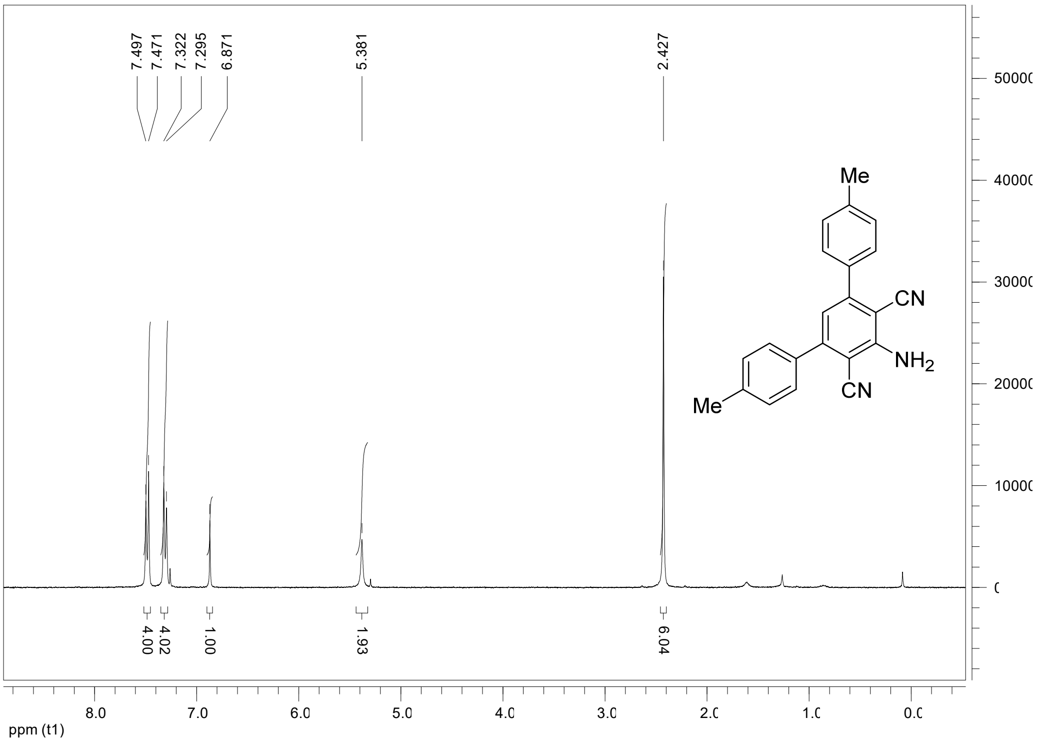

Supplementary Figure 13. Compound 5b: ^{1}H-NMR (300 MHz, $CDCl_3$)

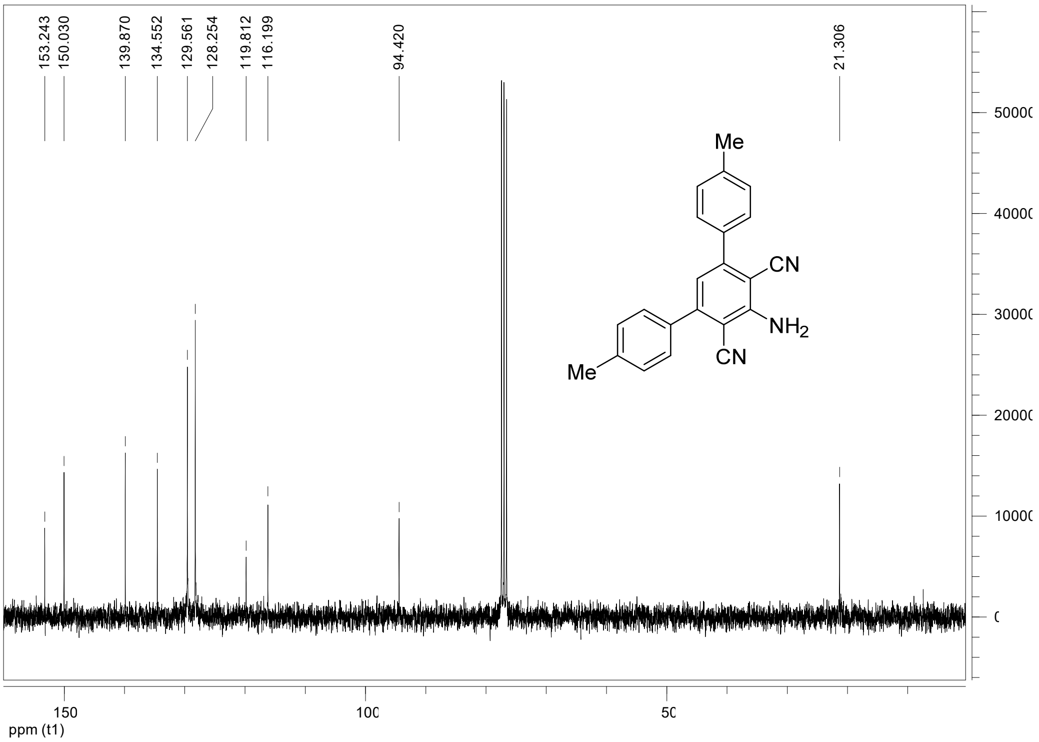

Supplementary Figure 14. Compound 5b: ^{13}C-NMR (100 MHz, $CDCl_3$)

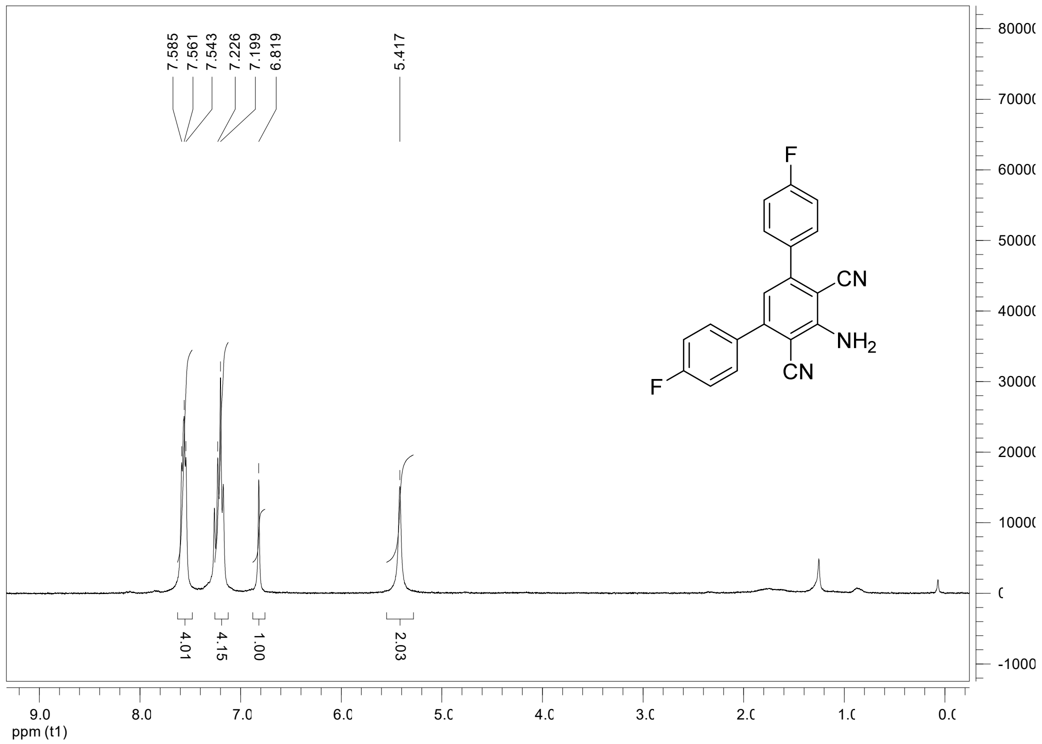

Supplementary Figure 15. Compound 5c: ^{1}H-NMR (300 MHz, $CDCl_3$)

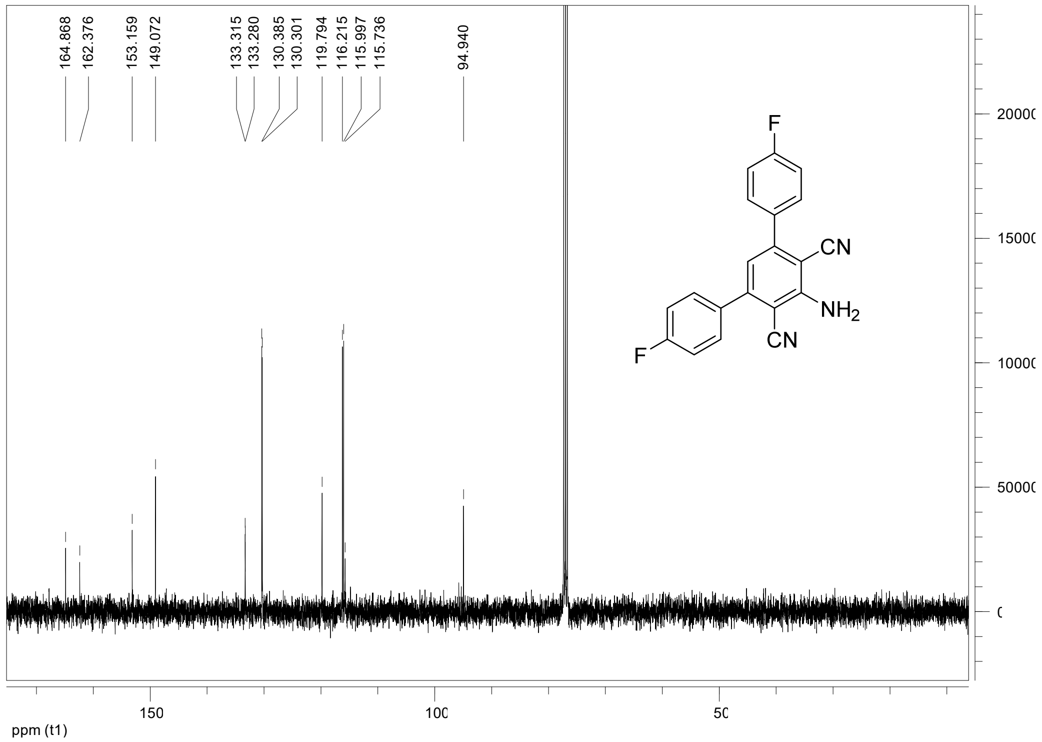

Supplementary Figure 16. Compound 5c: ^{13}C-NMR (100 MHz, $CDCl_3$)

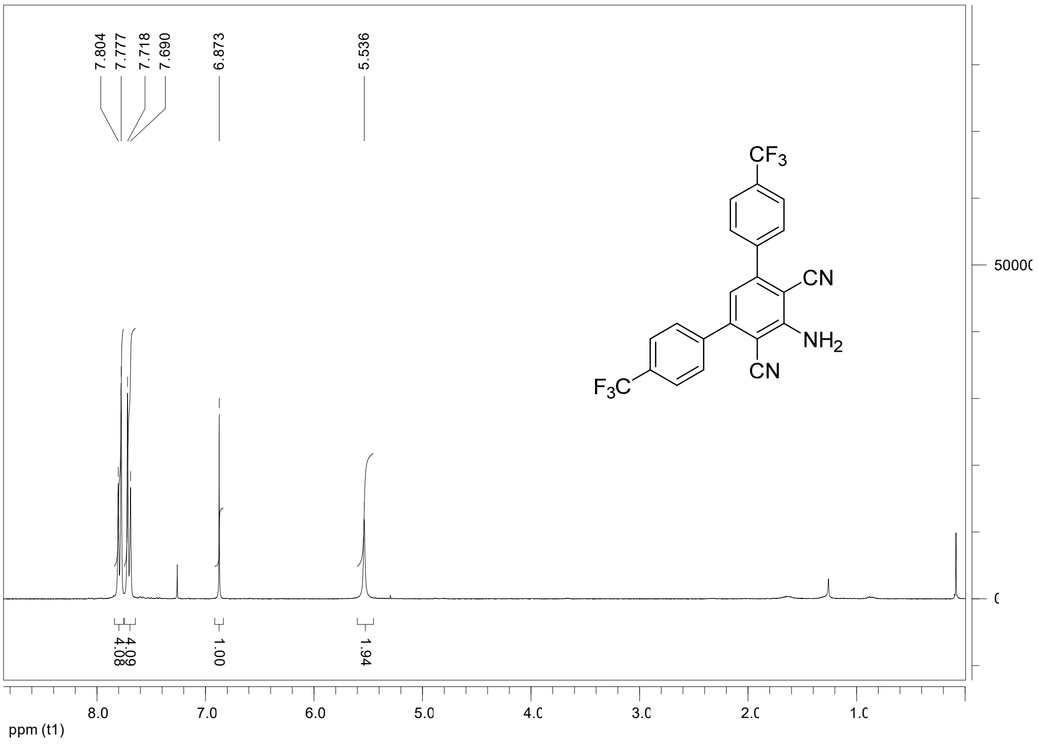

Supplementary Figure 17. Compound 5d: ^{1}H-NMR (300 MHz, $CDCl_3$)

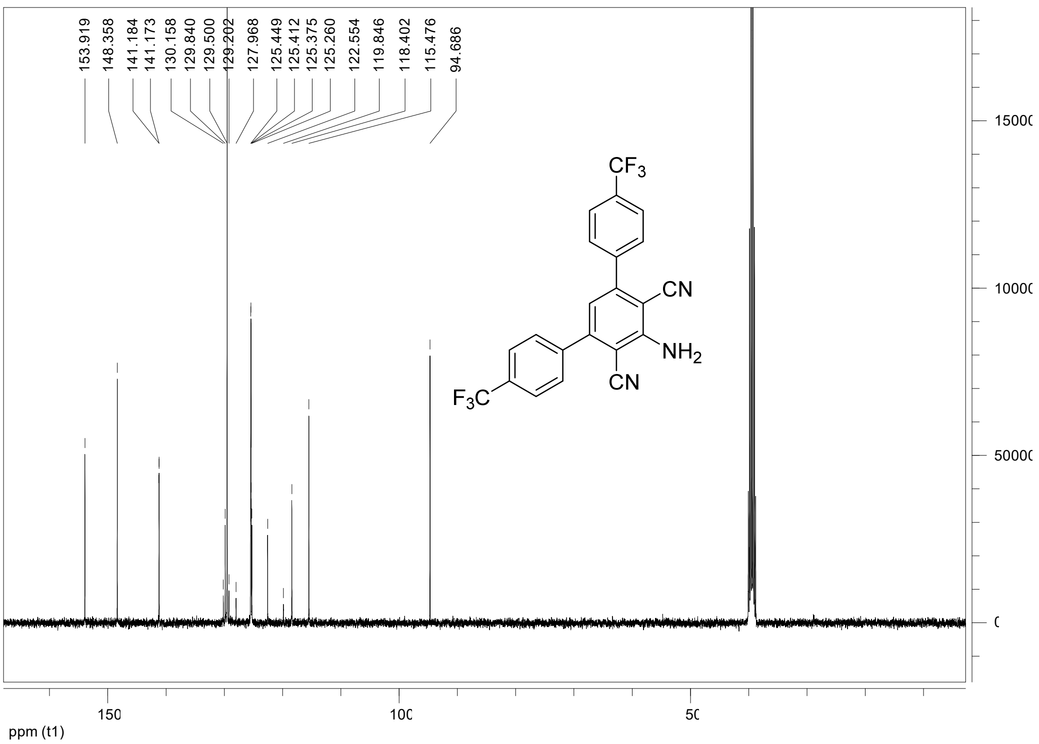

Supplementary Figure 18. Compound 5d: ^{13}C-NMR (100 MHz, $DMSO_{d6}$)

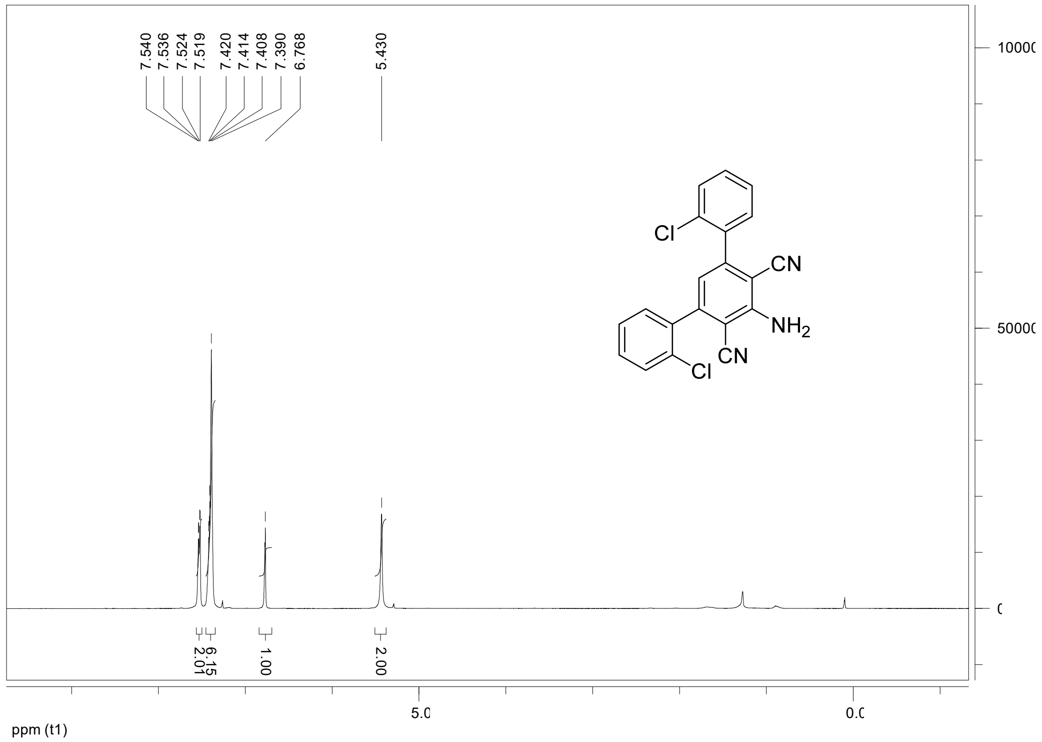

Supplementary Figure 19. Compound 5e: ^{1}H-NMR (400 MHz, $CDCl_3$)

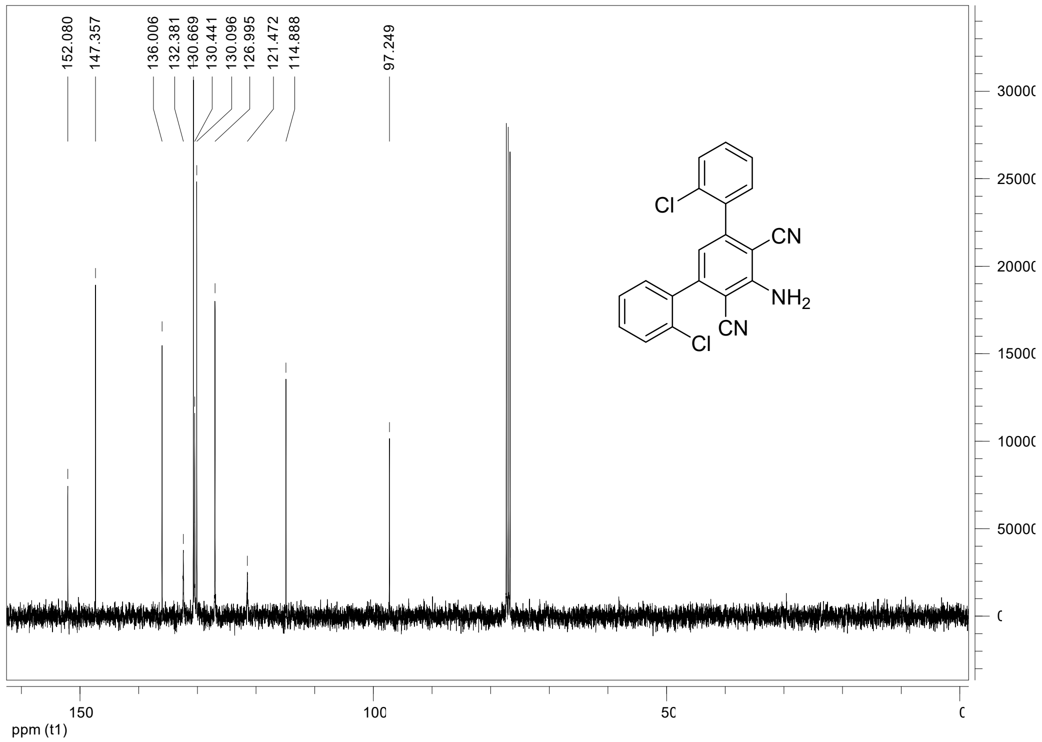

Supplementary Figure 20. Compound 5e: ^{13}C-NMR (100 MHz, $DMSO_{d6}$)

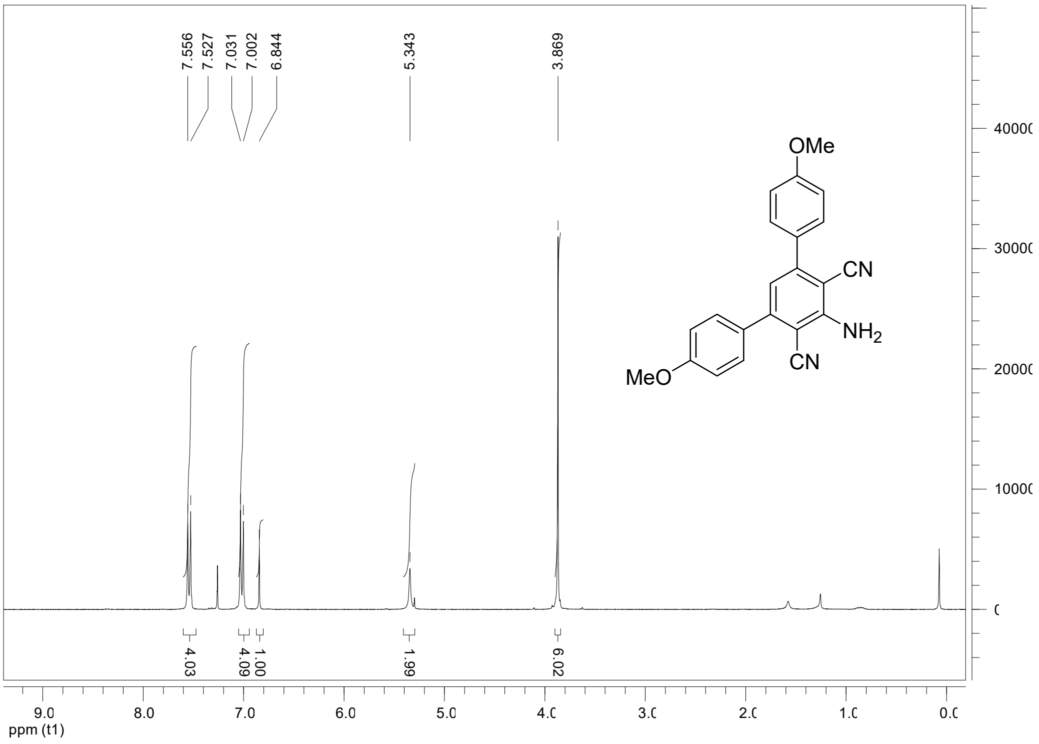

Supplementary Figure 21. Compound 5f: ^{1}H-NMR (300 MHz, $CDCl_3$)

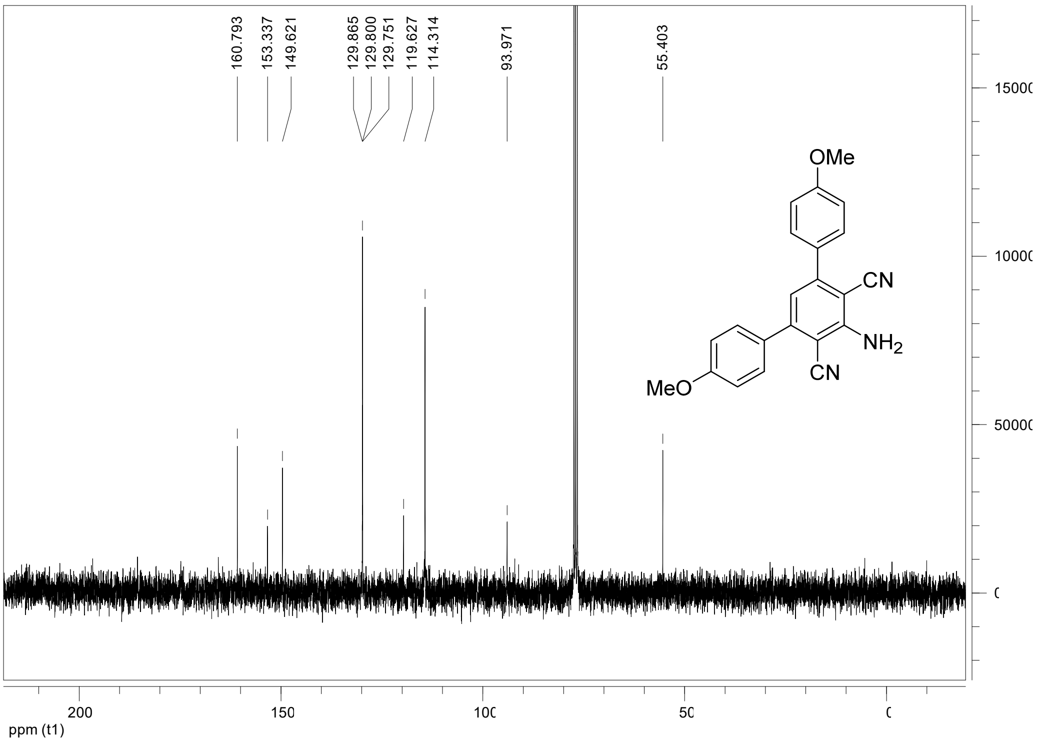

Supplementary Figure 22. Compound 5f: ^{13}C-NMR (100 MHz, CDCl3)

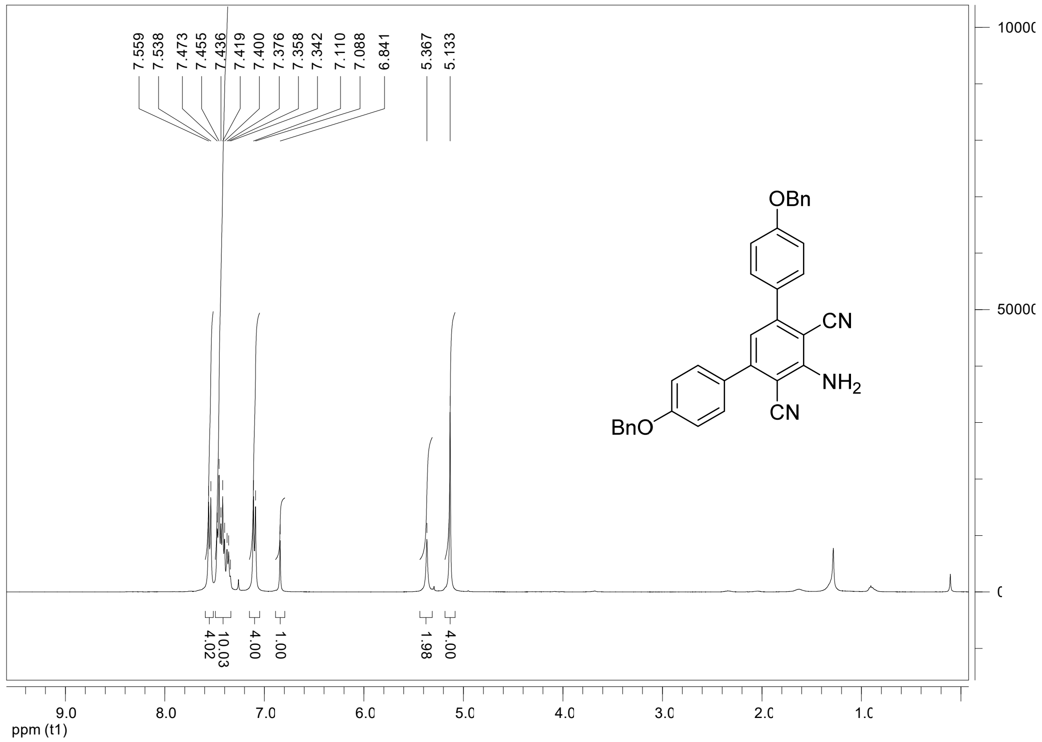

Supplementary Figure 23. Compound 5g: ^{1}H-NMR (400 MHz, $CDCl_3$)

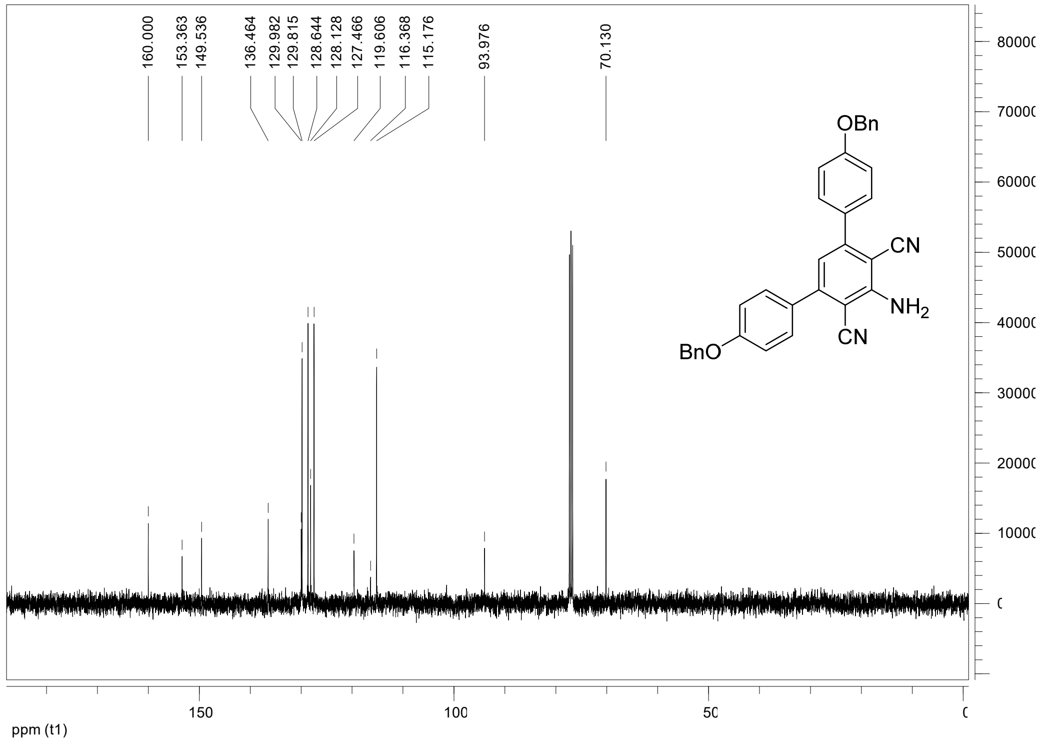

Supplementary Figure 24. Compound 5g: ^{13}C-NMR (100 MHz, $CDCl_3$)

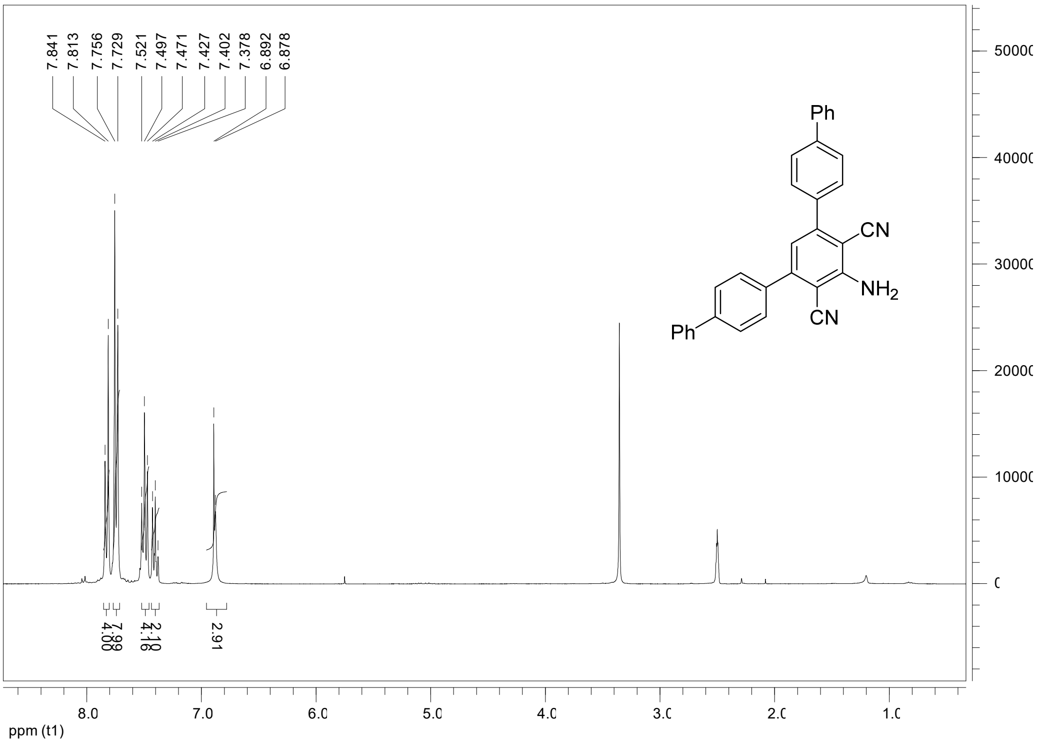

Supplementary Figure 25. Compound 5h: ^{1}H-NMR (300 MHz, $DMSO_{d6}$)

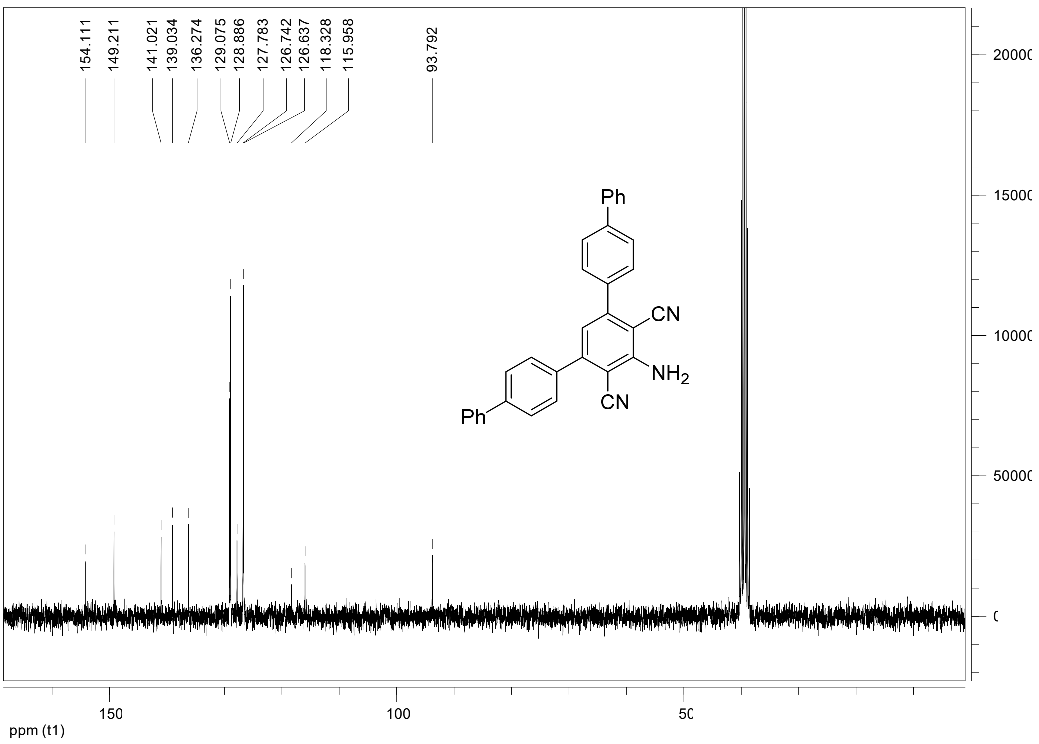

Supplementary Figure 26. Compound 5h: ^{13}C-NMR (100 MHz, $DMSO_{d6}$)

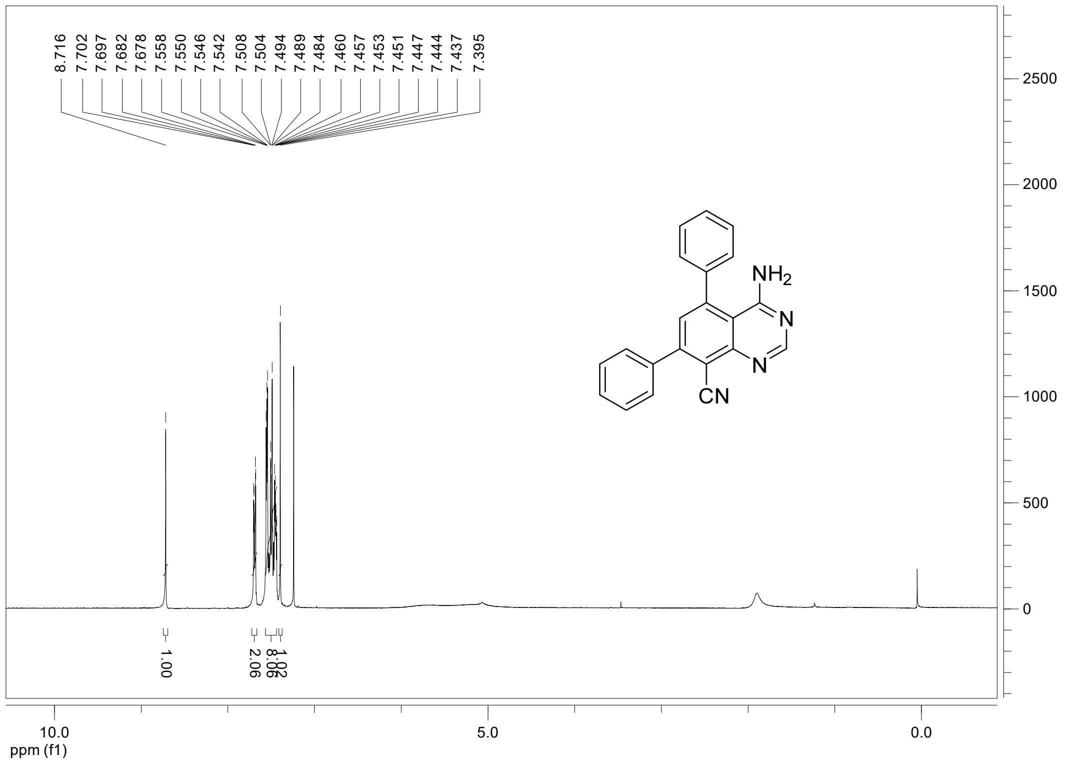

Supplementary Figure 27. Compound 7a: ^{1}H-NMR (400 MHz, $CDCl_3$)

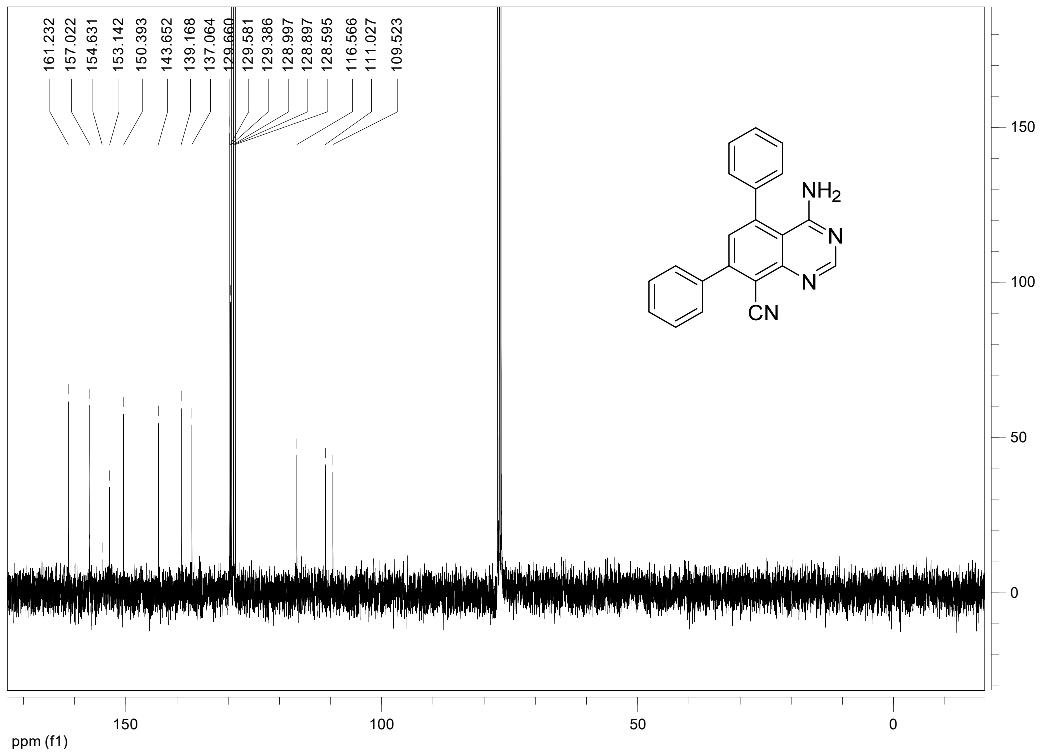

Supplementary Figure 28. Compound 7a: ^{13}C-NMR (100 MHz, $CDCl_3$)

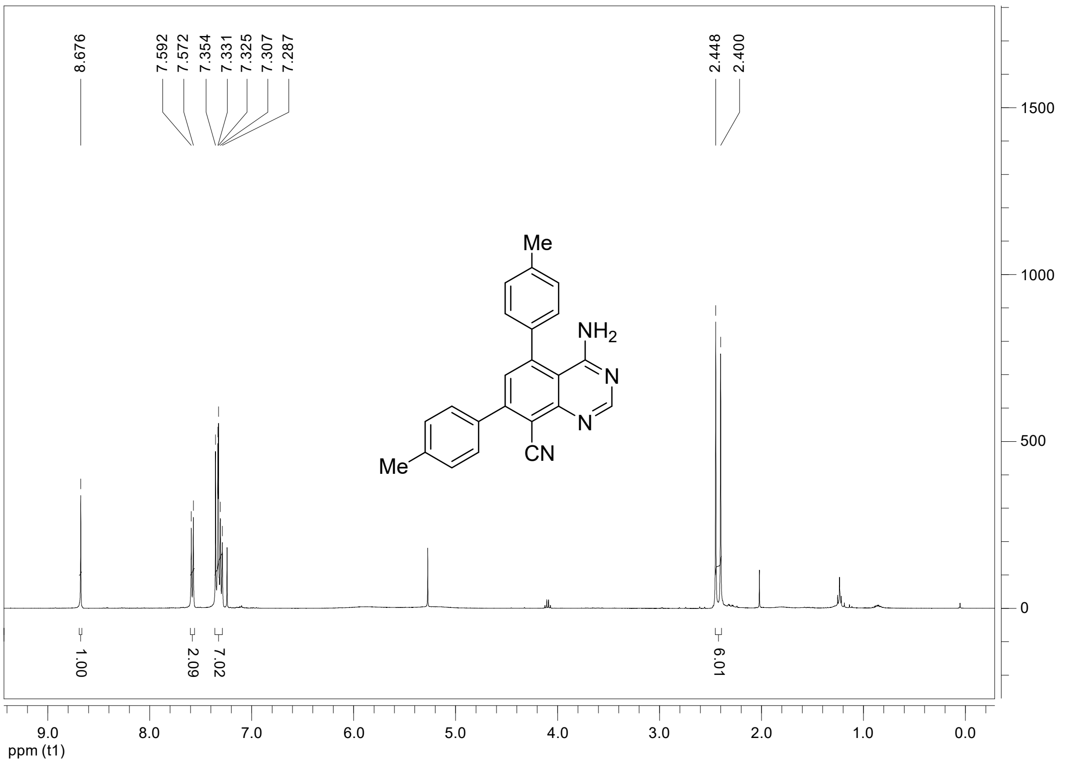

Supplementary Figure 29. Compound 7b: ^{1}H-NMR (400 MHz, $CDCl_3$)

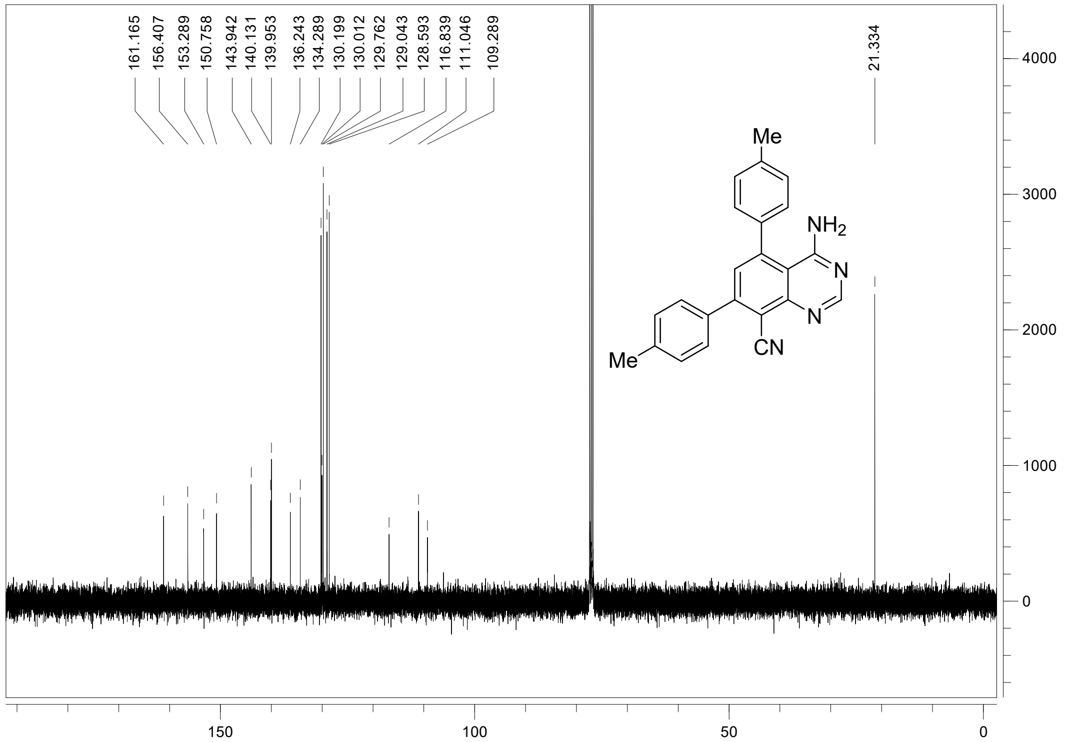

Supplementary Figure 30. Compound 7b: ^{13}C-NMR (100 MHz, $CDCl_3$)

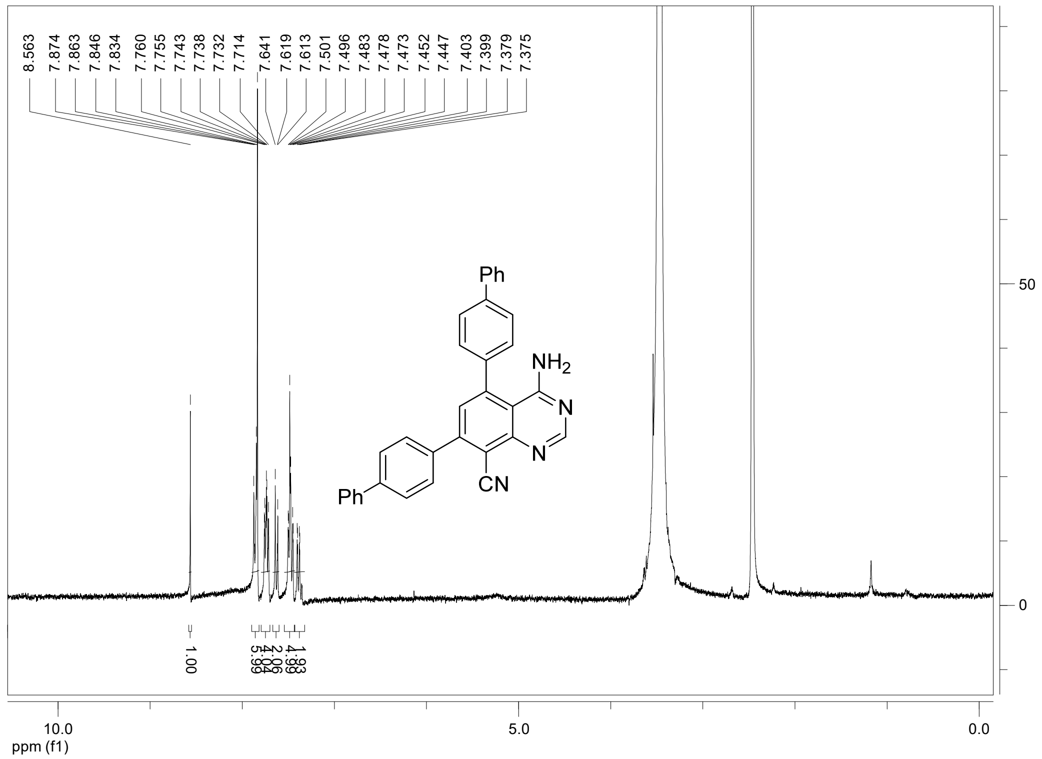

Supplementary Figure 31. Compound 7c: ^{1}H-NMR (400 MHz, DMSO$_{d6}$)

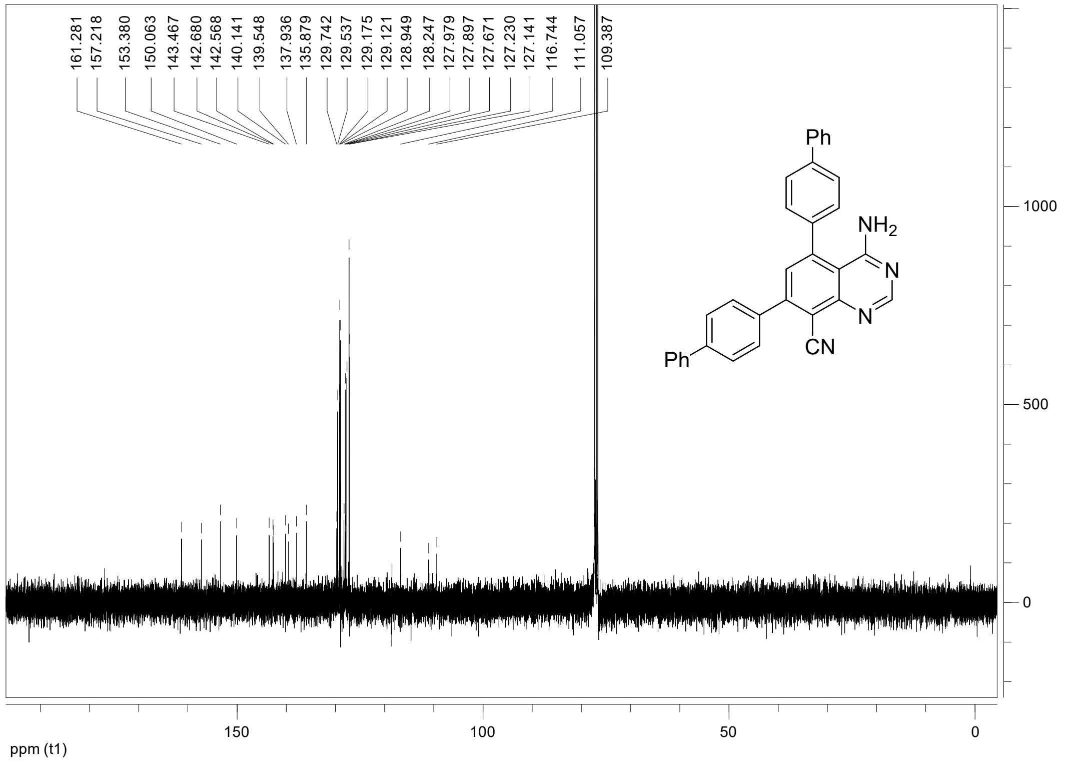

Supplementary Figure 32. Compound 7c: ^{13}C-NMR (100 MHz, DMSO$_{d6}$)

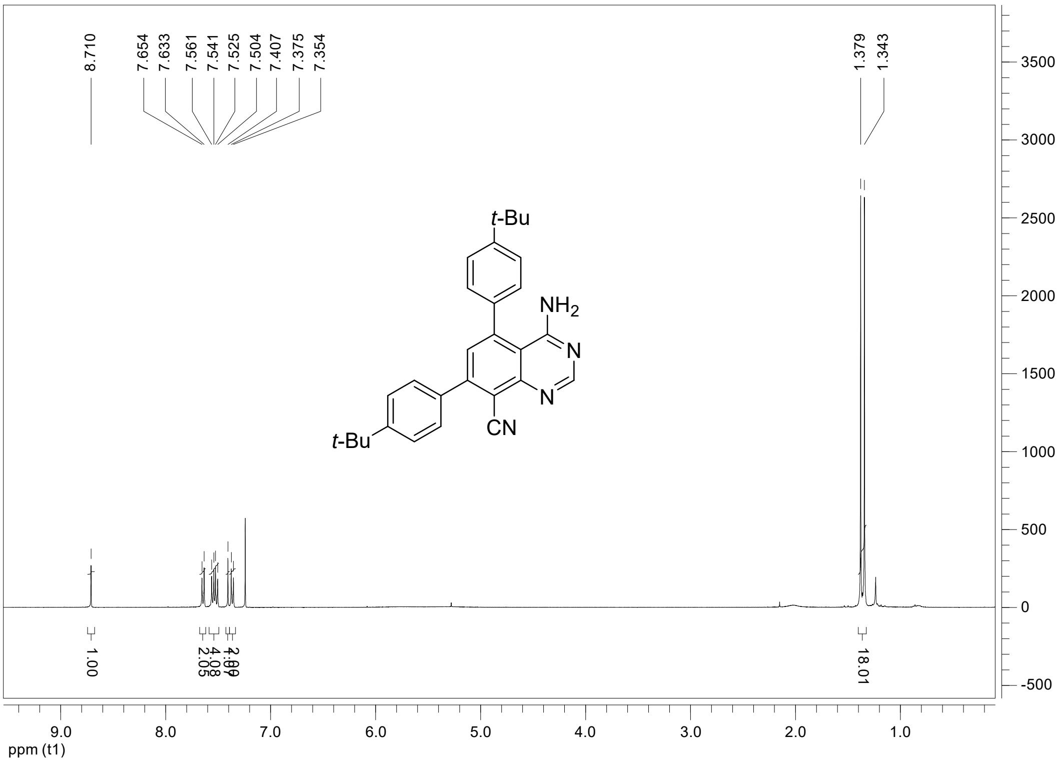

Supplementary Figure 33. Compound 7d: ^{1}H-NMR (400 MHz, $CDCl_3$)

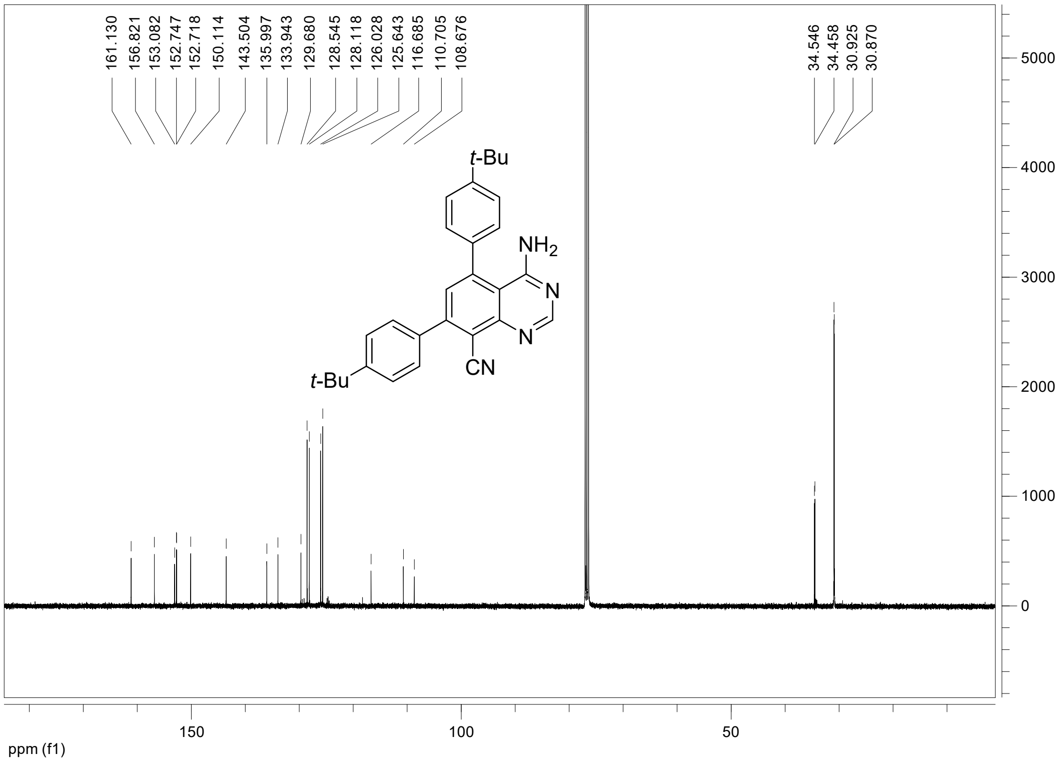

Supplementary Figure 34. Compound 7d: ^{13}C-NMR (100 MHz, $CDCl_3$)

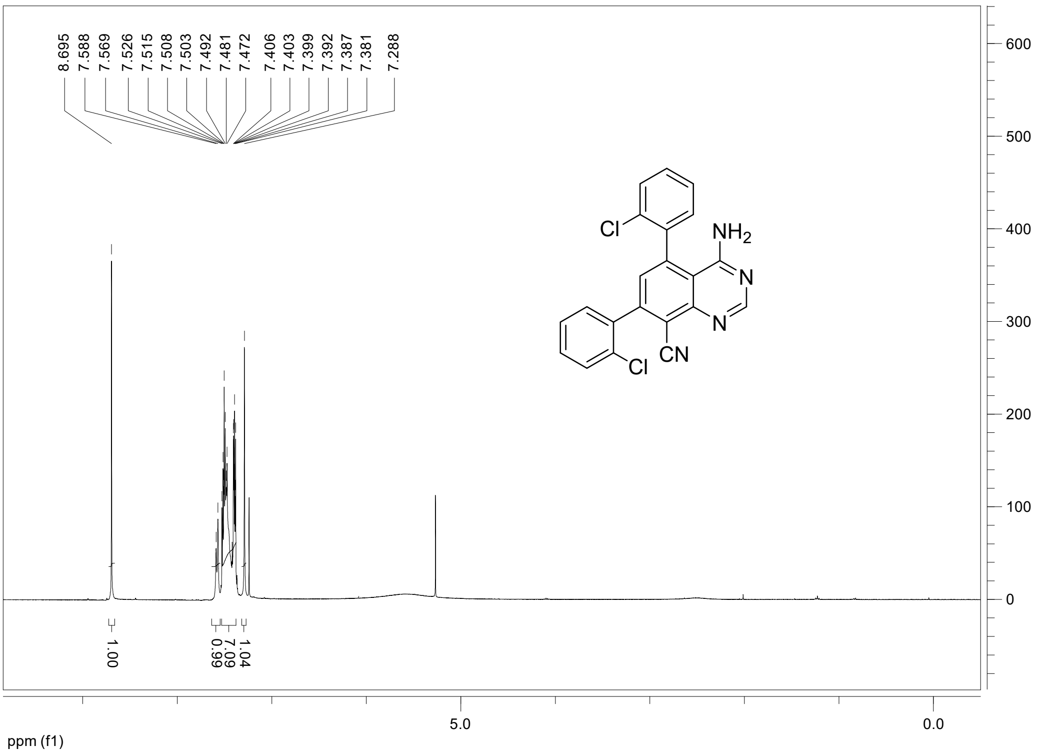

Supplementary Figure 35. Compound 7e: ^{1}H-NMR (400 MHz, $CDCl_3$)

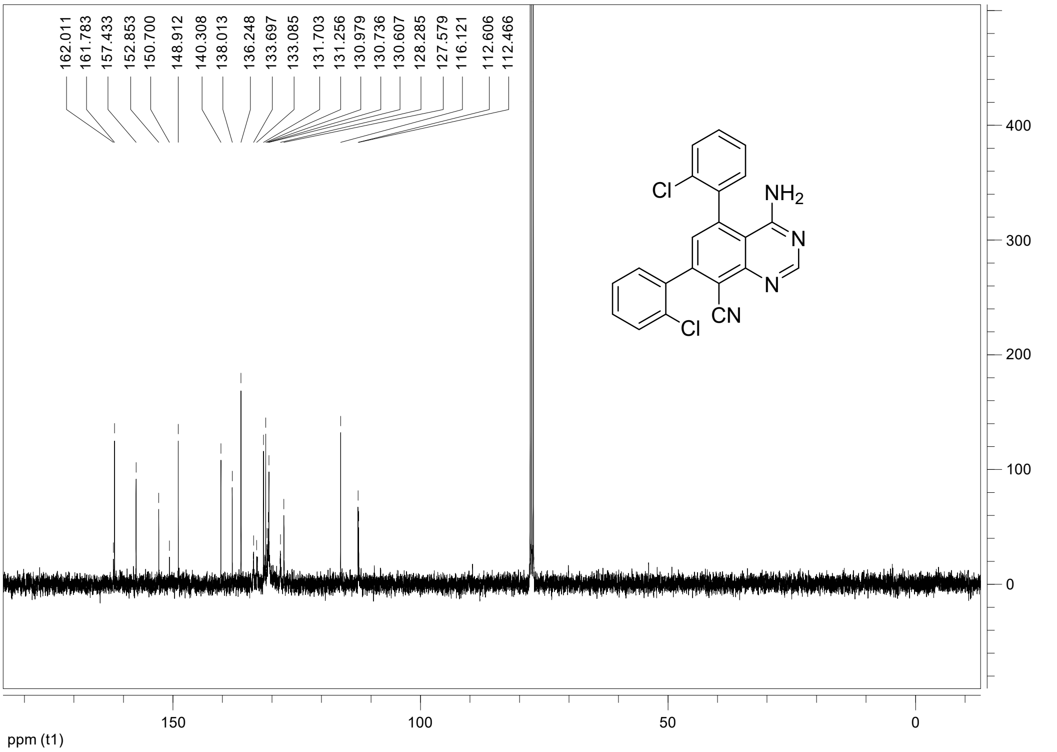

Supplementary Figure 36. Compound 7e: ^{13}C-NMR (100 MHz, $CDCl_3$)

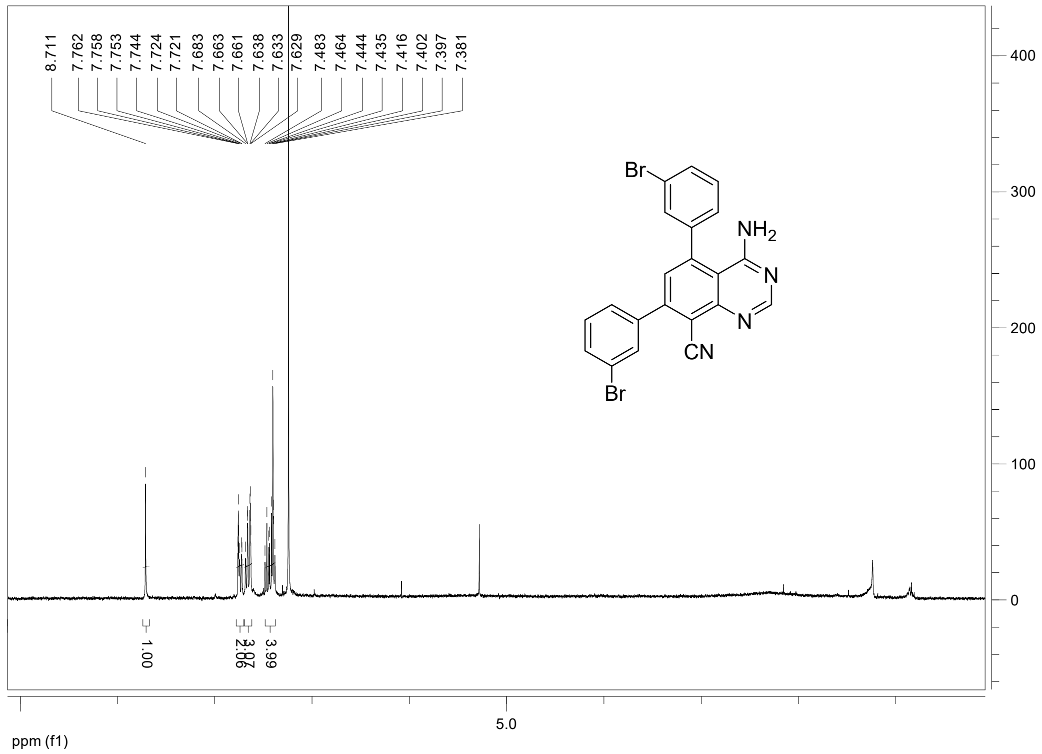

Supplementary Figure 37. Compound 7f: ^{1}H-NMR (400 MHz, $CDCl_3$)

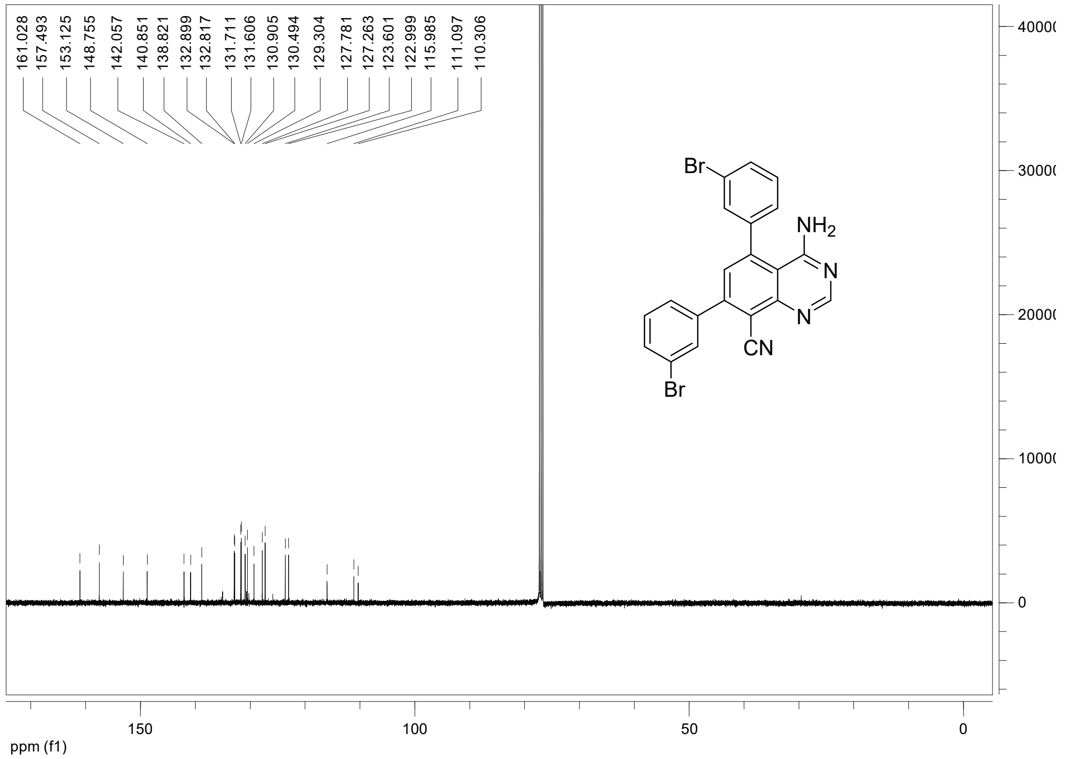

Supplementary Figure 38. Compound 7f: ^{13}C-NMR (100 MHz, $CDCl_3$)

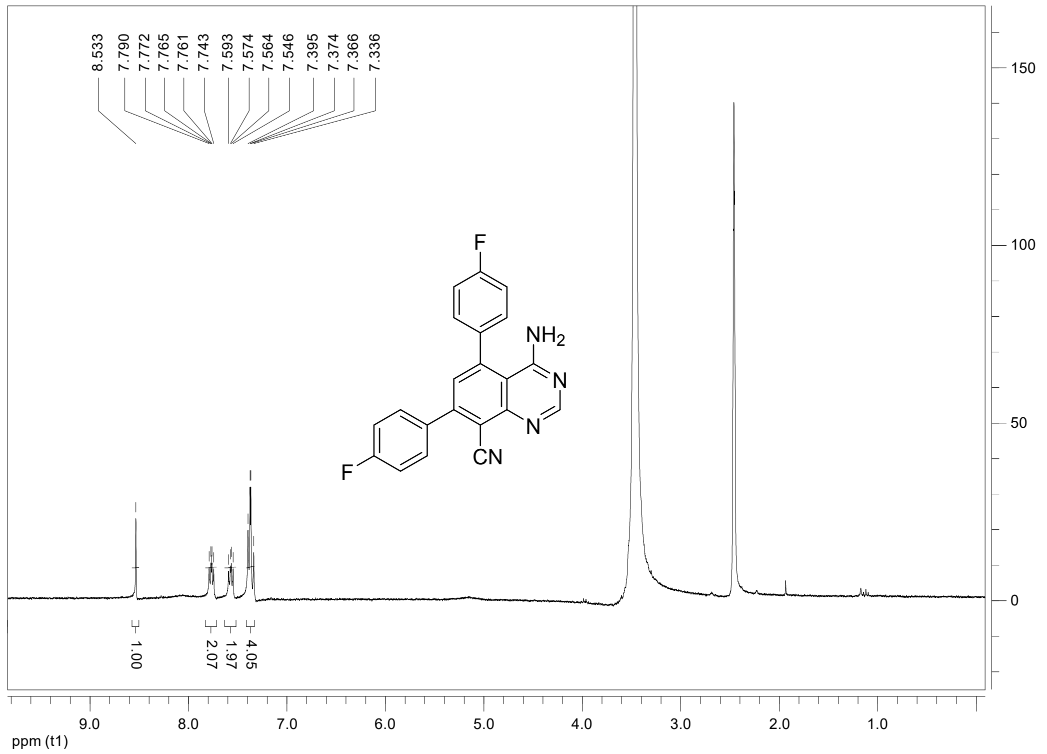

Supplementary Figure 39. Compound 7g: ^{1}H-NMR (400 MHz, DMSO$_{d6}$)

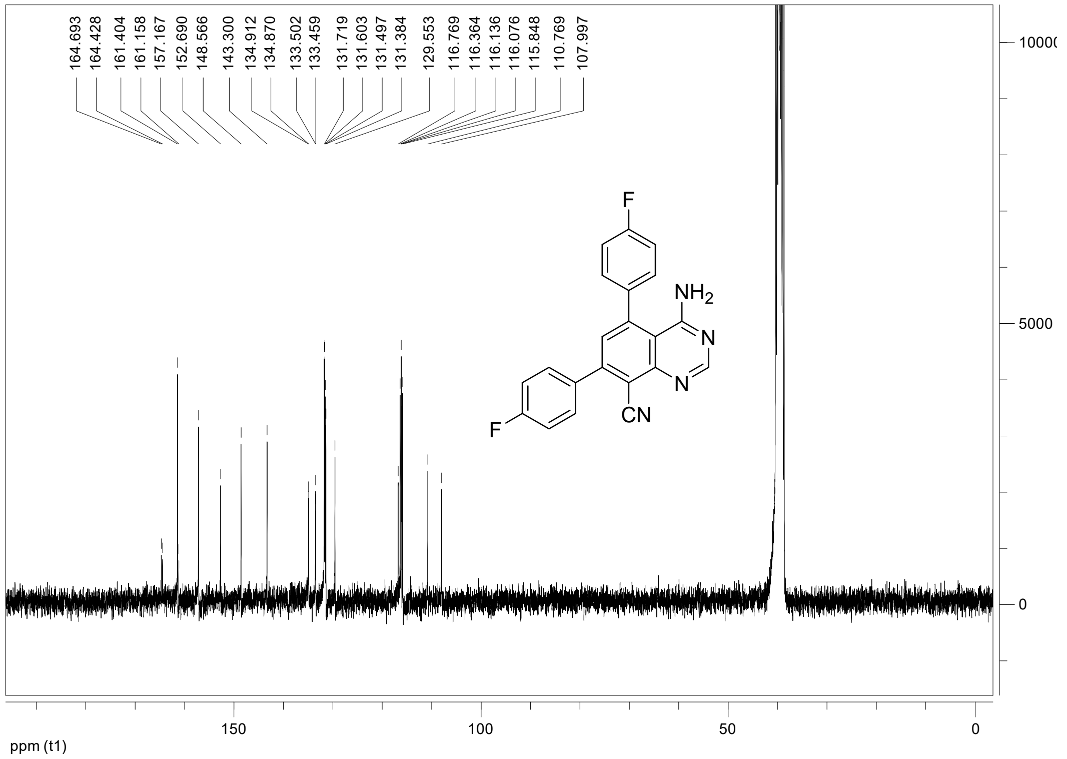

Supplementary Figure 40. Compound 7g: ^{13}C-NMR (100 MHz, DMSO$_{d6}$)

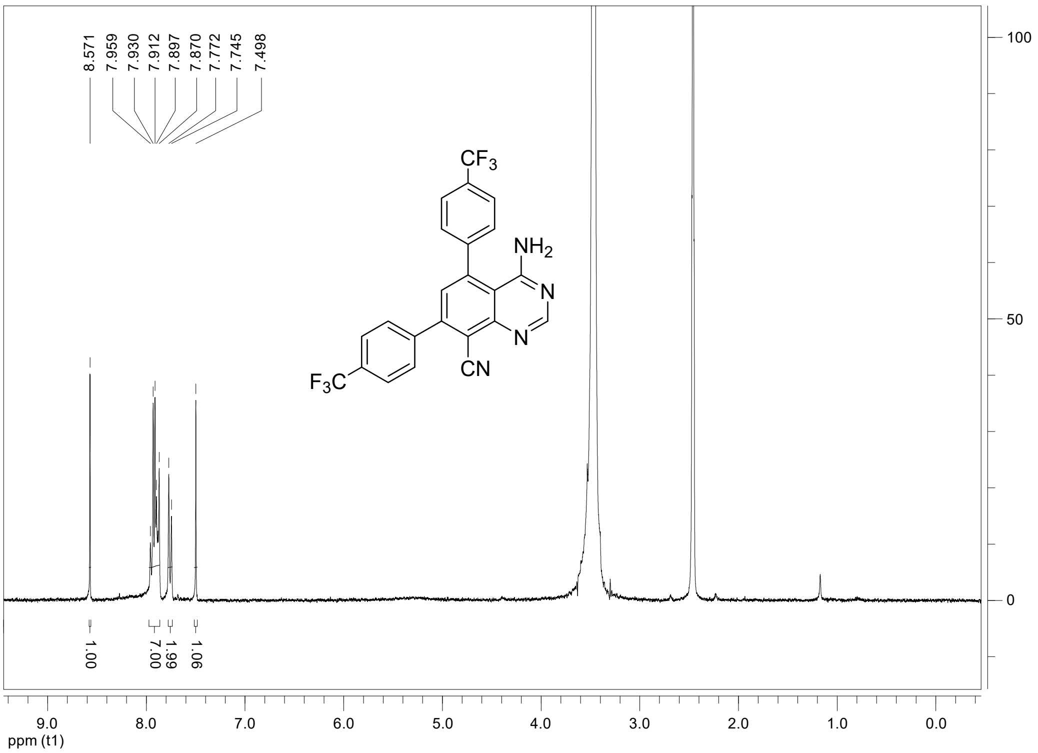

Supplementary Figure 41. Compound 7h: ^{1}H-NMR (400 MHz, DMSO$_{d6}$)

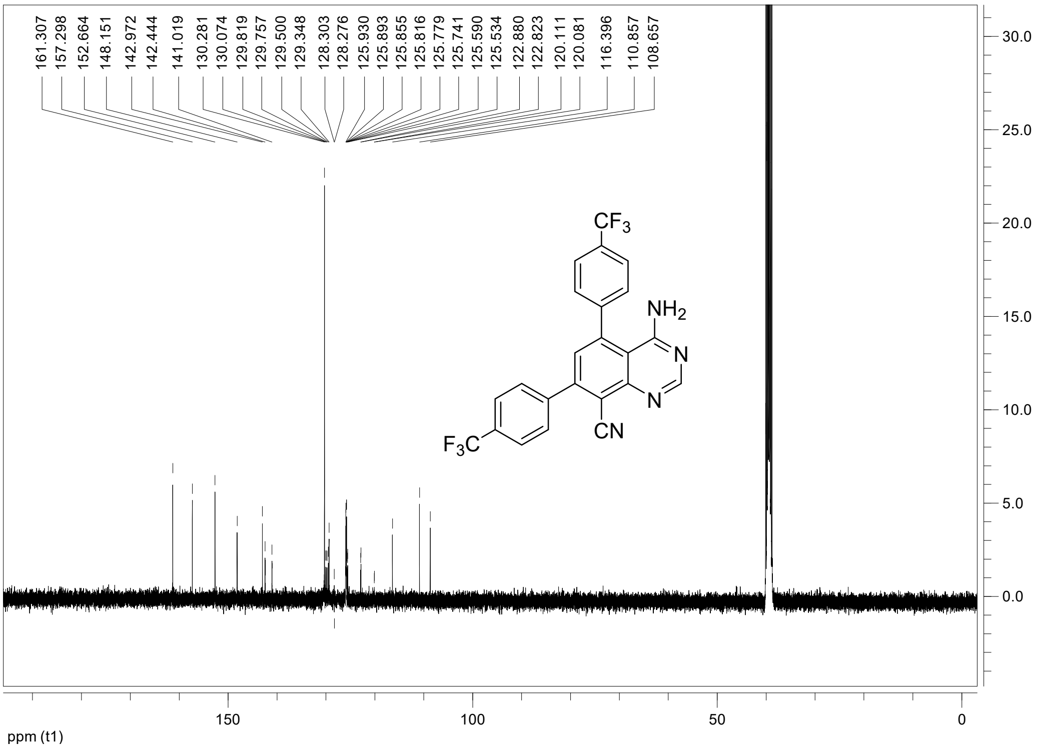

Supplementary Figure 42. Compound 7h: ^{13}C-NMR (100 MHz, DMSO$_{d6}$)

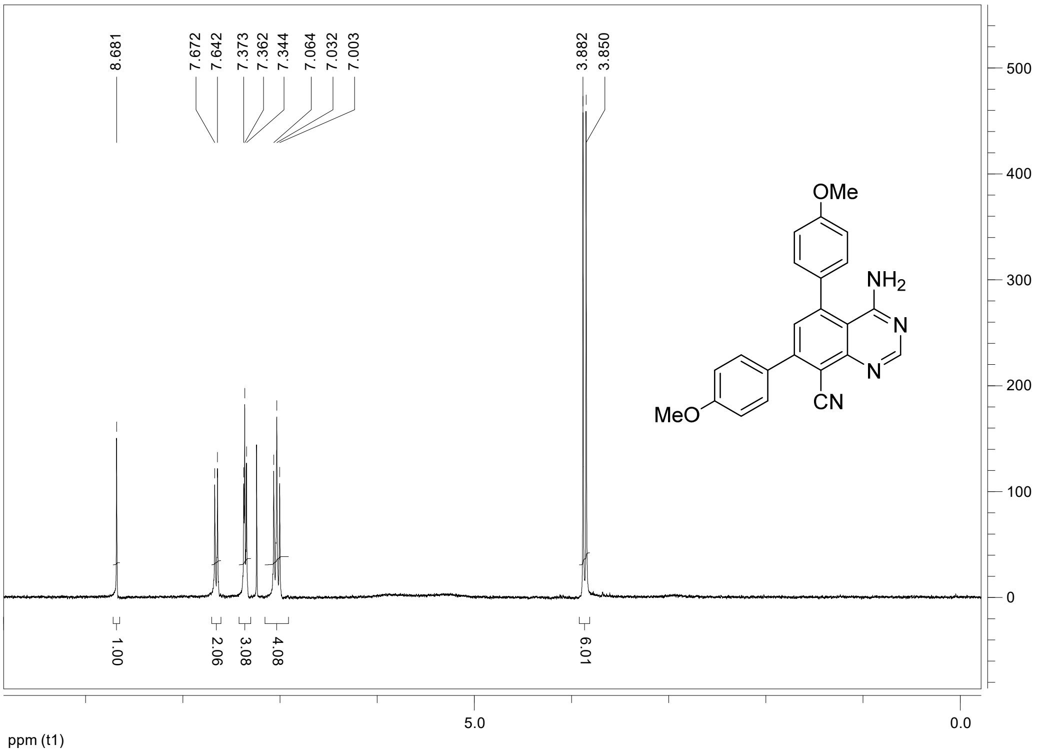

Supplementary Figure 43. Compound 7i: ^{1}H-NMR (400 MHz, $CDCl_3$)

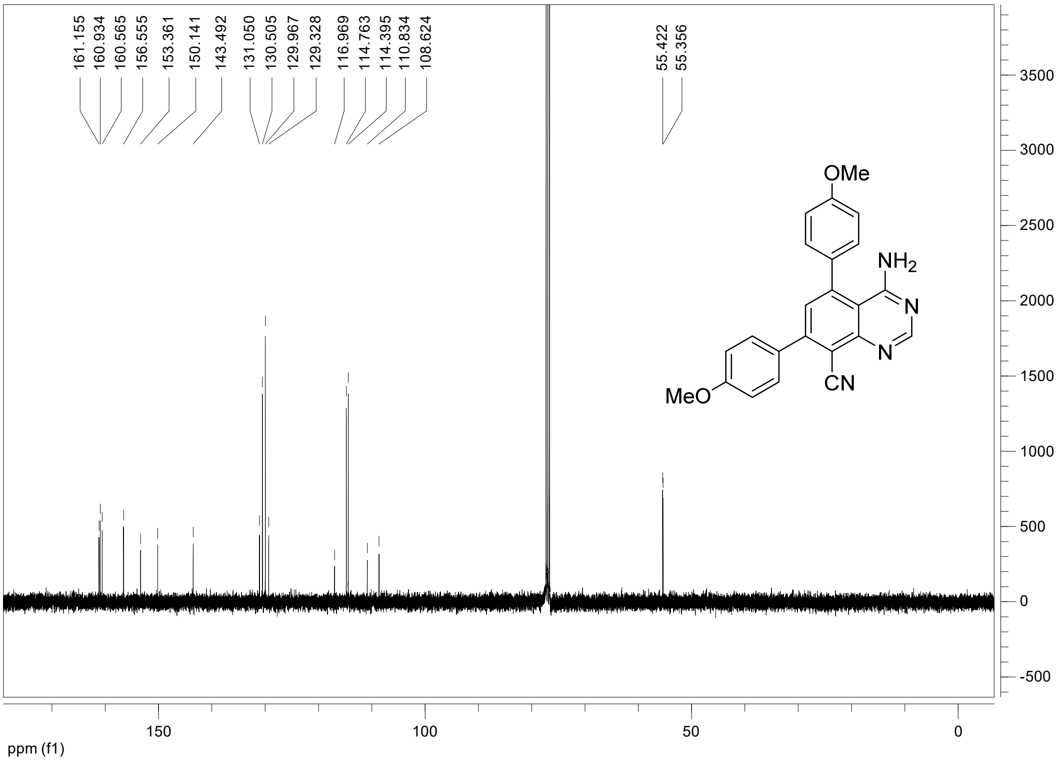

Supplementary Figure 44. Compound 7i: ^{13}C-NMR (100 MHz, $CDCl_3$)

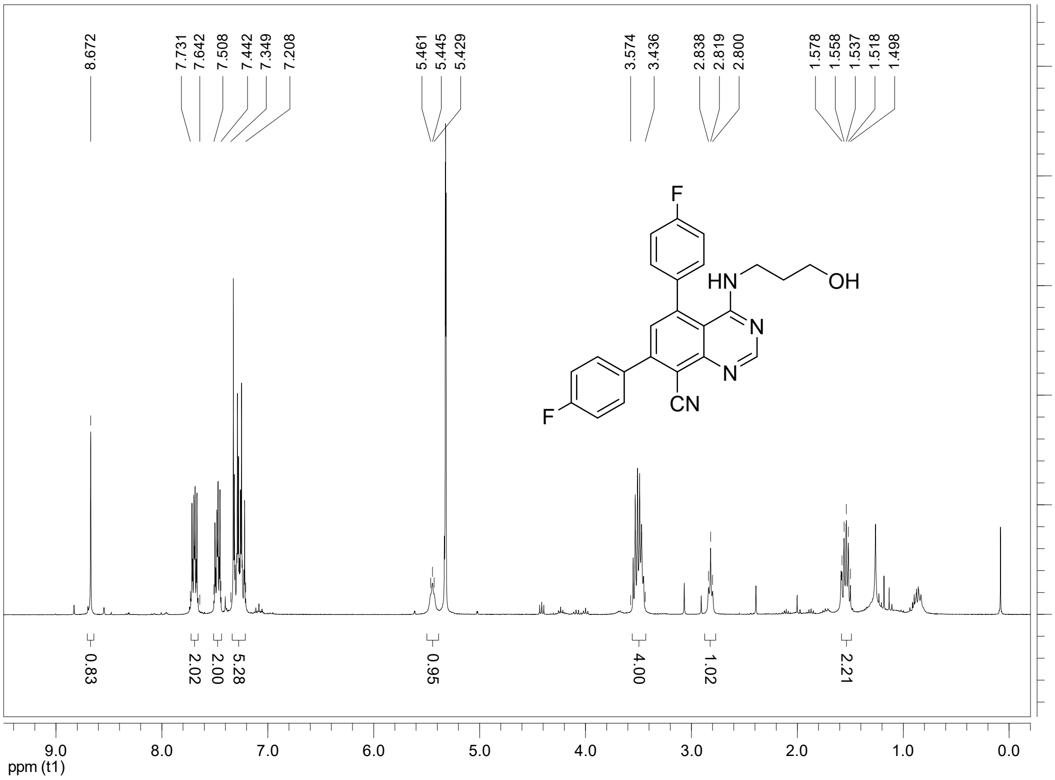

Supplementary Figure 45. Compound 10: ^{1}H-NMR (300 MHz, CD_2Cl_2)

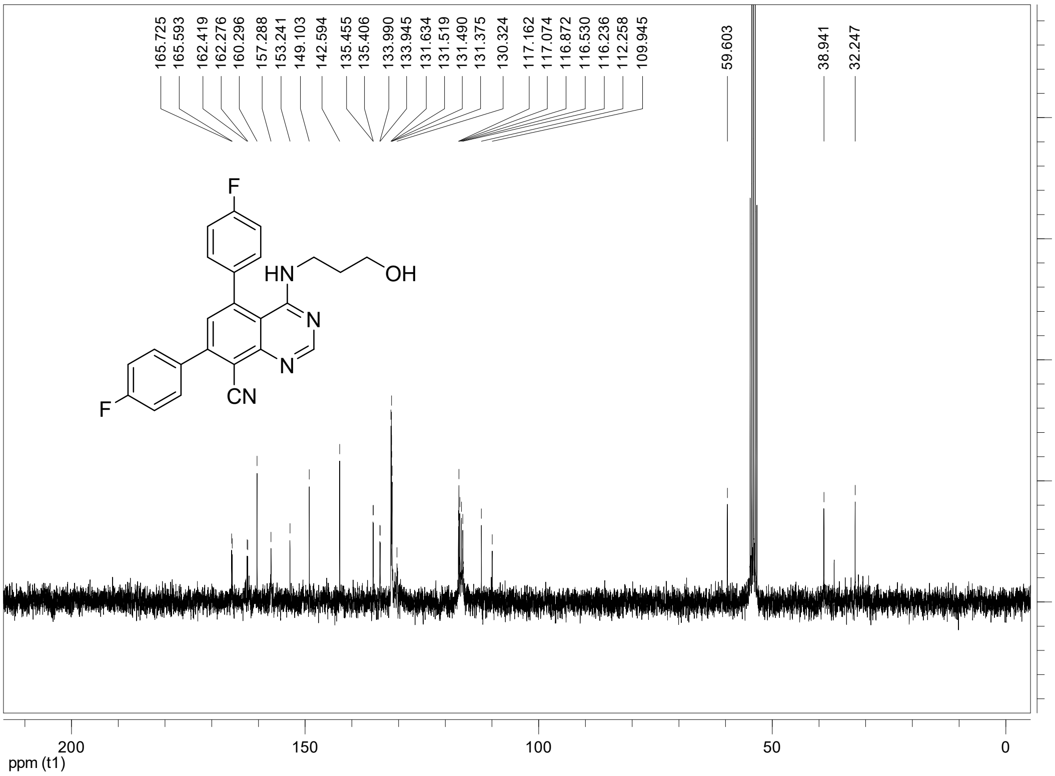

Supplementary Figure 46. Compound 10: ^{13}C-NMR (75 MHz, CD_2Cl_2)

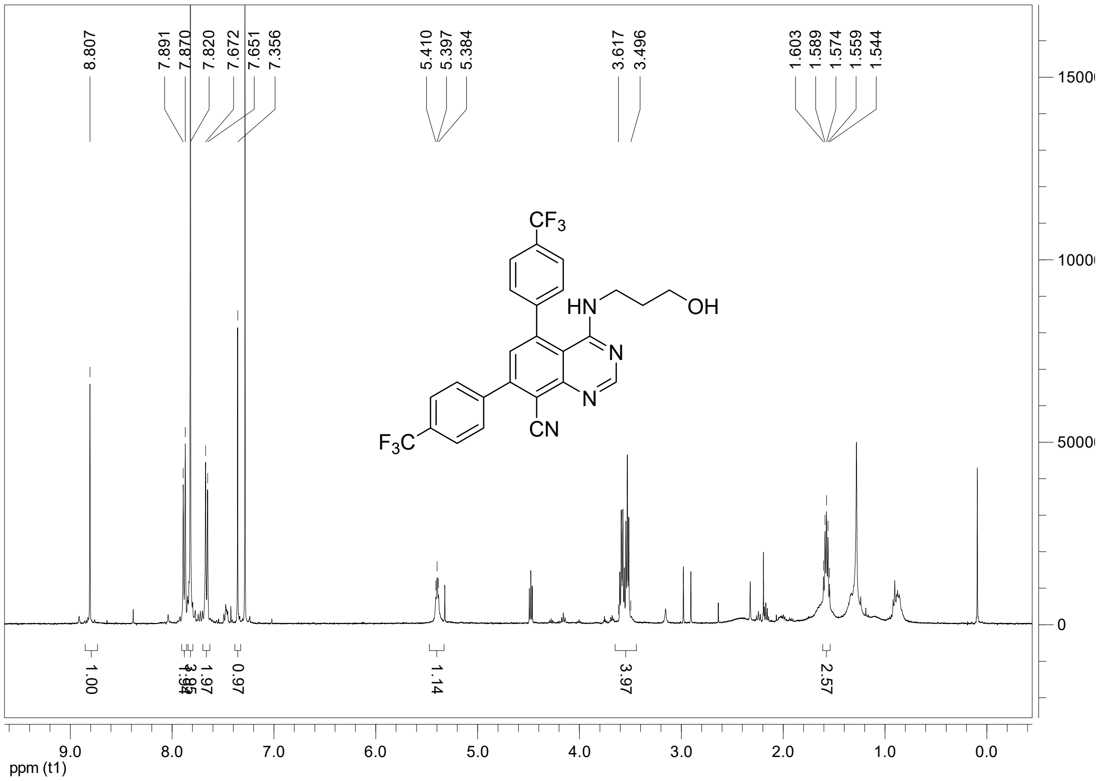

Supplementary Figure 47. Compound 11: ^{1}H-NMR (400 MHz, $CDCl_3$)

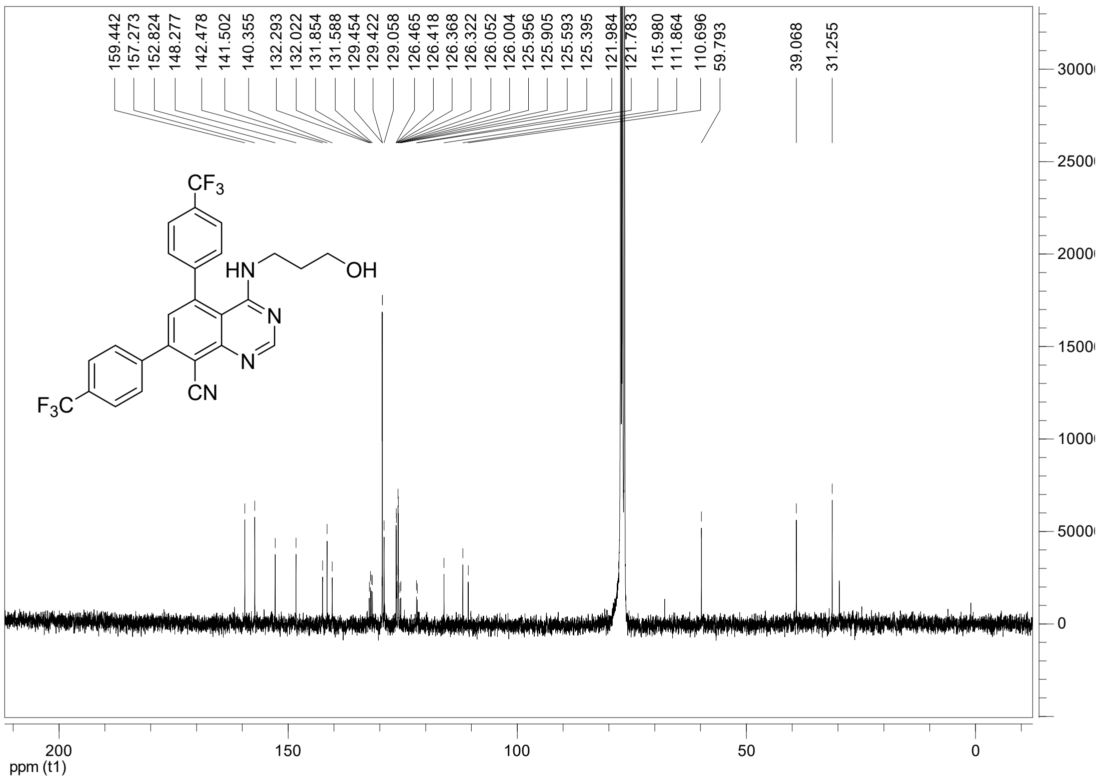

Supplementary Figure 48. Compound 11: ^{13}C-NMR (75 MHz, $CDCl_3$)

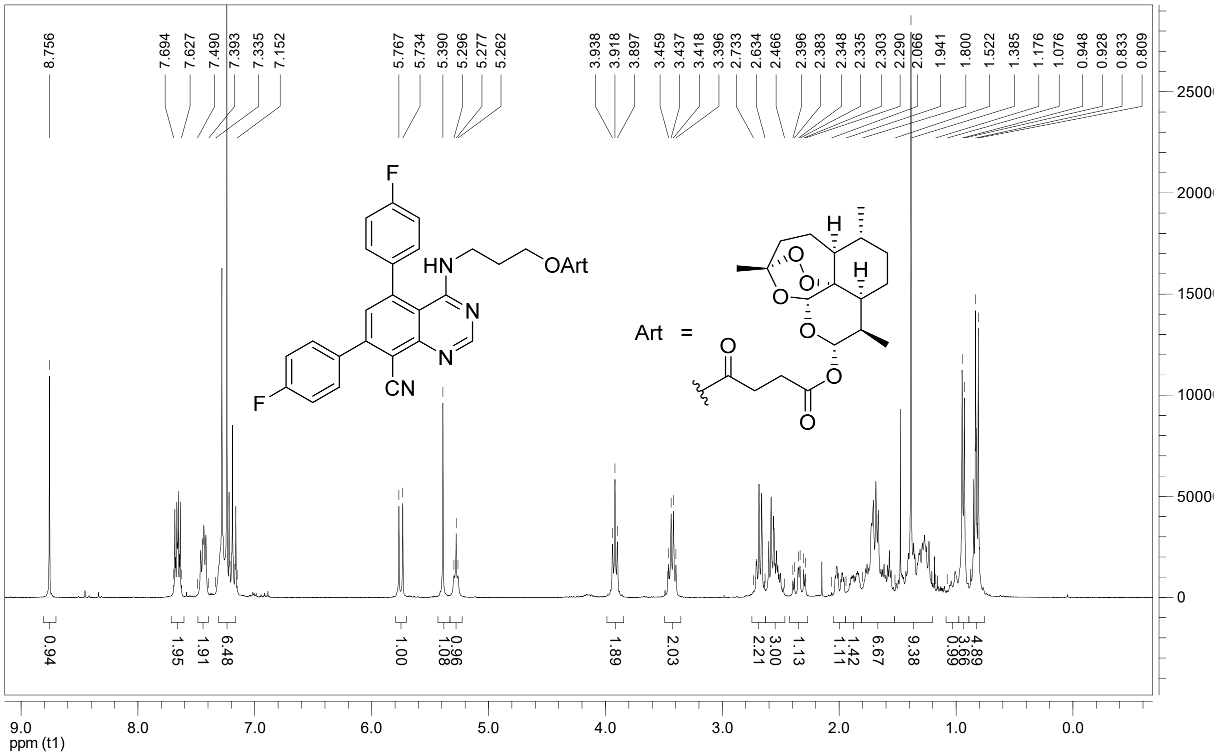

Supplementary Figure 49. Compound 8: ^{1}H-NMR (300 MHz, $CDCl_3$)

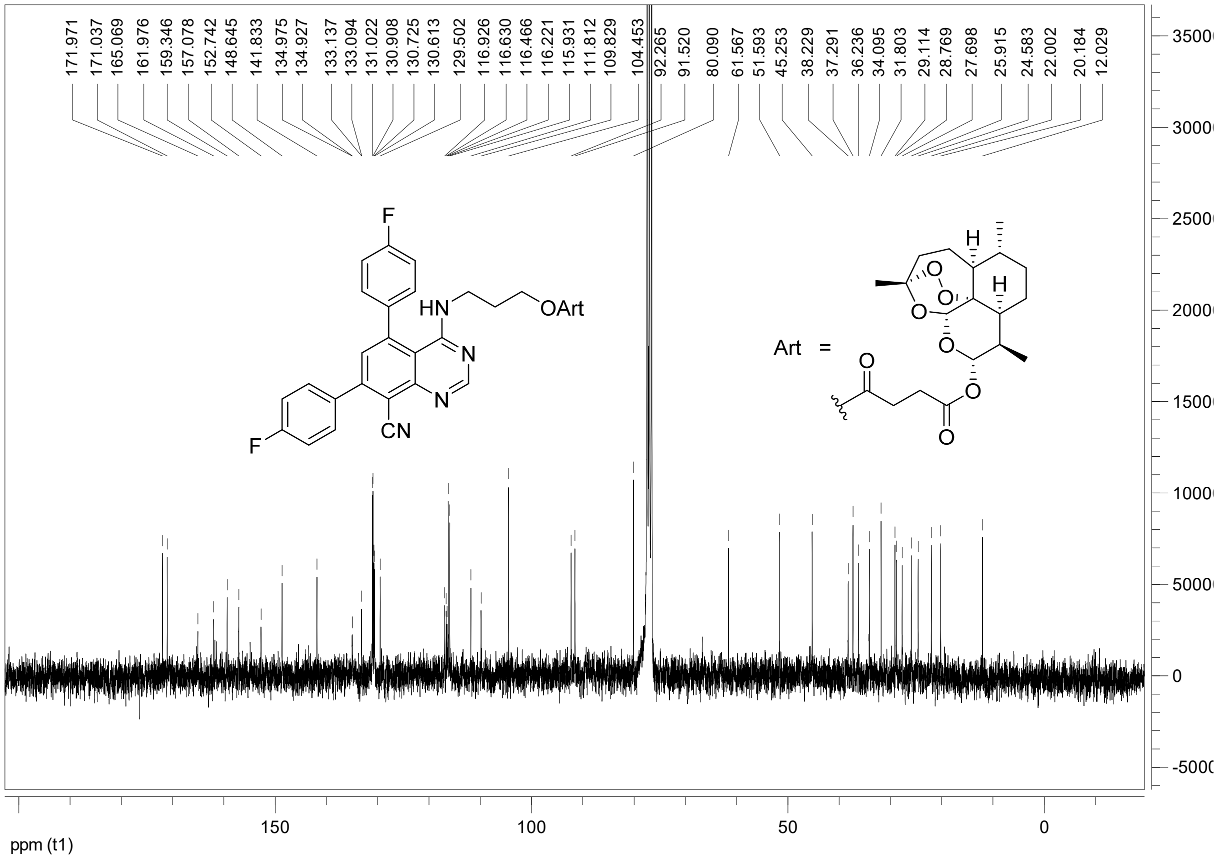

Supplementary Figure 50. Compound 8: ^{13}C-NMR (75 MHz, $CDCl_3$)

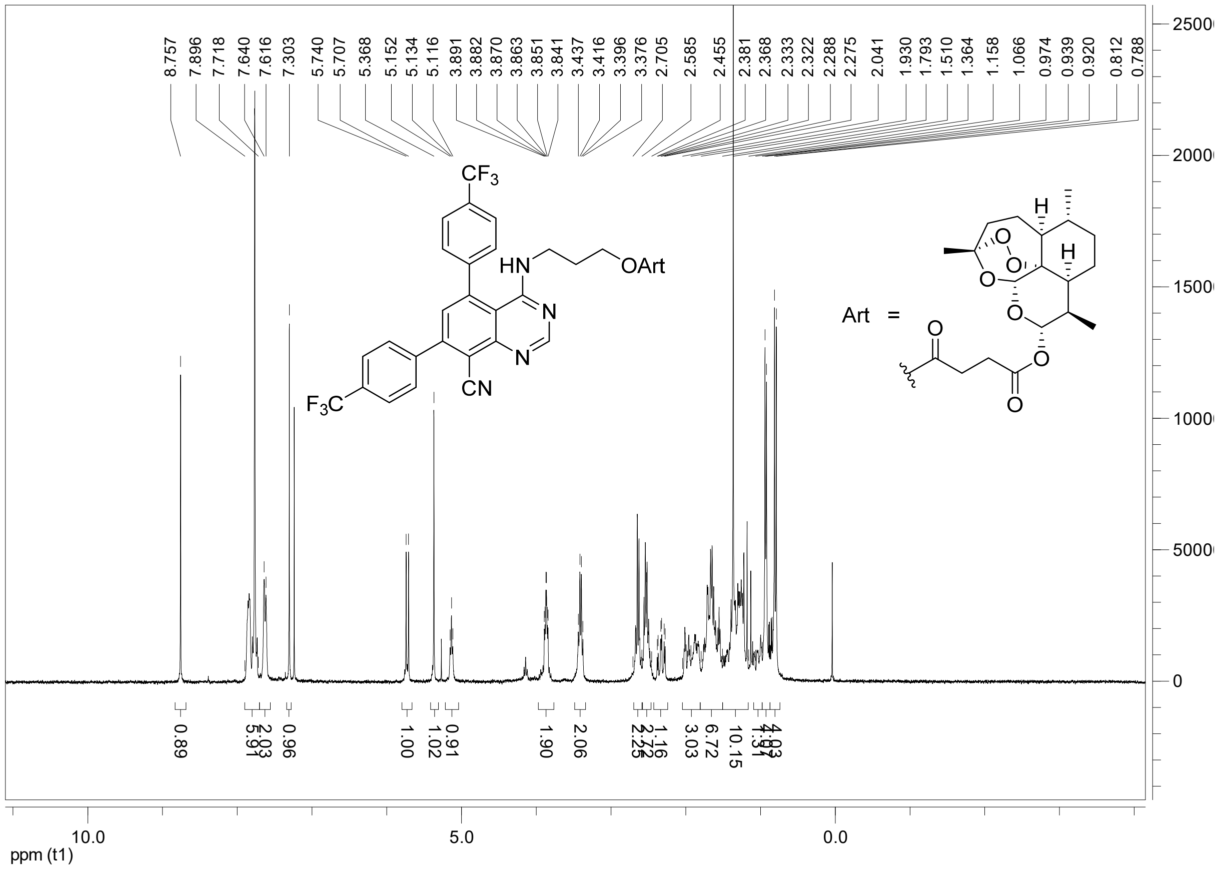

Supplementary Figure 51. Compound 9: ^{1}H-NMR (300 MHz, $CDCl_3$)

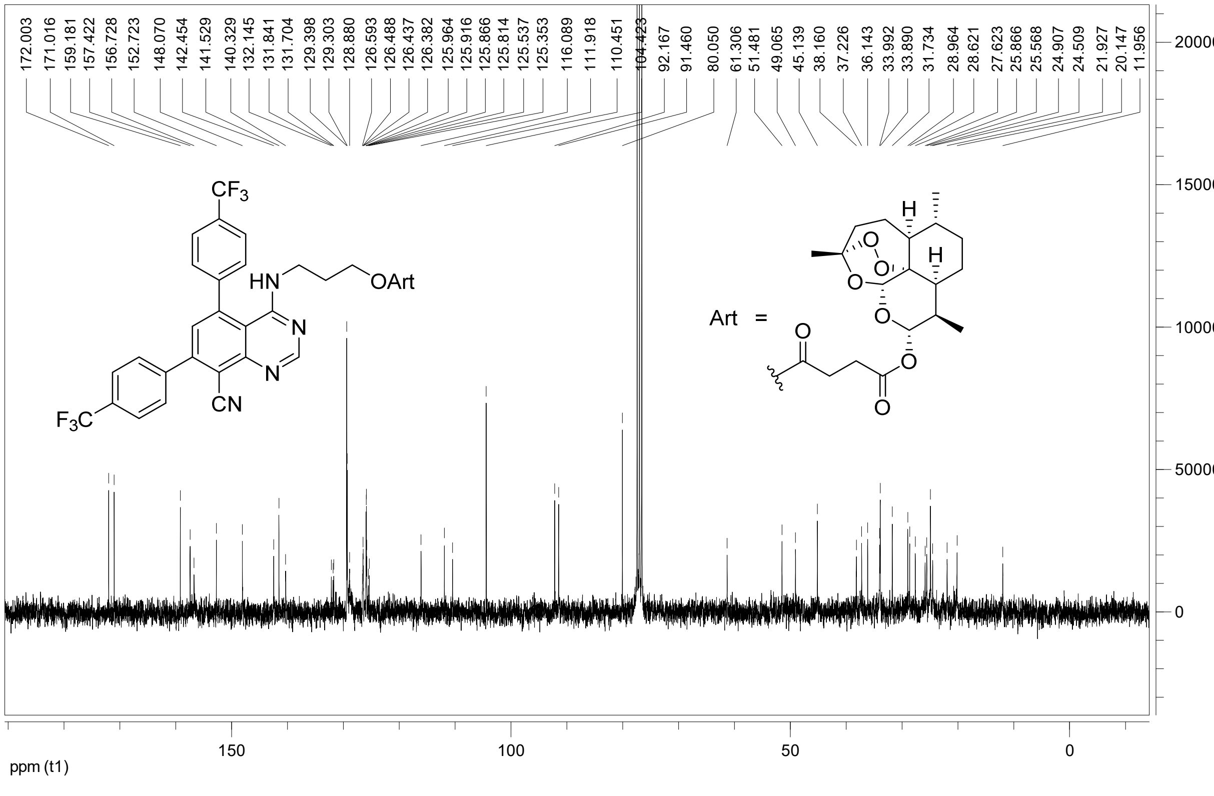

Supplementary Figure 52. Compound 9: ^{13}C-NMR (75 MHz, $CDCl_3$).

Supplementary Tables

Supplementary Table 1. Screening of reagents for aromatization of the domino product.

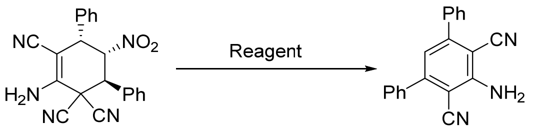

4a **5a**

Entry	Reagent	Solvent	T, °C	Conversion[a]/ Isolated Yield
1	DDQ (1 equiv)	ethyl acetate	80	traces on TLC
2	H_2O_2	-	r.t.	traces on TLC
3	MnO_2 (3 equiv)	toluene	120	traces on TLC
4	NaOMe (0.6 equiv)	methanol	r.t.	traces on TLC
5	H_2SO_4	-	100	no conversion
6	HNO_3	-	90	no conversion
7	ferric(III)nitrate (0.2 equiv)	toluene	80	no conversion
8	ammonium ceric (IV) nitrate (1 equiv)	formamide	150	some conversion visible on TLC
9	CuCl/TEMPO (0.05 equiv/0.05 equiv)	DMF	r.t.	no conversion
10	"bubbling air"	ethanol	r.t.	no conversion
11	AcOH / Py 1:1 ($^v/_v$)-mixture	-	80 °C	**good conversion on TLC: 41% isolated yield**

[a] determined *via* TLC

Supplementary Table 2. Crystal data and structure refinement for compound **4a**.

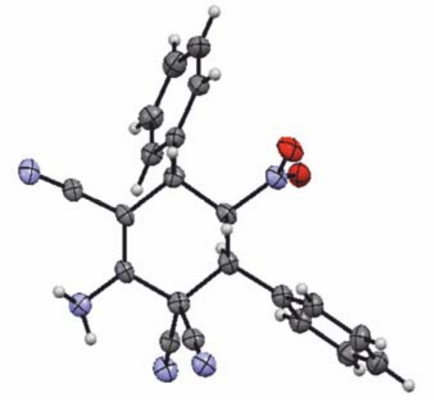

Colour scheme: carbon (grey), hydrogen (white), nitrogen (blue), oxygen (red).

Empirical formula	$C_{25}H_{23}N_4O_3$
Formula weight	427.47
Temperature/K	173.00(10)
Crystal system	monoclinic
Space group	P21/c
a/Å	6.7890(5)
b/Å	26.089(3)
c/Å	13.0807(17)
α/°	90.00
β/°	101.516(11)
γ/°	90.00
Volume/Å^3	2270.2(4)
Z	4
ρ_{calc}mg/mm^3	1.251
μ/mm^{-1}	0.681
F(000)	900.0
Crystal size/mm^3	0.309 × 0.1075 × 0.0819
2Θ range for data collection	6.78 to 124.34°
Index ranges	$-7 \le h \le 7$, $-17 \le k \le 29$, $-14 \le l \le 13$
Reflections collected	5808
Independent reflections	3431[R(int) = 0.0633]
Data/restraints/parameters	3431/0/293
Goodness-of-fit on F^2	1.081
Final R indexes [I>=2σ (I)]	R_1 = 0.0852, wR_2 = 0.1775

Final R indexes [all data]	$R_1 = 0.1579$, $wR_2 = 0.2376$
Largest diff. peak/hole / e Å^{-3}	0.28/-0.25

Supplementary Table 3. Crystal data and structure refinement for compound **4a'**.

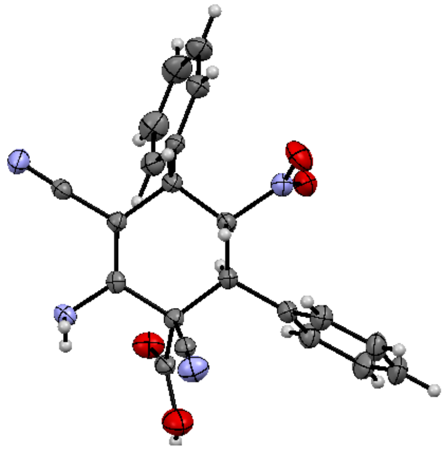

Colour scheme: carbon (grey), hydrogen (white), nitrogen (blue), oxygen (red).

Empirical formula	$C_{22.5}H_{22}N_4O_{5.5}$
Formula weight	436.44
Temperature/K	173.00(10)
Crystal system	triclinic
Space group	P-1
a/Å	10.5345(4)
b/Å	13.6669(4)
c/Å	15.7483(5)
α/°	103.058(3)
β/°	93.208(3)
γ/°	93.479(3)
Volume/Å^3	2198.99(12)
Z	4
ρ_{calc}mg/mm^3	1.318
μ/mm^{-1}	0.801
F(000)	916.0
Crystal size/mm3	0.169 × 0.1189 × 0.083
2Θ range for data collection	6.658 to 123.202°
Index ranges	$-12 \leq h \leq 11$, $-15 \leq k \leq 15$, $-17 \leq l \leq 17$

Reflections collected	27721
Independent reflections	6726[R(int) = 0.0440]
Data/restraints/parameters	6726/0/597
Goodness-of-fit on F2	1.035
Final R indexes [I>=2σ (I)]	R_1 = 0.0535, wR_2 = 0.1395
Final R indexes [all data]	R_1 = 0.0650, wR_2 = 0.1506
Largest diff. peak/hole / e Å^{-3}	0.80/-0.63

Supplementary Table 4. Crystal data and structure refinement for compound **7a**.

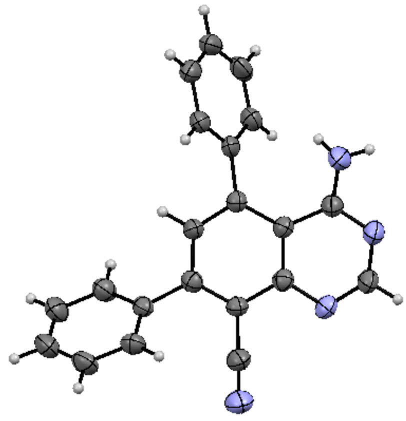

Colour scheme: carbon (grey), hydrogen (white), nitrogen (blue).

Empirical formula	$C_{21}H_{14}N_4$
Formula weight	322.36
Temperature/K	173.1(10)
Crystal system	triclinic
Space group	P-1
a/Å	9.5696(7)
b/Å	10.9245(7)
c/Å	18.4735(13)
α/°	74.255(6)
β/°	81.425(6)
γ/°	64.077(7)
Volume/Å^3	1670.7(2)
Z	4

ρ_{calc}g/cm^3	1.282
μ/mm^{-1}	0.620
F(000)	672.0
Crystal size/mm^3	0.1346 × 0.1038 × 0.0594
Radiation	CuKα (λ = 1.54184)
2Θ range for data collection/°	9.254 to 122.724
Index ranges	$-10 \leq h \leq 10$, $-12 \leq k \leq 9$, $-21 \leq l \leq 20$
Reflections collected	7387
Independent reflections	4936 [R_{int} = 0.0363, R_{sigma} = 0.0504]
Data/restraints/parameters	4936/0/453
Goodness-of-fit on F^2	1.034
Final R indexes [I>=2σ (I)]	R_1 = 0.0537, wR_2 = 0.1393
Final R indexes [all data]	R_1 = 0.0676, wR_2 = 0.1563
Largest diff. peak/hole / e Å^{-3}	0.35/-0.39

Supplementary Note 1

Fluorescent imaging with 5d. To prepare the donor slide we covered microscope glass substrates by radiation absorbing self-adhesive polyimide foil (Kapton, DuPont, USA; cmcKlebetechnik GmbH, Frankenthal/Pfalz, Germany; thickness of polyimide layer approx. 50 µm, thickness of glue layer approx. 45 µm). Then, 2 mg of the **5d** und 148 mg of the inert matrix polymer(SLEC PLT 7552, Sekisui Chemical GmbH, Düsseldorf / Germany) were dissolved in 1ml of dichloromethane and the spin coated (80 ppm, 40 s) over the irradiation absorbing laser. The transfer **5d** of occurred by local heating of the donor slide by laser irradiation that results in patterning of the spin-coated materials on the acceptor slide (see Supplementary Figure 2).

Supplementary Note 2

HCMV GFP-based replication assay and cell proliferation assay. The replication of human cytomegalovirus (HCMV) was determined by an established HCMV GFP-based replication assay as carried out over a duration of approx. seven days (multi-round infection) using cultures of primary human foreskin fibroblasts (HFFs). HFFs were cultivated in 12-well plates for the infection with a GFP-expressing recombinant human cytomegalovirus (HCMV AD169-GFP) and a signal quantitation using automated GFP fluorometry as described before.[2-4] All data represent mean values of determinations in quadruplicate (HCMV infections performed in duplicate, GFP measurements of total cell lysates performed in duplicate). Processing and evaluation of data was performed by the use of Excel (means and standard deviations, see Supplementary Figure 4 and Supplementary Data 1).

Supplementary Discussion

Basic Photophysical Study of Compound 7h. To shed light into the optical ground state properties of selected quinazoline **7h**, absorption spectra were measured in acetonitrile. In this solvent absorption in the UV region optical spectrum with a maximum at 350 nm and a not fully resolved absorption band below 300 nm are dominating (Supplementary Figure 3a). First insights into the excited states properties came from steady state fluorescence measurements. Compound **7h** exhibits in acetonitrile strong fluorescence between 370 and 600 nm (Supplementary Figure 3b) with a maximum at 439 nm and a fluorescence quantum yield of 0.15. The S_1-S_N transitions were investigated using femtosecond transient absorption spectroscopy, exciting **7h** in acetonitrile with femtosecond laser pulses at 258 nm. The excited singlet state is formed right after the laser pulse showing a broad transient absorption band with a maximum at 710 nm, accompanied by shoulders around 600 and 900 nm and a negative transient signal around 450 nm (Supplementary Figure 3c).

The observed transient absorption band belongs to the S_1-S_N transitions of **7h** and the observed minimum around 450 nm is best explained with the superimposition of the fluorescence giving a negative signal and the S_1-S_N transitions giving a positive transient absorption, with the former dominating around 450 nm. The first excited singlet state decays with a lifetime of 1490 ps into a new transient absorption maximizing at 510 nm (Supplementary Figure 3c / 3d). The best rationale for this finding is the intersystem crossing into the corresponding triplet manifold. In order to corroborate the triplet formation, nanosecond transient absorption measurements were conducted. The nanosecond transient absorption spectrum (Supplementary Figure 3e) exhibits maxima at 390 and 510 nm. This finding matches good with the femtosecond transient absorption spectrum after 6500 ps (Supplementary Figure 3c, cyan line) when taking into account that the fluorescence is not interfering with nanosecond transient absorption measurements in the microsecond time regime and that the detection of transient absorptions

in the femtosecond time domain is for our setup limited to the VIS and NIR part of the optical spectrum. The triplet nature of the transient absorption observed in Supplementary Figure 3e is confirmed by the oxygen sensitivity of this transient absorption. Supplementary Figure 3f for example depicts the absorption time profiles at 390 nm showing the decay of the triplet fist excited state under argon saturation with a lifetime of 4.6 µs (Supplementary Figure 3f, black curve) and under oxygen saturation (Supplementary Figure 3f, red curve), showing the expected oxygen sensitivity of triplet based transient absorptions.

Supplementary Methods

Photophysics

Steady state absorption and emission spectroscopy: Absorption spectra of compound **7h** (see Supplementary Figure 3) were measured with a Lambda 2 (Perkin Elmer) UV-vis spectrometer. The spectra were recorded between 300 and 600 nm at 240 nm per min with a 1.0 nm spectral bandwidth. The sample was contained in a 10 mm quartz cuvette. Fully corrected emission spectra were recorded on a FluoroMax-P spectrometer (Horiba JobinYvon). Fluorescence quantum yields were determined by the comparative method[5] using 9,10-diphenylanthracene as references with fluorescence quantum yields of 0.9, in cyclohexane.[6]

Transient absorption: Femtosecond transient absorption measurements were carried out with an amplified Ti/sapphire laser system CPA-2101 femtosecond laser (Clark MXR – output: 775 nm, 1 kHz and 150 fs pulse width) using a transient absorption pump / probe detection system (TAPPS Helios - Ultrafast Systems). The 258 nm excitation wavelength was generated by 3rd harmonic generation. Pulse widths of <150 fs and energies of 150 nJ / pulse were selected.

Nanosecond transient absorption laser photolysis measurements were performed with the output of the third harmonic (355 nm, 5 mJ / pulse) of a Nd:YAG laser (Brilliant B, Quantel). The optical detection is based on a pulsed (pulser MSP 05 – Optik Elektronik Müller) Xenon lamp (XBO 450, Osram), a monochromator (Spectra Pro 2300i, Acton Research), a R928 photomultiplier tube (Hamamatsu Photonics), or a fast InGaAs photodiode (Nano 5, Coherent) with 300-MHz amplification, and a 1-GHz digital oscilloscope (WavePro7100, LeCroy).

Cytotoxicity and Cell Proliferation Assays

Assays measuring distinct parameters of cytotoxicity and/or cell proliferation were performed as described earlier.[7, 8] In brief, a trypan blue exclusion assay was performed with cultured cells seeded in 24-well plates and incubated with increasing concentrations of antiviral compounds (range of 0.2 μM to 100 μM for 3 days). Staining of cells was achieved with 0.1% trypan blue for 10 min at room temperature before the percentage of viable cells was determined by microscopic counting (determination at least in triplicates). The cell proliferation assay (CellTiter 96 AQueous One Solution cell proliferation assay; Promega) was performed in a 96-well plate format after a 3-day treatment with compounds under standard conditions of measurement as described by the manufacturer.

Synthetic Methodologies

General Information. All chemicals used for synthesis were purchased from commercial sources and were used without further purification. All solvents were purified by distillation using rotary evaporation or were purchased in HPLC-grade-quality. All nitrostyrenes were prepared according to a literature procedure.[9] Spectral data of the nitrostyrenes matches literature values.[10, 11] All products were dried in high vacuum (up to 10^{-3} bar). Thin layer chromatography (TLC) was performed on pre-coated aluminum sheets ALUGRAM® SIL G/UV254 (0.2 mm silica gel with fluorescent indicator, MachereyNagel & Co). ^{1}H-NMR (^{13}C-NMR) spectra were recorded at room temperature on a Bruker Avance 300 or 400 or JEOL JNM GX 400 spectrometer operating at 300 MHz or 400 MHz (75 MHz or 100 MHz). All chemical shifts are given in the ppm-scale and refer to the nondeuterized proportion of the solvent. ESI and APPI mass spectra were recorded on a Bruker Daltonik maXis 4G or a Bruker Daltonik micrOTOF II focus. Elemental Analysis (C, H, N), carried out with an Elementar vario MICRO cube machine, is within ±0.40% of the calculated values confirming a purity of >95%. X-Ray crystallography was performed on a SuperNova, Dual, Cu at zero, Atlas diffractometer. Enantiomeric excesses (ee) were determined using analytical high performance liquid chromatography (HPLC) performed on an Agilent Technologies 1200 Series equipment provided with Agilent ChemStation, Standard and preparative Autosampler, Diode Array and Multiple Wavelength Detector SL, Thermostatted Column Compartment and a Daicel Chiralpak IA column.

General procedure for the domino reaction

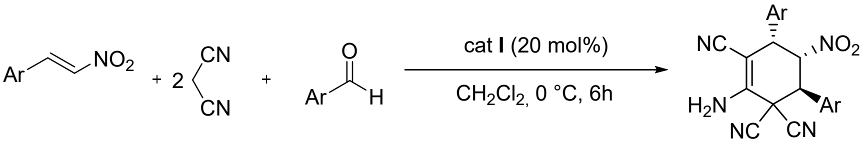

A round-bottomed flask equipped with a Teflon-coated magnetic stirring bar was charged with aromatic nitrostyrene (1 equiv), malononitrile (2 equiv), and aromatic aldehyde (1 equiv). To the mixture was added CH_2Cl_2 ([nitrostyrene] = 0.4M) at 0 °C. Then, catalyst **I** (0.2 equiv) was added and the reaction mixture was stirred for 6 h at 0 °C. The desired product was directly obtained by purification *via* column chromatography (SiO_2, *n*-hexane/EtOAc 7:1 – 5:1).

Characterization of the domino product 4a

(1'S,2'S,3'S)-5'-amino-2'-nitro-2',3'-dihydro-[1,1':3',1''-terphenyl]-4',4',6'(1'H)-tricarbonitrile 4a

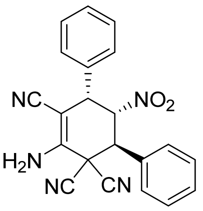

>99% yield; ^{1}H NMR (400 MHz, MeOD) δ: 4.06 (d, J = 12.7 Hz, 1H), 4.55 (d, J = 6.4 Hz, 1H), 6.06 (dd, J_1 = 6.4 Hz, J_2 = 12.7 Hz, 1H), 7.14-7.16 (m, 2H), 7.33-7.37 (m, 6H), 7.45-7.47 (m, 2H); ^{13}C NMR (100 MHz, $CDCl_3$) δ: 44.2, 45.4, 45.6, 78.2, 84.4, 112.5, 113.2, 118.0, 129.9, 129.9, 130.1, 130.2, 130.2, 130.9, 133.7, 136.4, 147.1; HRMS (ESI+) exact mass calcd. for $C_{21}H_{15}N_5NaO_2$ $[M+Na]^+$: 392.1118, found: 392.1112.

Procedure for the two-step aromatization of the domino product 4a

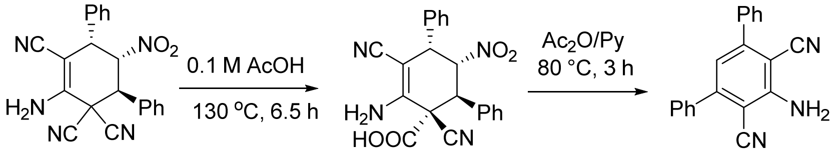

4a **4a'**, 75% yield **5a,** 95% yield

A round-bottomed flask equipped with a Teflon-coated magnetic stirring bar was charged with **4a** (1 equiv). AcOH ([**4a**] = 0.1M) was added and the reaction was heated at reflux (130 °C) for 6.5 h. The hydrolysed intermediate **4a'** was obtained by purification *via* column chromatography (SiO_2, *n*-hexane/EtOAc 2:1 – 1:1). **4a'** was then charged with Ac_2O/pyridine (80 equiv/40 equiv) and heated at 80 °C for 3 h. The desired product was obtained by purification *via* column chromatography (SiO_2, *n*-hexane/EtOAc = 10:1).

Characterization of cyclohexene derivative 4a’

(1'S,2'S,3'S,4'S)-5'-amino-4',6'-dicyano-2'-nitro-1',2',3',4'-tetrahydro-[1,1':3',1''-terphenyl]-4'-carboxylic acid 4a'

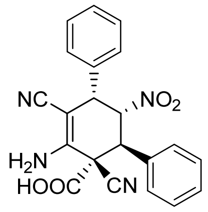

75% yield; ^1H NMR (300 MHz, MeOD) δ: 4.33 (d, *J*= 13.0 Hz, 1H), 4.46 (d, *J* = 6.15 Hz, 1H), 5.92 (dd, J_1 = 6.2, J_2 = 13.0, 1H), 7.23-7.32 (m, 10H); ^{13}C NMR (100 MHz, $CDCl_3$) δ: 44.2, 46.2, 56.2, 79.3, 85.8, 117.1, 118.8, 120.1, 129.9, 129.9, 130.0, 130.5, 135.5, 137.2, 151.5, 167.0.

General one-pot two-step procedure for the synthesis of 2,6-dicyanoanilines

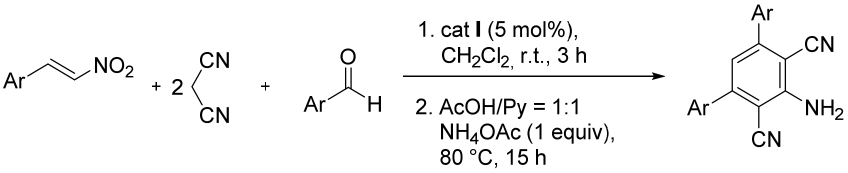

A round-bottomed flask equipped with a Teflon-coated magnetic stirring bar was charged with aromatic nitrostyrene (1 equiv), malononitrile (2 equiv), and aromatic aldehyde (1 equiv). A solution of catalyst **I** (0.05 equiv) in CH_2Cl_2 was added to the mixture at room temperature. The reaction mixture was stirred for 3 h. After evaporation of the solvent crude reaction mixture was dissolved in AcOH/pyridine (1:1 ($^v/_v$)-mixture, [nitrostyrene] = 0.4M) and NH_4OAc (1 equiv) was added. The reaction mixture was stirred at 80 °C for 15 h, after which time TLC indicated complete consumption of starting material. AcOH and pyridine were removed under reduced pressure. The desired product was obtained by purification *via* column chromatography (SiO_2, *n*-hexane/EtOAc) followed by recrystallization from MeOH.

Characterization of 2,6-dicyanoanilines

5'-amino-[1,1':3',1''-terphenyl]-4',6'-dicarbonitrile 5a

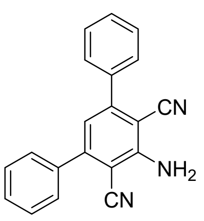

67% yield; column conditions: *n*-hexane/EtOAc = 10:1; MP 226 ºC; ^1H NMR (300 MHz, $CDCl_3$) δ: 5.42 (brs, 2H), 6.90 (s, 1H), 7.50-7.59 (m, 10H); ^{13}C NMR (100 MHz, $CDCl_3$) δ: 94.8, 115.9, 120.0, 128.4, 128.9, 129.7, 137.3, 150.1, 153.2; HRMS (APPI) exact mass calcd. for $C_{20}H_{13}N_3$ [M]: 295.1105, found: 295.1104.

5'-amino-4,4''-dimethyl-[1,1':3',1''-terphenyl]-4',6'-dicarbonitrile 5b

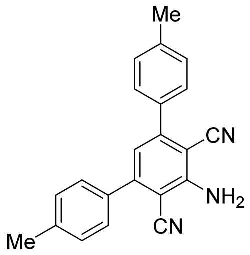

67% yield; column conditions: *n*-hexane/EtOAc = 10:1; MP 230 ºC; ^{1}H NMR (300 MHz, $CDCl_3$) δ: 2.43 (s, 6H), 5.38 (brs, 2H), 6.87 (s, 1H), 7.30 (d, *J* = 8.3 Hz, 4H), 7.48 (d, *J* = 8.1 Hz, 4H,); ^{13}C NMR (100 MHz, $CDCl_3$) δ: 21.3, 94.4, 116.2, 119.8, 128.3, 129.6, 134.6, 139.9, 150.0, 153.2; HRMS (APPI) exact mass calcd. for $C_{22}H_{17}N_3$ [M]: 323.1419, found: 323.1417.

5'-amino-4,4''-difluoro-[1,1':3',1''-terphenyl]-4',6'-dicarbonitrile 5c

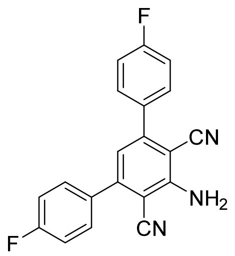

61% yield; column conditions: *n*-hexane/EtOAc = 10:1; MP 280 ºC (decomposition); ^{1}H NMR (300 MHz, $CDCl_3$) δ: 5.42 (brs, 2H), 6.82 (s, 1H), 7.20-7.23 (m, 4H), 7.54-7.59 (m, 4H); ^{13}C NMR (100 MHz, $CDCl_3$) δ: 94.9, 115.7, 116.1 (d, *J* = 22.0 Hz), 119.8, 130.3 (d, *J* = 8.0 Hz), 133.3 (d, *J* = 3.7 Hz), 149.1, 153.2, 162.4, 164.9; HRMS (APPI) exact mass calcd. for $C_{20}H_{11}F_2N_3$ [M]: 331.0921, found: 331.0916.

5'-amino-4,4''-bis(trifluoromethyl)-[1,1':3',1''-terphenyl]-4',6'-dicarbonitrile 5d

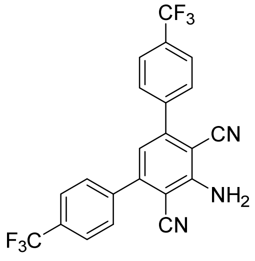

61% yield; column conditions: *n*-hexane/EtOAc = 12:1; MP = 282 ºC (decomposition); ^{1}H NMR (300 MHz, $CDCl_3$) δ: 5.54 (brs, 2H), 6.87 (s, 1H), 7.70 (d, *J* = 8.3 Hz, 4H), 7.79 (d, *J* = 8.3 Hz, 4H); ^{13}C NMR (100 MHz, $DMSO_{d6}$) δ: 94.7, 115.5, 118.4, 123.9 (q, *J* = 272.4 Hz), 125.4 (q, *J* = 3.8 Hz), 129.5, 129.8 (q, *J* = 32.1 Hz), 141.2 (q, *J* = 1.3 Hz), 148.4, 153.9; HRMS (APPI) exact mass calcd. for $C_{22}H_{11}F_6N_3$ [M]: 431.0849, found: 431.0852.

5'-amino-2,2''-dichloro-[1,1':3',1''-terphenyl]-4',6'-dicarbonitrile 5e

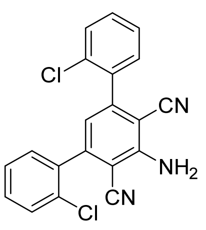

63% yield; column conditions: *n*-hexane/EtOAc = 10:1; MP = 250 ºC; ^{1}H NMR (400 MHz, $CDCl_3$) δ: 5.43 (brs, 2H), 6.77 (s, 1H), 7.39-7.42 (m, 6H), 7.52-7.54 (m, 2H); ^{13}C NMR (100 MHz, $DMSO_{d6}$) δ: 97.3, 114.9, 121.5, 127.0, 130.1, 130.4, 130.7, 132.4, 136.0, 147.4, 152.1; HRMS (APPI) exact mass calcd. for $C_{20}H_{11}Cl_2N_3$ [M]: 363.0329, found: 363.0325.

5'-amino-4,4''-dimethoxy-[1,1':3',1''-terphenyl]-4',6'-dicarbonitrile 5f

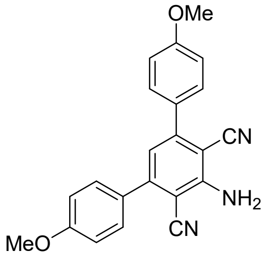

31% yield; column conditions: *n*-hexane/EtOAc = 14:1; MP = 242 ºC; ^{1}H NMR (300 MHz, $CDCl_3$) δ: 3.87 (s, 6H), 5.34 (bs, 2H), 6.84 (s, 1H), 7.02 (d, J = 8.9 Hz, 4H), 7.54 (d, J = 8.7 Hz, 4H); ^{13}C NMR (100 MHz, $CDCl_3$) δ: 55.4, 93.9, 114.3, 119.6, 129.7, 129.8, 129.9, 149.6, 153.3, 160.8; HRMS (APPI) exact mass calcd. for $C_{22}H_{17}N_3O_2$ [M]: 355.1316, found: 323.1315.

5'-amino-4,4''-bis(benzyloxy)-[1,1':3',1''-terphenyl]-4',6'-dicarbonitrile 5g

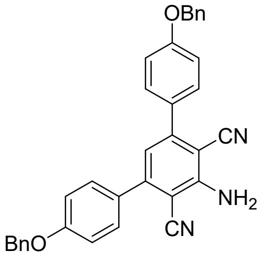

34% yield; column conditions: *n*-hexane/EtOAc = 10:1; ^{1}H NMR (400 MHz, $CDCl_3$) δ: 5.13 (s, 4H,), 5.37 (brs, 2H,), 6.84 (s, 1H), 7.10 (d, J = 8.6 Hz 4H), 7.34-7.47 (m, 10H), 7.55 (d, J = 8.6 Hz, 4H); ^{13}C NMR (100 MHz, $CDCl_3$) δ: 70.1, 93.9, 115.2, 116.4, 119.6, 127.5, 128.1, 128.6, 129.8, 129.9, 136.5, 149.5, 153.4, 160.0; HRMS (APPI) exact mass calcd. for $C_{34}H_{25}N_3O_2$ [M]: 507.1944, found: 507.1941.

5''-amino-[1,1':4',1'':3'',1''':4''',1''''-quinquephenyl]-4'',6''-dicarbonitrile 5f

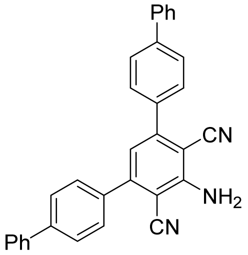

32% yield; column conditions: *n*-hexane/EtOAc = 8:1; ^{1}H NMR (300 MHz, $DMSO_{d6}$): 6.88-6.89 (m, 3H), 7.38-7.43 (m, 2H), 7.47-7.52 (m, 4H), 7.73-7.76 (m, 8H), 7.81-7.84 (m, 4H); ^{13}C NMR (100 MHz, $CDCl_3$): 93.8, 115.9, 118.3, 126.6, 126.7, 127.8, 128.9, 129.1, 136.3, 139.0, 141.0, 149.2, 154.1; HRMS (APPI) exact mass calcd. for $C_{32}H_{21}N_3$ [M]: 447.1732, found: 447.1730.

General one-pot procedure for the synthesis of quinazolines

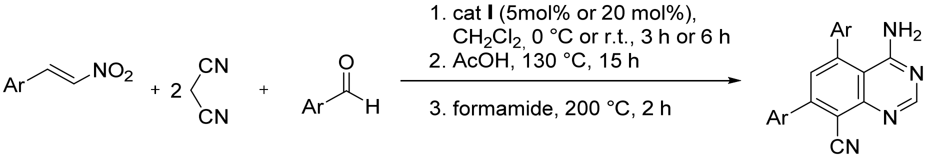

A round-bottomed flask equipped with a Teflon-coated magnetic stirring bar was charged with aromatic nitrostyrene (1 equiv), malononitrile (2 equiv), and aromatic aldehyde (1 equiv). To the mixture were added CH_2Cl_2 ([nitrostyrene] = 0.4M) at room temperature and catalyst **I** (0.2 equiv). The reaction mixture was stirred for 3 h. After evaporation of the solvent AcOH ([nitrostyrene] = 0.1M) was added

and the reaction was heated at reflux (130 °C) for 15 h, after which time TLC indicated complete consumption of starting material. AcOH was removed under reduced pressure, formamide (83 equiv) was added and the reaction was heated to 200 °C for 2 h. The reaction mixture was cooled to r.t. and poured into ice-water, extracted with ethyl acetate, washed with brine and dried over Na_2SO_4. The desired product was obtained by purification *via* column chromatography (SiO_2, *n*-hexane/EtOAc 2:1 – 1:1).

Characterization of quinazolines

4-amino-5,7-diphenylquinazoline-8-carbonitrile 7a

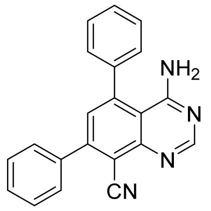

50% yield; ^1H NMR (400 MHz, $CDCl_3$) δ: 7.40 (s, 1H), 7.44-7.56 (m, 8H), 7.69 (2H, dd, J_1 = 1.6 Hz, J_2 = 7.9 Hz, ArCH), 8.72 (s, 1H); ^{13}C NMR (100 MHz, $CDCl_3$) δ: 109.5, 111.0, 116.6, 128.6, 128.9, 129.0, 129.4, 129.6, 129.7, 137.1, 139.2, 143.7, 150.4, 153.1, 154.6, 157.0, 161.2; HRMS (ESI+) exact mass calcd. for $C_{21}H_{15}N_4$ $[M+H]^+$: 323.1291, found: 323.1291, calcd. for $C_{21}H_{14}N_4Na$ $[M+Na]^+$: 345.1111, found: 345.1101.

4-amino-5,7-di-p-tolylquinazoline-8-carbonitrile 7b

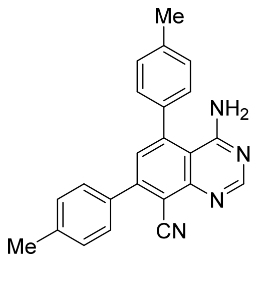

33% yield; MP 257 °C; ^1H NMR (300 MHz, $CDCl_3$) δ: 2.40 (s, 3H), 2.45 (s, 3H), 7.29-7.35 (m, 7H), 7.58 (d, J = 8.2 Hz, 2H), 8.68 (s, 1H); ^{13}C NMR (100 MHz, $CDCl_3$) δ: 21.3, 109.3, 111.0, 116.8, 128.6, 129.0, 129.8, 130.0, 130.2, 134.3, 136.2, 140.0, 140.1, 143.9, 150.8, 153.3, 156.4, 161.2; HRMS (ESI+) exact mass calcd. for $C_{23}H_{19}N_4$ $[M+H]^+$: 351.1606, found: 351.1606; anal. calcd. for $C_{23}H_{18}N_4$ x H_2O: C, 74.98; H, 5.47; N, 15.21; found: C, 75.14; H, 5.11; N, 14.81.

5,7-di([1,1'-biphenyl]-4-yl)-4-aminoquinazoline-8-carbonitrile 7c

20% yield; ^1H NMR (400 MHz, $DMSO_{d6}$) δ: 7.35-7.40 (m, 2H), 7.45-7.50 (m, 5H), 7.61-7.64 (m, 2H), 7.71-7.76 (m, 4H), 7.71-7.87 (m, 6H), 8.56 (s, 1H); ^{13}C NMR (100 MHz, $CDCl_3$) δ: 109.4, 111.1, 116.7, 127.1, 127.2, 127.7, 127.9, 128.0, 128.2, 128.9, 129.1, 129.2, 129.5, 129.7, 135.9, 137.9, 139.5, 140.1, 142.6, 142.7, 143.5, 150.0, 153.4, 157.2, 161.3; HRMS (ESI+) exact mass calcd. for $C_{33}H_{22}N_4$ $[M+H]^+$: 475.1910, found: 475.1917.

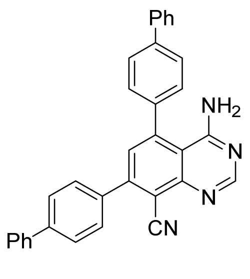

4-amino-5,7-bis(4-(tert-butyl)phenyl)quinazoline-8-carbonitrile 7d

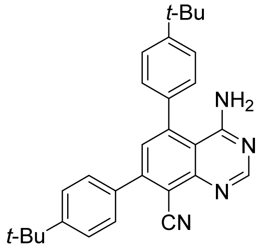

25% yield; ^{1}H NMR (400 MHz, $CDCl_3$) δ: 1.34 (s, 9H), 1.38 (s, 9H), 7.36 (d, J = 8.3 Hz, 2H), 7.40 (s, 1H), 7.51 (d, J = 8.4 Hz, 2H), 7.55 (d, J = 8.3 Hz, 2H), 7.64 (d, J = 8.4 Hz, 2H), 8.71 (s, 1H); ^{13}C NMR (100 MHz, $CDCl_3$) δ: 30.9, 31.0, 34.5, 34.6, 108.7, 110.8, 116.7, 125.6, 126.0, 128.1, 128.5, 129.7, 133.9, 136.0, 143.5, 150.1, 152.7, 152.7, 153.1, 156.8, 161.1; HRMS (ESI+) exact mass calcd. for $C_{29}H_{31}N_4$ $[M+H]^+$: 435.2560, found: 435.2543.

4-amino-5,7-bis(2-chlorophenyl)quinazoline-8-carbonitrile 7e

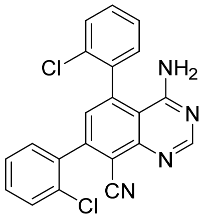

40% yield; ^{1}H NMR (400 MHz, $CDCl_3$) δ: 7.28 (s, 1H), 7.38-7.41 (m, 2H), 7.47-7.53 (m, 5H), 7.58 (d, J = 7.7 Hz, 2H), 8.70 (s, 1H); ^{13}C NMR (100 MHz, $CDCl_3$) δ: 112.5, 112.6, 116.1, 127.6, 128.3, 130.6, 130.7, 131.0, 131.3, 131.7, 133.1, 133.7, 136.2, 138.0, 140.3, 148.9, 150.7, 152.9, 157.4, 161.8, 162.0; HRMS (ESI+) exact mass calcd. for $C_{21}H_{13}Cl_2N_4$ $[M+H]^+$: 391.0512, found: 391.0506.

4-amino-5,7-bis(3-bromophenyl)quinazoline-8-carbonitrile 7f

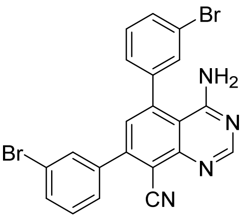

40% yield; ^{1}H NMR (400 MHz, $CDCl_3$) δ: 7.33 (s, 1H), 3.38-7.48 (m, 4H), 7.63-7.66 (m, 3H), 7.72-7.76 (m, 2H); ^{13}C NMR (100 MHz, $CDCl_3$) δ: 110.3, 111.1, 116.0, 123.0, 123.6, 127.3, 127.8, 129.3, 130.5, 130.9, 131.6, 131.7, 132.8, 132.9, 138.8, 140.8, 142.0, 148.8, 153.1, 157.5, 161.0; HRMS (ESI+) exact mass calcd. for $C_{21}H_{13}Br_2N_4$ $[M+H]^+$: 435.2560, found: 435.2543.

4-amino-5,7-bis(4-fluorophenyl)quinazoline-8-carbonitrile 7g

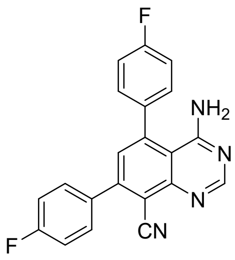

35% yield; MP 300 °C (decomposition); ^{1}H NMR (400 MHz, $DMSO_{d6}$) δ: 7.34-7.40 (m, 4H), 7.55-7.59 (m, 2H), 7.74-7.79 (m, 2H), 8.53 (s, 1H); ^{13}C NMR (100 MHz, $DMSO_{d6}$) δ: 108.0, 110.8, 116.1 (dd, J_1 = 17.2 Hz, J_2 = 21.7 Hz), 116.8, 129.5, 131.6 (dd, J_1 = 8.6 Hz, J_2 = 16.7 Hz), 134.2 (dd, J_1 = 3.2 Hz, J_2 = 106.4 Hz), 143.3, 148.6, 152.7, 157.2, 161.4, 162.9 (dd, J_1 = 20.0 Hz, J_2 = 247.5 Hz); ^{19}F NMR (282 MHz, $DMSOd_6$) δ: -60.8, -60,7; HRMS (ESI+) exact mass calcd. for $C_{21}H_{13}F_2N_4$ $[M+H]^+$: 359.1103, found: 359.1096; anal. calcd. for $C_{21}H_{12}F_2N_4$: C, 70.39; H, 3.38; N, 15.63; found: C, 69.86; H, 3.29; N, 15.50.

4-amino-5,7-bis(4-(trifluoromethyl)phenyl)quinazoline-8-carbonitrile 7h

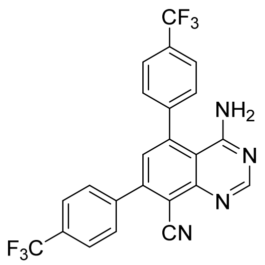

25% yield; MP 290 °C (decomposition); ^{1}H NMR (300 MHz, DMSO$_{d6}$) δ: 7.50 (s, 1H), 7.75-7.77 (m, 2H), 7.88-8.00 m, (m, 6H), 8.57 (s, 1H); ^{13}C NMR (100 MHz, DMSO$_{d6}$) δ: 108.7, 110.9, 116.4, 124.1 (dq, J_1 = 5.6 Hz, J_2 = 272.6 Hz) 125.7-125.9 (m), 129.3, 129.8 (dd, J_1 = 25.7 Hz, J_2 = 32.0 Hz), 130.3, 141.0, 142.4, 142.9, 148.1, 152.6, 157.2, 161.2; HRMS (MALDI+) exact mass calcd. for $C_{23}H_{13}F_6N_4$ $[M+H]^+$: 459.1039, found: 459.1045; exact mass calcd. for $C_{23}H_{12}F_6N_4Na$ $[M+Na]^+$: 481.0862, found: 481.0862; anal. calcd. for $C_{23}H_{12}F_6N_4$: C, 60.27; H, 2.64; N, 12.22; Found: C, 60.23; H, 2.79; N, 12.07.

4-amino-5,7-bis(4-methoxyphenyl)quinazoline-8-carbonitrile 7i

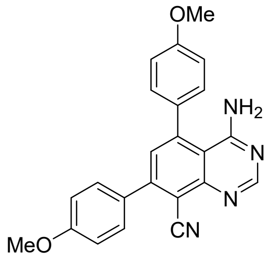

20% yield; MP 254 °C; ^{1}H NMR (400 MHz, $CDCl_3$) δ: 3.85 (s, 3H), 3.88 (s, 3H), 7.03-7.06 (m, 4H), 7.34-7.37 (m, 3H), 7.66 (d, J = 8.8 Hz, 2H), 8.68 (s, 1H); ^{13}C NMR (100 MHz, $CDCl_3$) δ: 55.4, 55.4, 108.6, 110.8, 114.4, 114.8 , 117.0, 129.3, 130.0, 130.5, 131.1, 143.5, 150.1, 153.4, 156.6, 160.6, 160.9, 161.2; HRMS (ESI+) exact mass calcd. for $C_{23}H_{19}N_4O_2$ $[M+H]^+$: 383.1503, found: 383.1503; anal. calcd. for $C_{23}H_{18}N_4O_2$: C, 72.24; H, 4.74; N, 14.65; found: C, 71.72; H, 4.81; N, 14.84.

Procedure for the synthesis of artesunic acid-quinazoline hybrids 8/9

The synthesis of the target compounds **8** and **9** (s. Scheme below) is very straightforward and starts with simple alkylation of quinazolines **7g** or **7h** at the free amine group using 3-bromopropanol as electrophile, $CsCO_3$ as base and a 4:1 mixture of CH_3CN/DMF as solvent. After refluxing for 18 h the product could be isolated in 60%/54% yield. Subsequent Steglich esterification using DCC and DMAP as coupling agents and CH_2Cl_2 as solvent afforded the desired artesunic acid-quinazoline hybrids **8** and **9** in 78% / 81% yield.

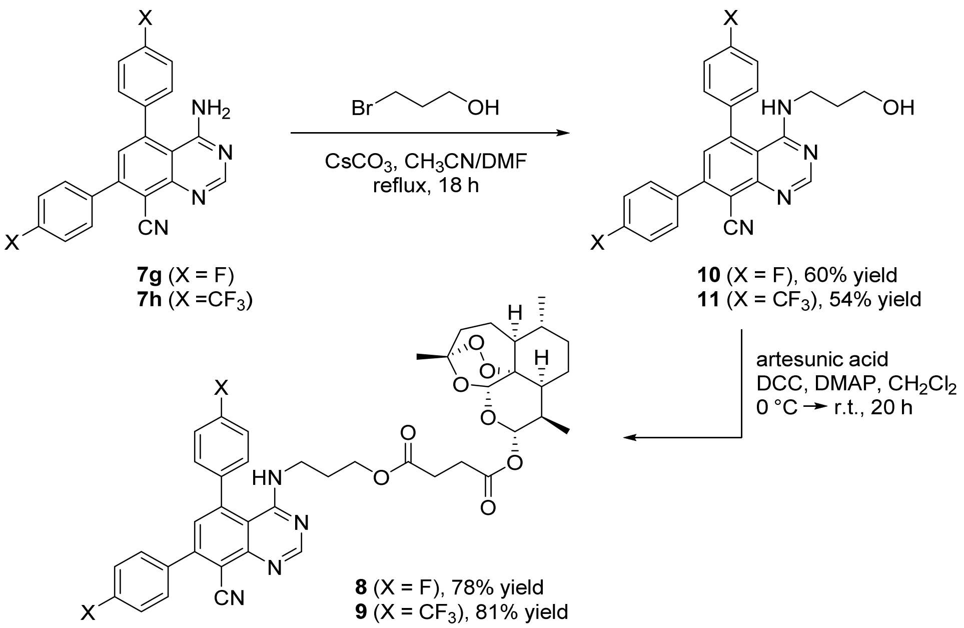

General procedure for the synthesis of quinazoline alcohols 10 and 11

To a solution of either quinazoline **7g** (118 mg, 0.33 mmol, 1 equiv) or **7h** (50.0 mg, 0.11 mmol, 1 equiv) in a 4:1 mixture of CH_3CN and DMF (10.0 mL) $CsCO_3$ (2 equiv) and 3-bromopropanol (5 equiv) were added. The resulting reaction mixture was heated until reflux for 18 h. Subsequently, the mixture was allowed to reach room temperature, treated with water (30 mL) and extracted with EtOAc (3 x 25 mL). The combined organic layers were washed with brine (20 mL), dried over Na_2SO_4, filtered and concentrated under reduced pressure to give an orange oil. The desired products were obtained as pale yellow solids (**10**: 82.4 mg, 0.20 mmol, 60%; **11**: 30.5 mg, 0.06 mmol, 54%) by purification *via* gradient column chromatography (SiO_2, *n*-hexane/EtOAc 2:1 – 1:1 – 1:2).

General procedure for the synthesis of artesunic acid-quinazoline hybrids 8 and 9

A solution of artesunic acid (2 equiv) and DMAP (60 mol%) in dry CH_2Cl_2 (3.00 mL) was cooled to 0 °C. Quinazoline alcohol **10** (47.0 mg, 0.11 mmol, 1 equiv) or **11** (29.0 mg, 0.06 mmol, 1 equiv) was added to the reaction mixture at 0 °C under N_2. After addition of DCC (2 equiv), the reaction mixture was slowly warmed to room temperature and stirred overnight (20 h). The precipitated dicyclohexylurea was removed by filtration and the solvent was removed under reduced pressure. The residue was purified by gradient column chromatography (SiO_2, *n*-hexane/EtOAc 4:1, 2:1) and thereby the desired hybrid **8** (69.1 mg, 0.09 mmol, 78%) or **9** (40.2 mg, 0.05 mmol, 81%) was obtained as an off-white solid.

Characterization of artesunic acid-quinazoline hybrids 8/9 and their precursors 10/11

4-((3-hydroxypropyl)amino)-5,7-bis(4-fluorophenyl)quinazoline-8-carbonitrile 10

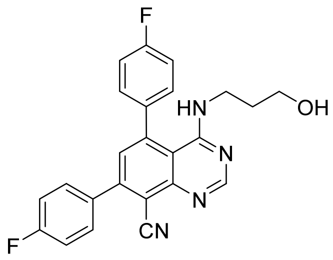

60% yield; ^{1}H NMR (300 MHz, CD_2Cl_2) δ: 1.54 (quint, J = 5.7 Hz, 2H), 2.82 (t, J = 5.7 Hz, 1H), 3.44-3.57 (m, 4H), 5.45 (t, J = 4.8 Hz, 1H), 7.21-7.35 m, (m, 5H), 7.44-7.51 (m, 1H), 7.64-7.73 (m, 2H), 8.67 (s, 1H); ^{13}C NMR (75 MHz, CD_2Cl_2) δ: 32.2, 38.9, 59.6, 109.9, 112.6, 116.2, 116.5, 116.9, 117.1, 117.2, 130.3, 131.4, 131.5 (2), 131.6, 134.0 (d, J = 3.4 Hz), 135.5 (d, J = 3.7 Hz), 142.6, 149.1, 153.2, 157.3, 160.3, 162.4 (d, J = 10.8 Hz), 165.7 (d, J = 10.0 Hz); ^{19}F NMR (282 MHz, $DMSO_{d6}$) δ: -111.11 (m), -111.74 (m); HRMS (ESI+) exact mass calcd. for $C_{24}H_{19}F_2N_4O$ $[M+H]^+$: 417.1521, found: 417.1511.

4-((3-hydroxypropyl)amino)-5,7-bis(4-(trifluoromethyl)phenyl)quinazoline-8-carbonitrile 11

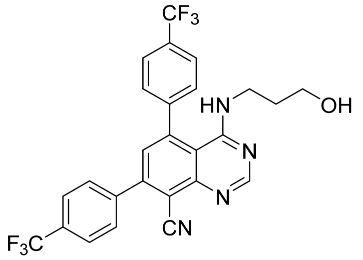

54% yield; ^{1}H NMR (400 MHz, $CDCl_3$) δ: 1.57 (quint, J = 5.6 Hz, 2H), 3.50-3.62 (m, 4H), 5.40 (t, J = 5.2 Hz, 1H), 7.36 (s, 1H), 7.64-7.68 (m, 2H), 7.81-7.90 m, (m, 6H), 8.81 (s, 1H); ^{13}C NMR (75 MHz, $CDCl_3$) δ: 31.3, 39.1, 59.8, 110.7, 111.9, 116.0, 121.9 (d, J = 15.2 Hz), 125.40, 125.60, 126.2 (dq, J_1 = 3.4 Hz, J_2 = 31.2 Hz), 129.1, 129.4 (d, J = 2.4 Hz), 131.6, 131.9, 132.0, 132.3, 140.4, 141.5, 142.5, 148.3, 152.8, 157.3, 159.4; ^{19}F NMR (282 MHz, $CDCl_3$) δ: -60.80, -60,82; HRMS (ESI+) exact mass calcd. for $C_{26}H_{19}F_6N_4O$ $[M+H]^+$: 517.1458, found: 517.1458.

Artesunic acid-quinazoline hybrid 8

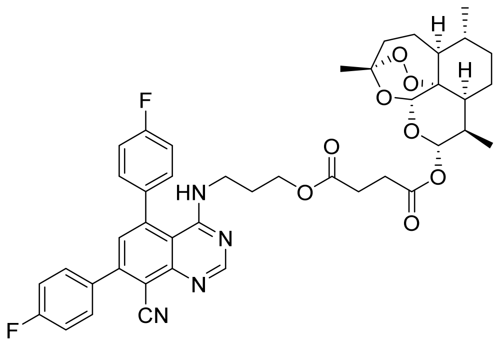

78% yield; ^{1}H NMR (300 MHz, $CDCl_3$) δ: 0.82 (d, J = 7.2 Hz, 3H), 0.94 (d, J = 6.0 Hz, 3H), 0.96-1.08 (m, 1H), 1.18-1.51 (m, 8H), 1.52-1.80 (m, 4H), 1.81-1.94 (m, 1H), 1.95-2.07 (m, 1H), 2.34 (td, J_1 = 13.5 Hz, J_2 = 3.9 Hz, 1H), 2.47-2.62 (m, 3H), 2.63-2.73 (m, 2H), 3.43 (q, J = 5.7 Hz, 2H), 3.92 (t, J = 6.0 Hz, 2H), 5.28 (t, J = 4.5 Hz, 1H), 5.39 (s, 1H), 5.75 (d, J = 9.9 Hz, 1H), 7.15-7.34 (m, 5H), 7.39-7.49 (m, 2H), 7.63-7.69, (m, 2H), 8.76 (s, 1H); ^{13}C NMR (75 MHz, $CDCl_3$) δ: 12.0, 20.2, 22.0, 24.6, 25.9, 27.7, 28.8, 29.1, 31.8, 34.1, 36.2, 37.3, 38.2, 45.3, 51.6, 61.6, 80.1, 91.5, 92.3, 104.5, 109.8, 111.8, 115.9, 116.2, 116.5, 116.6, 116.9, 129.5, 130.6, 130.7, 130.9, 131.0, 133.1 (d, J = 3.2 Hz), 135.0 (d, J = 3.6 Hz), 141.8, 148.7, 152.7, 157.1, 159.4, 162.0, 165.1, 171.0, 172.0; ^{19}F NMR (282 MHz, $DMSOd_6$) δ:

-109.74 (m), -110.70 (m); HRMS (ESI+) exact mass calcd. for $C_{43}H_{45}F_2N_4O_8$ $[M+H]^+$: 783.3200, found: 783.3191; anal. calcd. for $C_{43}H_{44}F_2N_4O_8$: C, 65.97; H, 5.67; N, 7.16; Found: C, 66.51; H, 6.15; N, 6.60.

Artesunic acid-quinazoline hybrid 9

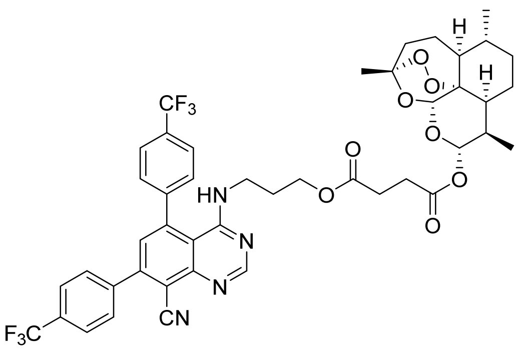

81% yield; ^1H NMR (300 MHz, $CDCl_3$) δ: 0.80 (d, J = 7.2 Hz, 3H), 0.93 (d, J = 5.7 Hz, 3H), 0.97-1.07 (m, 1H), 1.16-1.50 (m, 8H), 1.51-1.78 (m, 4H), 1.79-2.04 (m, 2H), 2.33 (td, J_1 = 14.4 Hz, J_2 = 3.3 Hz, 1H), 2.46-2.58 (m, 3H), 2.59-2.71 (m, 2H), 3.41 (q, J = 6.0 Hz, 2H), 3.87 (td, J_1 = 6.3 Hz, J_2 = 2.1 Hz, 2H), 5.13 (t, J = 5.4 Hz, 1H), 5.37 (s, 1H), 5.72 (d, J = 9.9 Hz, 1H), 7.30 (s, 1H), 7.61-7.65 (m, 2H), 7.72-7.90, (m, 6H), 8.76 (s, 1H); ^{13}C NMR (75 MHz, $CDCl_3$) δ: 12.0, 20.1, 21.9, 24.5, 24.9, 25.6, 25.9, 27.6, 28.6, 29.0, 31.7, 33.9, 34.0, 36.1, 37.2, 38.2, 45.1, 49.1, 51.5, 61.3, 80.1, 91.5, 92.2, 104.4, 110.5, 111.9, 116.1, 125.4, 125.5, 125.9 (q, J = 3.6 Hz), 126.4-126.6 (m), 128.9, 129.3, 129.4, 131.7, 131.8, 132.1, 140.3, 141.5, 142.4, 148.1, 152.7, 156.7, 157.4, 159.2, 171.0, 172.0; ^{19}F NMR (282 MHz, $CDCl_3$) δ: -63.19, -63,27; HRMS (ESI+) exact mass calcd. for $C_{45}H_{45}F_6N_4O_8$ $[M+H]^+$: 883.3136, found: 883.3134; anal. calcd. for $C_{45}H_{44}F_6N_4O_8$: C, 61.22; H, 5.02; N, 6.35; found: C, 61.82; H, 5.64; N, 6.04.

Supplementary references

1. Fröhlich T, Ndreshkjana B, Muenzner JK, Reiter C, Hofmeister E, Mederer S, Fatfat M, El-Baba C, Gali-Muhtasib H, Schneider-Stock R, Tsogoeva SB. Synthesis of Novel Hybrids of Thymoquinone and Artemisinin with High Activity and Selectivity Against Colon Cancer. *ChemMedChem* **12**, 226-234 (2017).

2. Marschall M, Freitag M, Weiler S, Sorg G, Stamminger T. Recombinant GFP-expressing human cytomegalovirus as a tool for screening of antiviral agents. *Antimicrob. Agents Chemother.* **44**, 1588-1597 (2000).

3. Hutterer C, Niemann I, Milbradt J, Fröhlich T, Reiter C, Kadioglu O, Bahsi H, Zeitträger I, Wagner S, Einsiedel J, Gmeiner P, Vogel N, Wandinger S, Godl K, Stamminger T, Efferth T, Tsogoeva SB, Marschall M. The broad-spectrum antiinfective drug artesunate interferes with the canonical nuclear factor kappa B (NF-kB) pathway by targeting RelA/p65. *Antiviral Res.* **124**, 101-109 (2015).

4. Hutterer C, Eickhoff J, Milbradt J, Korn K, Zeitträger I, Bahsi H, Wagner S, Zischinsky G, Wolf A, Degenhart C, Unger A, Baumann M, Klebl B, Marschall M. A novel CDK7 inhibitor of the pyrazolo-triazine class exerts broad-spectrum antiviral activity at nanomolar concentrations. *Antimicrob. Agents Chemother* **59**, 2062-2071 (2015).

5. Williams ATR, Winfield SA, Miller JN. Relative fluorescence quantum yields using a computer-controlled luminescence spectrometer. *The Analyst* **108**, 1067- 1071 (1983).

6. Hamai S, Hirayama F. Actinometric determination of absolute fluorescence quantum yields. *J. Phys. Chem.* **87**, 83-89 (1983).

7. Hutterer C, Hamilton S, Steingruber M, Zeitträger I, Bahsi H, Thuma N, Naing Z, Örfi Z, Örfi L, Socher E, Sticht H, Rawlinson W, Chou S, Haupt VJ, Marschall M. The chemical class of quinazoline compounds provides a core structure for the design of anticytomegaloviral kinase inhibitors. *Antiviral Research* **134**, 130-143 (2015).

8. Hutterer C, Wandinger SK, Wagner S, Müller R, Stamminger T, Zeitträger I, Godl K, Baumgartner R, Strobl S, Marschall M. Profiling of the kinome of cytomegalovirus-infected cells reveals the functional importance of host kinases Aurora A, ABL and AMPK. *Antiviral Research* **99**, 139-148 (2013).

9. Karmarkar SN, Kelkar SL, Wadia M. A simple unusual one-step conversion of aromatic aldehydes into nitriles. *Synthesis*, 510-512 (1985).

10. Ying A, Xu S, Liu S, Ni Y, Yang J, Wu C. Novel multiple-acidic ionic liquids: Catalysts for environmentally friendly benign synthesis of trans-*β*-nitrostyrenes under solvent-free conditions. *Industrial & Engineering Chemistry Research* **53**, 547-552 (2014).

11. Lopchuk JM, Hughes RP, Gribble GW. What controls regiochemistry in 1,3-dipolar cycloadditions of munchnones with nitrostyrenes? *Org Lett.* **15**, 5218-5221 (2013).
